# Comparative Transcriptome Analysis of Chinary, Assamica and Cambod tea (*Camellia sinensis*) Types during Development and Seasonal Variation using RNA-seq Technology

**DOI:** 10.1038/srep37244

**Published:** 2016-11-17

**Authors:** Ajay Kumar, Vandna Chawla, Eshita Sharma, Pallavi Mahajan, Ravi Shankar, Sudesh Kumar Yadav

**Affiliations:** 1 Plant Metabolic Engineering Laboratory, Biotechnology Division, CSIR-Institute of Himalayan Bioresource Technology, Palampur-176061 (HP), India; 2 Academy of Scientific and Innovative Research, New Delhi, India; 3 Studio of Computational Biology & Bioinformatics, Biotechnology Division, CSIR-Institute of Himalayan Bioresource Technology, Palampur-176061 (HP), India; 4 Department of Biotechnology, Guru Nanak Dev University, Amritsar, Punjab, India; 5 Food and Nutraceutical Division, CSIR-Institute of Himalayan Bioresource Technology, Palampur-176061 (HP), India; 6 Center of Innovative and Applied Bioprocessing (CIAB), Mohali-160071, Punjab, India

## Abstract

Tea quality and yield is influenced by various factors including developmental tissue, seasonal variation and cultivar type. Here, the molecular basis of these factors was investigated in three tea cultivars namely, Him Sphurti (H), TV23 (T), and UPASI-9 (U) using RNA-seq. Seasonal variation in these cultivars was studied during active (A), mid-dormant (MD), dormant (D) and mid-active (MA) stages in two developmental tissues viz. young and old leaf. Development appears to affect gene expression more than the seasonal variation and cultivar types. Further, detailed transcript and metabolite profiling has identified genes such as F3′H, F3′5′H, FLS, DFR, LAR, ANR and ANS of catechin biosynthesis, while MXMT, SAMS, TCS and XDH of caffeine biosynthesis/catabolism as key regulators during development and seasonal variation among three different tea cultivars. In addition, expression analysis of genes related to phytohormones such as ABA, GA, ethylene and auxin has suggested their role in developmental tissues during seasonal variation in tea cultivars. Moreover, differential expression of genes involved in histone and DNA modification further suggests role of epigenetic mechanism in coordinating global gene expression during developmental and seasonal variation in tea. Our findings provide insights into global transcriptional reprogramming associated with development and seasonal variation in tea.

Tea (*Camellia sinensis* L.) of *Theaceae* family is an evergreen perennial tree species distributed all across the tropics and subtropics region^1^. Tea enjoys the status of being the most widely consumed drink, next only to water. It is an important commercial crop with diverse health beneficial properties. Commercially used tea is mainly distributed in three major varieties *Camellia sinensis* (Chinary), *Camellia assamica* (Assamica) and *Camellia assamica ssp lasiocalyx* (Cambod)^2^. Young tissues (an apical bud and the associated two leaves) of these varieties are used to manufacture high quality teas^3^. Tea cultivars type season and tissue were known to influence yield and quality of tea. Apical bud growth rate in tea is maximum during active growth season from April to September and minimum during winter dormancy from October to March, thus severely reducing the commercial yield of tea^4^,^5^. Understanding the mechanism of regulation of quality tea production is of immense commercial and health interest. Therefore, identification of genes associated with development and seasonal variation can greatly facilitate the development of improved tea cultivars with enhanced tolerance using biotechnological approaches.

Development in terms of vegetative growth and seasonal variations are controlled by regulatory network of genes. The mechanisms pertaining to these variations could be attributed to the underlying genetic or epigenetic regulations. However, in tea, influence of season and development has been shown only on a few selected genes^4^,^5^,^6^,^7^,^8^. Due to non-availability of the reference genome sequence in tea, informations generated are not comprehensive. Recently, attempts have been made to identify transcriptional regulation using transcriptomic approach in tea^1^,^9^,^10^,^11^. However, these studies are limited to either specific cultivar and/or seasons. Moreover, there are still major gaps in understanding molecular mechanisms governing transition to and release from dormancy. In addition, seasonal variation is also known to activate a range of physiological responses including phytohormonal regulations and epigenetic mechanisms^12^,^13^. The relative expression of genes related to plant hormone metabolism and signal transduction such as ABA, gibberellins, ethylene, brassinosteroid and auxin have been described in other perennial plant species during vegetative growth and seasonal variations^14^,^15^. However, transcriptional regulation of phytohormone biosynthesis/catabolism, signal transduction and epigenetic mechanism has not been well understood in tea plant. Thus, comparing tea cultivars differing in yield, quality and environmental sensitivities could represent a comprehensive approach in delineating the molecular mechanisms of underlying tolerance and provide better candidate genes for key metabolic pathways.

Here, we report transcriptome analysis of three tea cultivars for better understanding of the molecular mechanisms controlling developmental and seasonal variations. Our data emphasizes several characteristic transcriptional changes pertaining to initiation, progression and subsequent release of dormancy accompanying active growth in three different tea cultivars. Further, transcript and metabolite profiling revealed the molecular regulation of catechins and caffeine pathways during development and seasonal variation in three cultivars. Present data would render better understanding of the mechanisms involving development and growth vis-a-vis seasonal variations in tea cultivars of commercial importance.

## Results

### Reads generation, *de novo* assembly and homology search

High-throughput sequencing of tea transcriptome with PE run of 2 × 72 cycles for each of 24 samples was resulted into a total of 271,873,512 paired-end reads and on pre-processing, total reads were reduced to 213,184,738 representing 74,048,716 (78.154%), 78,409,904 (77.818%) and 60,726,118 (79.521%) quality reads for Him Sphurti (H), TV23 (T) and UPASI-9 (U) cultivars, respectively (Table 1; Supplementary Fig. S1). Details of various tea samples used in this study for sequencing are provided in Table 1.

**Figure 1:**
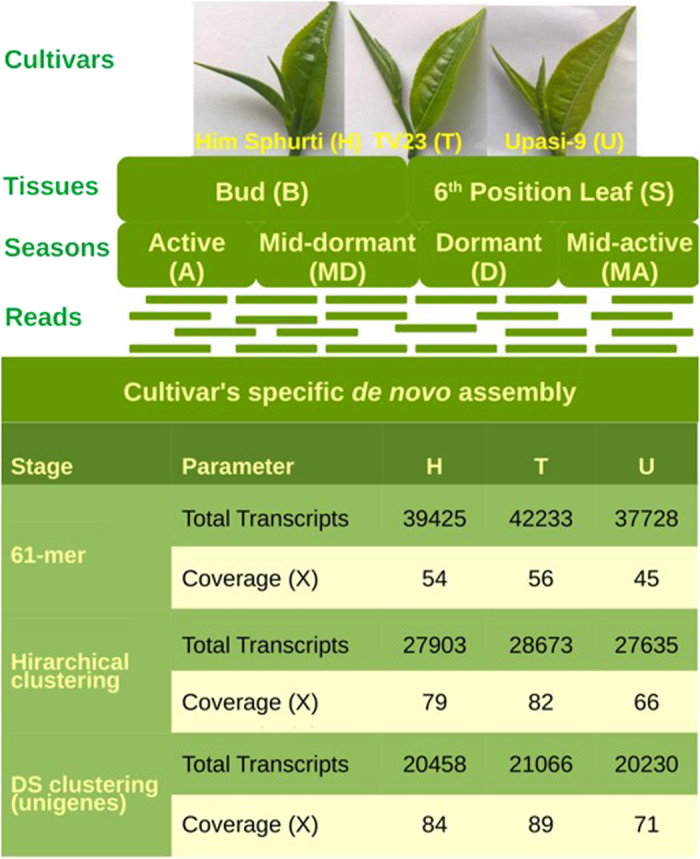


From all the assemblies resultant of different *K*-mers (21–69 mers) (Supplementary Table S1), 61 *K*-mer size was selected as most appropriate for each cultivar specific assembly. Minimum length cut-off for assembled transcripts was set to 100 bases. The total transcript sequences obtained after primary assembly steps were 39,425 (34.73%) for ‘H’, 42,233 (31.38%) for ‘T’ and 37,728 (32.99%) for ‘U’ cultivar with transcripts having length above 1000 bases. The maximum length of the transcripts was found to be 42,142, 40,124 and 7,985 bases, while the average transcript length was ~896.64, 828.71 and 840.93 bases having 1634, 1587, 1517 bases as N50 value and coverage of 54.21x, 56.26x and 45.871x for ‘H’, ‘T’ and ‘U’, respectively (Fig. 1; Supplementary Table S1). Different level of transcripts’ clustering and selections steps resulted into reduction of total number of transcripts to 20,458, 21,066 and 20,230 for best group representation (unigenes) in ‘H’, ‘T’ and ‘U’, respectively (Fig. 1). The details of clustered sequences are provided as Supplementary Table S1. In cultivars’ specific unigenes, transcripts above 1000 bases were 45.28%, 44.98% and 44.68% in ‘H’, ‘T’ and ‘U’ cultivars, respectively. The unigenes were resulted into 1,578, 1,561 and 1,513 bases as N50 value with coverage of 84.24X, 89.59X and 71.30X in ‘H’, ‘T’ and ‘U’, respectively (Supplementary Table S1). Among the total assembled sequences 27,903, 28,673 and 27,635 for ‘H’, ‘T’ and ‘U’, respectively, a total of 12401, 12,506 and 12,406 sequences had similarity with ESTs (Supplementary Table S2).

### Functional annotation and classification

Assignment of corresponding functional categories viz. Gene Ontology [Biological Process (P), Molecular Function (F) & Cellular Component (C)], Enzyme Classification (EC) and Kyoto Encyclopedia of Genes and Genomes (KEGG) were based on highest scoring hits attained for the sequences using Annot8r tool. Annotation of tea unigenes against GO database was resulted in significant hits for 16,778 transcripts in ‘H’ (P = 16,249, F = 15,253, C = 16,066), 17,270 transcripts in ‘T’ (P = 16,777, F = 15677, C = 16541) and 16,613 transcripts in ‘U’ (P = 16,088, F = 15,149, C = 15,936). Annotation of unigenes for EC was resulted into a total of 9,194 transcripts in ‘H’, 9,515 transcripts in ‘T’ and 9,110 transcripts in ‘U’ while for KEGG category 9,592 transcripts in ‘H’, 9,902 transcripts in ‘T’, and 9,491 transcripts in ‘U’ (Supplementary Fig. S2). General annotation details of all the unigenes are listed in Supplementary Table S3.

### Global expression profiling of three different tea cultivars during development and seasonal variation

**Figure 2:**
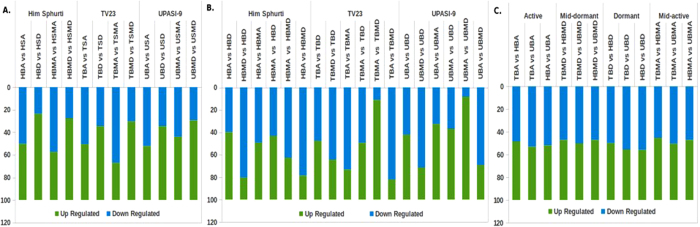
Differential gene expression in different tea cultivars during development and seasonal variation. (**A**) Number of differentially expressed genes in different tea cultivars during development at four stages of season are presented in the bar graph. (**B**) Number of differentially expressed genes in different tea cultivars during seasonal variations are presented in the bar graph. (**C**) Number of differentially expressed genes among three tea cultivars at four stages of season are presented in the bar graph. Number of up-and down-regulated genes are presented via the bars along the Y-axis.

We explored the transcriptome of twenty four libraries from 24 samples to identify differentially expressed genes (DEGs) during development and seasonal variations among three tea cultivar. The transcripts with log two-fold and above differential expressions were selected as DEGs. FPKM expression value and differential expression of all unigenes specific to three cultivars: ‘H’, ‘T’ and ‘U’ during developmental and seasonal variations are listed in Supplementary Table S4. MA-plot representing differences between the two samples while plotting logarithmic fold changes on the y-axis against the logarithmic mean of counts on the x-axis (Supplementary Fig. S3). Tissues from three cultivars were compared at four stages of season to identified DEGs among cultivars. The number of DEGs varied from 12064 (for TBD_UBD) to 17427 (for TBA-TBA). All three cultivars showed higher number of DEGs at active season (A). A total of 17,427 (TBA_HBA), 16887 (TBA_UBA) and 16497 (UBA_HBA) Transcripts were identified as DEGs at ‘A’ season. However, highest number of transcripts 8971 (TBA_UBA) and 7784 (TBMA_UBMA) were upregulated at ‘A’ and mid-active (MA) stages, while 7059 (TBMD_UBMD), 7151 (TBD_HBD) were upregulated at mid-dormant (MD) and dormant (D) stages respectively, (Fig. 2; Supplementary Table S5). Young and old tissue from three cultivars were compared at four different stages of season to identify DEGs during development. The number of DEGs varied from 823 (for HBMA_HSMA) to 3704 (for TBA_TSA) during development. Highest DEGs were observed at ‘A’ season that decreased subsequently at ‘MD’ and ‘D’ seasons. During development, larger number of transcripts were downregulated at ‘A’ and ‘MA’ season, while at ‘MD’ and ‘D’ season higher number of transcripts were upregulated in all three cultivars (Fig. 2; Supplementary Table S5). Further, DEGs were identified during seasonal variations by comparing young tissues collected at ‘A’, ‘MD’, ‘D’ and ‘MA’ seasons from three cultivars. During Seasonal, variation number of DEGs varied from 29 (TBA_TMBA) to 1986 (TBA_TBD) in ‘T’, 93 (HBA_TMBA) to 1468 (HBA_HBD) in H and 95 (UBMA_UBMD) to 2440 (UBA_UBD) in ‘U’ cultivar. The higher number transcripts were found upregulated 1064 (TBA_TBD), 883 (HBA_HBD), and 1410 (UBA_UBD) when tissues from ‘A’ and ‘D’ season were compared (Fig. 2; Supplementary Table S5).

### Gene Ontology (GO) and Kyoto Encyclopedia of Genes and Genomes (KEGG) enrichment analysis of differentially expressed genes (DEGs)

We performed GO and KEGG enrichment analysis to assign functional categories to the differentially expressed genes (DEGs) during developmental and seasonal comparisons. In GO enrichment analysis, GO term related to ‘biological process’ category such as cell proliferation, development growth, regulation of meristem development and maintenance, secondary metabolic processes, hormone metabolic process, regulation of gene expression, epigenetic, photosynthesis, DNA methylation, cell cycle, multi-organism process and response to abiotic stimulus were highly enriched during development and seasonal variations (Supplementary Table S6; Supplementary Fig. S4). On the other hand, GO term related to ‘molecular function’ such as NADH-dehydrogenase, various aspects of oxidoreductase, hydrolase activity, transporter activity and DNA and protein binding were highly enriched during development and seasonal variations (Supplementary Table S6; Supplementary Fig. S4).

In KEGG pathway enrichment analysis 55, 61, and 58 pathways corresponding to 3670, 5679 and 5520 DEGs were significantly enriched in ‘H’, ‘T’ and ‘U’, respectively (P ≤ 0.05; Supplementary Table S7). The genes that encode enzymes involved in various metabolic pathways such as pentose and glucuronate interconversion pathway, galactose metabolism were highly enriched during seasonal variation in all three cultivars. However, starch and sucrose metabolic pathway was additionally enriched during different developmental stages in ‘H’ and ‘U’ cultivars. Pathways involved in secondary metabolites biosynthesis such as flavonoids and phenylpropanoids, were enriched in young tissues during ‘A’ and ‘MA’ seasons. Moreover, pathways corresponding to the circadian rhythm, photosynthesis, plant hormones signal transduction was found to be significantly enriched during developmental and seasonal variations (Supplementary Table S7).

### Transcriptome and metabolite analysis identified molecular response of key secondary metabolic pathways during development and seasonal variation in three tea cultivars. Catechin biosynthetic pathway

**Figure 3:**
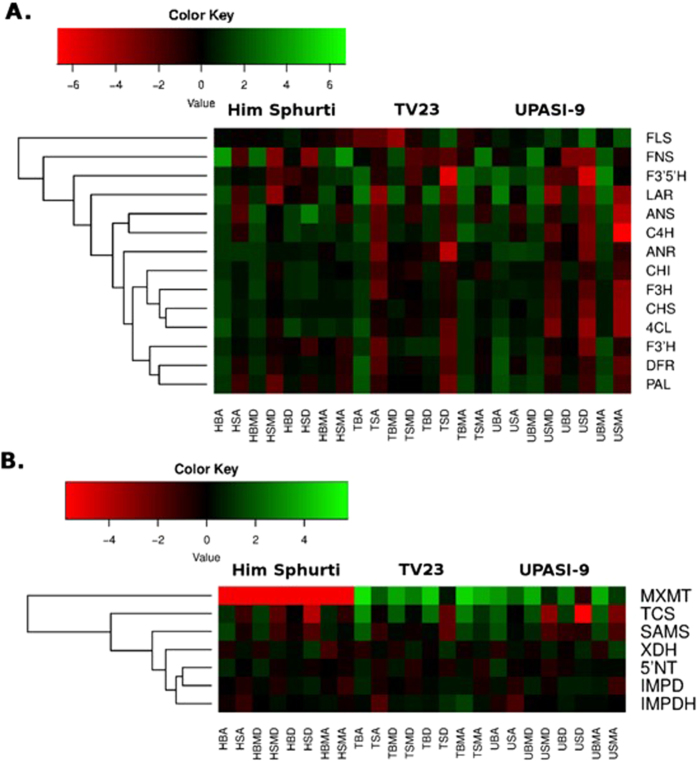
Regulation of phenylpropanoid and caffeine pathway in three tea cultivars during development and seasonal variation. Heatmaps showing expression profile of transcripts annotated in phenylpropanoid (**A**) and caffeine pathway (**B**). The color scale represents log2 transformed FPKM value.

The flavonoid biosynthetic pathway of *C. sinensis* has been studied at physiological, biochemical and genetic levels^16^,^17^,^18^. Although, several independent studies have reported the expression profile of various genes of these pathways individually or in limited number in response to leaf age and different external cues in single varieties^19^,^20^,^21^,^22^,^23^,^24^,^25^. Also, these previous studies were limited either by gene studied, tissues, variety and/or season. In view of this, expression profile of key regulatory gene of flavonoid pathway was comprehensively investigated in three tea cultivars during development and seasonal variation. Among the cultivars eight gene namely, flavanone 3-hydroxylase (*F3H*), flavonoid 3′-hydroxylase (*F3′H*), flavonoid 3′,5′-hydroxylase (*F3*′*5*′*H*), flavonol synthase (*FLS*), dihydroflavonol 4-reductase (*DFR*), leucoanthocyanidin reductase (*LAR*), flavone synthase (*FNS*) and anthocyanidin reductase (*ANR*) were differentially expressed. In particular, the expression of F3′H, F3′5′H, FLS, DFR, LAR and ANR was found to be highest in ‘U’ and ‘T’ cultivars whereas F3H and FNS was highest in ‘H’ cultivar. In addition, expression of phenylalanine ammonia-lyase (*PAL*), chalcone isomerase (*CHI*), 4-coumarate CoA ligase (*4CL*), cinnamate 4-hydroxylase (*C4H*), chalcone synthase (*CHS*) and *F3H* gene was not affected at different season in ‘H’ cultivar. Results also showed minimum variation in gene expression as well as metabolite content in ‘H’ cultivar across the season and tissue as compared to ‘U’ and ‘T’ cultivars (Fig. 3A; Supplementary Table S8A). Further, expression analysis indicated that almost all known genes related to flavonoid biosynthesis were differentially expressed during development. The expressions of all genes were observed to be higher in young tissue during development at four different stages of season except FLS, whose expression was observed in old leaf tissue (Fig. 3A; Supplementary Table S8A). However, minimum difference in expression was observed during development at ‘D’ season. Moreover, while comparing seasons, the tissues obtained during ‘A’ season showed maximum relative expression of most of the regulatory genes except F3H, 4CL, C4H, CHS and CHI, intended to decrease in subsequent seasons and observed minimum expression for these genes during ‘D’ season which subsequently increased during ‘MA’ season. (Fig. 3A; Supplementary Table S8A).

Previously, variation in catechins concentration has been reported during one or two seasons in tea^19^. Here, influence of cultivar type, development stage and season on catechins content was identified by analysing young and old leaf tissue of three tea cultivars during four different seasons using HPLC. Six characteristic tea catechins namely; catechin (C), epicatechin (EC), gallocatechin (GC), epigallocatechin (EGC), epicatechingallate (ECG), epigallocatechingallate (EGCG) showed significant differences in their content among three tea cultivars. The highest total catechins (TC) content was recorded in ‘U’ (26.9%) followed by ‘T’ (23.9%) and ‘H’ (19.8%) (Table 2). The difference was also observed in two different tissues. TC was higher in young compared to old leaf tissue. Data on TC during seasonal variation has documented its highest content during active season and lowest during dormant season. Among the individual catechins, all six catechins were recorded in higher amount in young tissue during ‘A’ season. EGCG was recorded in highest amount followed by ECG, while GC was lowest in all three cultivars irrespective of tissue and season. The order of catechins was EGCG > ECG > EC > EGC in ‘H’ cultivar whereas in ‘T’ and ‘U’ cultivar was EGCG > ECG > EGC > EC (Table 2). Hence, cultivar-type, their tissue development stage and seasons have strong influence on the catechins level in tea.

### Caffeine biosynthetic pathway

Transcriptome analysis identified expression profile of genes related to caffeine biosynthesis including IMP dehydrogenase (IMPDH), AMP deaminase (AMPD), 5′-Nuleotidase (5′NT), S-adenosyl-L-methionine synthase (SAMS), theobromine synthase (7-methylxanthine-N-methyltransferase) (MXMT), caffeine synthase (TCS) in young and old leaf tissues of ‘H’, ‘T’ and ‘U’ tea cultivars during ‘A’, ‘MD’, ‘D’ and ‘MA’ seasons. The unigene IDs of these genes are listed in Supplementary S8A. Simultaneously, the hierarchical clustering analysis showed that expression of IMPDH, AMPD and 5′NT genes were not varied with tissue, season and cultivar (Fig. 3B; Supplementary Table S8A). Among cultivars, TCS and MXMT expression was highest in ‘T’ cultivar fallowed by ‘U’ and ‘H’ cultivars whereas SAMS expression was highest in ‘U’ cultivar followed by ‘H’ and ‘T’ cultivar. Unexpectedly no expression was observed for MXMT in ‘H’ cultivar (Fig. 3B; Supplementary Table S8A). The expression of TCS, MXMT and SAMS was observed to be higher in young tissue during development. While during seasonal variation the expressions of these genes were highest during ‘A’ season and intended to decrease during ‘MD’ and ‘D’ and subsequently increased during MA season. In addition, gene encoding xanthine dehydrogenase (XDH), an enzyme involved in xanthine (precursor of caffeine) catabolism showed higher expression in old tissue during development; while during seasonal variation highest expression was observed at ‘D’ and ‘MD’ seasons. Interestingly, expression of XDH gene was not affected by cultivars (Fig. 3B; Supplementary Table S8A).

Among the cultivars caffeine content was highest in ‘H’ (4.4%) followed by ‘T’ (3.6%) and ‘U’ (3.4%). However, lowest caffeine content was observed in old tissue of ‘U’ (0.75%) cultivar at ‘D’ season (Table 2). Caffeine content was found to be higher in young tissue during development in all three cultivars irrespective of seasons (Table 2). Caffeine content was recorded maximum during ‘A’ season and minimum during ‘D’ season in all three tea cultivars (Table 2).

Further, correlation was identified for total catechin (TC) and caffeine content to expression of important genes of catechin and caffeine biosynthesis mentioned above in three tea cultivars during development and seasonal variation. Relative expressions of PAL, 4CL, C4H, CHS, CHI, F3′H, F3′5′H, LAR, DFR, and ANS gene were positively correlated with TC during ‘A’ and ‘MA’ season (Table 3). Moreover, expression of 4CL, C4H, CHS, FNS, LAR, DFR and ANS was also positively correlated with TC content during ‘MD’ season (Table 3). While during ‘D’ season the expression of only PAL and FNS genes were positively correlated with TC content (Table 3). Moreover, in caffeine biosynthesis pathway significant positive correlation was observed for SAMS and TCS gene expression with caffeine content during ‘A’, ‘MD’ and ‘MA’ seasons (Table 3). However, the expression of XDH showed negative correlation with caffeine content during ‘A’, ‘MD’ and ‘MA’ season (Table 3).

Data on catechin and caffeine contents were analyzed by two-way ANOVA followed by Duncun’s test to explore the effects of cultivar, season, tissue, cultivar*season, cultivar*tissue, season*tissue and cultivar*season*tissue in tea plants. Significant differences in individual and total catechin and caffeine concentrations were identified in three tea cultivars during development and seasonal variation (Table 4). Results indicated that different tissue, cultivars and seasons have effect on catechin and caffeine accumulation in tea plants.

### Transcriptional response of phytohormone biosynthesis and signal transduction related genes during development and seasonal variation

**Figure 4:**
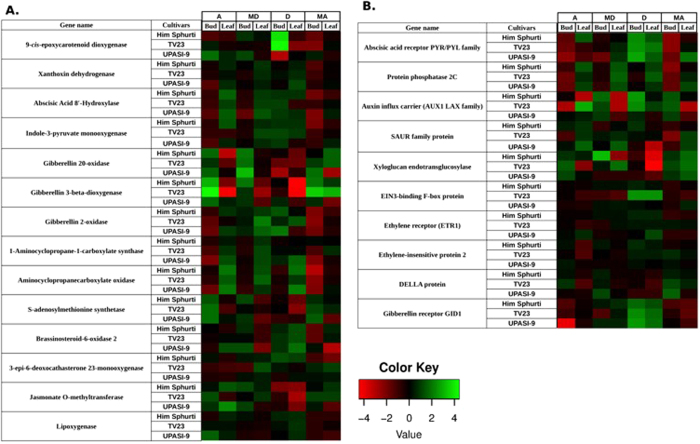
Comparative expression profile of genes related to phytohormone metabolism and signal transduction during development and seasonal variation in tea cultivars. Heatmaps showing differential expression of genes annotated to ABA, gibberellins, ethylene, auxin and brasinosteroid metabolism (**A**) and signal transduction (**B**). The color scale at the bottom represents log2 transformed FPKM value.

The DEGs annotated to phytohormone metabolism in KEGG enrichment analysis were further investigated to identify transcriptional regulation of various phytohormones during development and seasonal variations in three tea cultivars. The enrichment of carotenoid pathway, associated with ABA metabolism was observed (Supplementary Table S7). The ABA biosynthetic pathway genes such as 9-cisepoxycarotenoid dioxygenase (NCED) and zeaxanthin epoxidase showed higher expression in old tissue compared to young tissue during development. While during seasonal variations, expression of these genes were observed to be higher at ‘D’ and ‘MD’ seasons in ‘H’ and ‘T’ cultivars respectively. Conversely, in the ‘U’ cultivar expression of NCED gene was higher during ‘A’ and ‘MA’ seasons (Fig. 4A; Supplementary Table S9). However the transcripts of genes encoding ABA8′-hydroxylase, a key enzyme for ABA degradation^26^, showed higher expression during ‘D’. Similarly differential expression of gibberellin metabolism-related genes was observed by analyzing the diterpenoid biosynthesis pathway (Supplementary Table S7). The expression of transcripts annotated as GA20 oxidase (GA20ox), a key enzyme of active gibberellins’ biosynthesis was higher in young tissue compared to old tissue during ‘A’ season in all three tea cultivars. However, the highest expression of this gene was noticed during ‘MD’ season only. Conversely, expression of the gene encoding gibberellin 2-beta-hydroxylase, an enzyme involved in gibberellin inactivation showed higher expression in old tissue compared to young tissue during ‘D’ and ‘MD’ seasons (Fig. 4A).

The cysteine and methionine metabolic pathway, related to ethylene biosynthesis was found to be significantly enriched in KEGG analysis (Supplementary Table S7). Genes related to ethylene biosynthesis such as S-adenosyl-L-methionine synthetase (SAMS), ACC synthase (ACS) and ACC oxidase (ACO) were differentially expressed. The expressions of ACS and ACO genes were higher in old tissue during ‘D’ and ‘MD’ seasons in ‘T’ and ‘U’ cultivar. However in ‘H’ cultivar, expression of these genes was higher in young tissue during ‘A’ and ‘MA’ season (Fig. 4A). Moreover, the expression SAMS was higher in young tissue during ‘A’ and ‘MA’ season. Further, differential expressions of genes involved in other phytohormone pathways were also investigated. Indole-3-pyruvate monooxygenase gene or YUCCA, which catalyzes the rate limiting step in the auxin biosynthesis pathway^27^, was up-regulated in old tissue during ‘D’ and ‘MD’ seasons irrespective of cultivars (Fig. 4A; Supplementary Table S9). Brassinosteroid-6-oxidase2 and CYP90D1 related to brassinosteroid biosynthesis were up-regulated during ‘D’ and ‘MD’ season (Fig. 4A; Supplementary Table S9). Gene related to jasmonate biosynthesis such as linoleate 13S-lipoxygenase was down regulated in young tissue during ‘A’ season in ‘H’ and ‘T’ cultivar. Conversely, this gene was up-regulated in ‘U’ cultivar. Jasmonate-O-methyltransferase, whose product deactivates jasmonate, was up-regulated in young tissue at ‘A’ season in all three cultivars. However, an increase in expression of this gene was also observed during ‘MD’ season in ‘H’ and ‘U’ cultivars (Fig. 4A; Supplementary Table S9).

DEGs were also annotated in plant hormone signal transduction pathways (Supplementary Table S7), among which ABA, gibberellins, ethylene, auxin and brassinosteroid were further analyzed (Fig. 4B; Supplementary Table S9). The relative expression of genes involved in ABA signaling pathway such as abscisic acid receptor PYR/PYL family and PROTEIN PHOSPHATASE 2 C (PP2C) was higher in old tissues during ‘D’ and ‘MD’ seasons (Fig. 4B; Supplementary Table S9). In gibberellin-responsive pathway, genes encoding DELLA proteins showed higher expression whereas gibberellin receptor GID1 was lower in young tissues during ‘A’ and ‘MA’ seasons compared to ‘D’ season in all three cultivars. In ethylene signal transduction pathway, genes annotated as ethylene receptor (ETR1) and EIN3-binding F-box protein (EBF1) have shown synchronized expression patterns, with lower levels of ETR transcripts and higher levels of EBF transcripts towards ‘A’ and ‘MA’ seasons (Fig. 4B; Supplementary Table S9). The gene annotated as auxin influx carrier (AUX1 LAX family) was up-regulated in old tissue during ‘D’ and ‘MD’ seasons in all three cultivars. Similar up-regulation of SAUR family protein was observed for ‘H’ and ‘T’ cultivars during ‘D’ and ‘MD’ seasons. SAUR family protein was also up-regulated during ‘A’ season in ‘U’ cultivar. In the brassinosteroid pathway, TCH4 genes involved in brassinosteroid induced cell elongation were up-regulated in young tissue during ‘A’ season (Fig. 4B; Supplementary Table S9).

### Transcriptional response of epigenetic regulation related genes during development and seasonal variation

**Figure 5:**
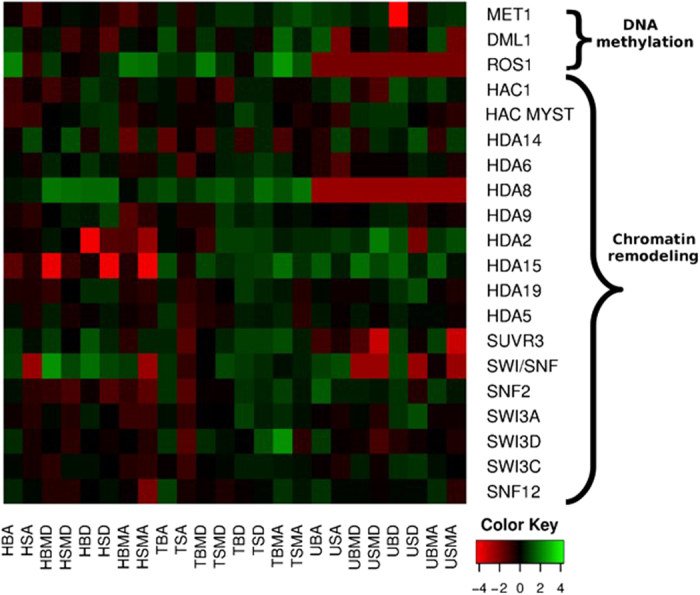
Differential expression of genes associated with epigenetic regulation during development and seasonal variation in tea cultivars. Heatmap showing relative expression of selected genes involved in DNA methylation, histone modification, and chromatin remodeling during development and seasonal variation in tea plant. The color scale represents log 2 transformed FPKM value.

The expression of chromatin remodeling components during dormancy and in subsequent growth reactivation has been conducted in tea (Fig. 5). Dormancy was observed to be inducing the expression of different histone deacetylases, SUVR3 and SWI/SNF**-**like homolog. Also, higher expression of methyltransferase gene and lower expression of DEMETER-like as well as ROS genes was observed during ‘D’ and ‘MD’ seasons in old tissue of three tea cultivars (Fig. 5).

### qRT–PCR validation of differentially expressed transcripts

**Figure 6:**
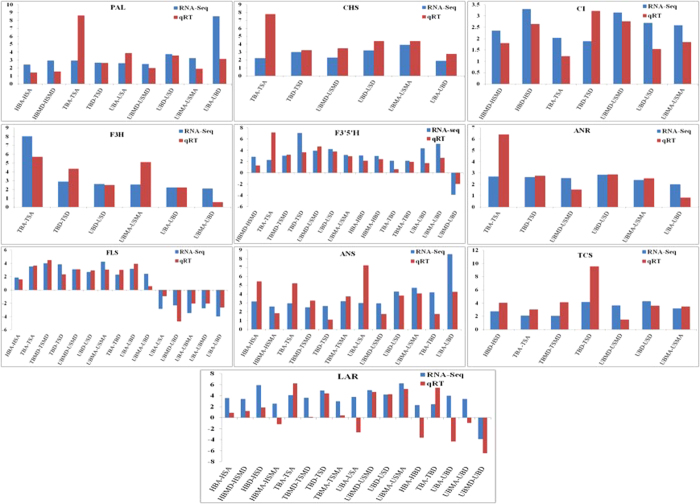
Validation of expression profile of RNAseq through qRT-PCR. A total of 10 genes were selected and validated using qRT-PCR against their expression profile from RNA-seq.

To authenticate the reproducibility as well as accuracy of differential gene expression identified through RNA-seq and computational analysis, 10 genes related to phenylpropanoid, flavonoid and caffeine biosynthesis pathways having differential expression were selected for qRT–PCR. Among the chosen transcripts the expression pattern of 9 genes obtained through qRT-PCR and RNA-seq was in accordance with each other. Bar-graph was plotted by comparing the change in log2 fold values calculated by transcriptome analysis and qRT–PCR (Fig. 6). However, one transcript (LAR) did not match precisely with its RNA-seq value (Fig. 6). Such differences in expression pattern observed by RNA seq and qRT-PCR methods have also been reported by other groups as well^1^,^28^,^29^.

## Discussion

Development (apical bud growth), seasonal variation as well as cultivars are amongst the critical determinants of tea quality and yield. Due to the significance of development, tea plants have evolved sophisticated system to detect environmental conditions and to regulate developmental programs. Recently, Paul *et al.*^9^ have reported the expression profile in assamica type of tea clone during active growth and dormancy. However, the molecular regulations related to tea yield and quality owing to the influence of development, seasonal variations and genetic background remains largely unexplored. Therefore, present investigation targets at generating knowledge about gene networks and molecular regulation of processes that are influenced by development and seasonal variation in three tea cultivars grown in Kangra valley of Himachal Pradesh, India.

Global transcriptional reprogramming is considered as the important molecular response of the plants to adapt to different developmental and season variations. To understand the molecular response, RNA-seq analysis of tea cultivars was performed and investigated the transcriptional differences during development and seasonal variation in three tea cultivars. Identification of several genes and transcript isoforms in tea demonstrated the power of deep sequencing technology. Large number of transcripts exhibited a developmental stage, season and/or cultivar specific response. Differences in number of DEGs in ‘T’ and ‘U’ and ‘H’ cultivars during development and seasonal variation has indicated different response of these cultivars towards such variations. The results suggest that major transition in transcriptome of three tea cultivars occurred during development at ‘A’ season, while it remained more or less quiescent at ‘D’ season. Across seasonal variations, less number of DEGs during ‘D’ season has indicated the down-regulatory effect of dormancy on transcript expression. Such down regulatory effects on transcript number and expression during dormancy has also been reported in assamica type of tea clone^9^. Overall analysis of RNA-seq data revealed a complex transcriptional network governing developmental and seasonal variation in tea.

Catechins are not only important for tea quality but also related to the growth and metabolism of tea plant. The amount of catechins present in the tea shoots gives an indication of the potential of a cultivar to produce good quality tea. Recently, RNA-Seq technology was used to identify key genes involved in the regulation of the flavonoid biosynthesis pathway in tea^11^,^30^. However, only few reports are available on the changes of genes related to catechin biosynthesis in response to environmental stresses^31^,^32^. Here, comparative transcriptome analysis data documented that the expression levels of F3′H, F3′5′H, FLS, DFR, LAR and ANR were highest in ‘T’ and ‘U’ cultivars and supported the consistent changes in EGCG, ECG, and EGC along with TC levels. Results have suggested the key regulatory role of these genes in controlling the levels of catechins in different cultivars. Moreover, higher catechins are reported in larger-leaf tea species^10^. The leaf size of cultivars used in the present study were large, medium and small in size in ‘U’, ‘T’ and ‘H’ cultivar respectively. In agreement with this, highest TC was recorded in ‘U’ cultivar followed by ‘T’ and ‘H’ cultivars which further suggested the potential of ‘U’ cultivar to produce better quality tea compared to ‘T’ and ‘H’ cultivars. Comparatively stable gene expression and metabolite content across the season and tissue further suggested ‘H’ cultivar to be more tolerant to season and tissue. Higher cold tolerance has also been reported for chinary tea cultivar in a previous study^33^. An increased expression of FLS with decrease in TC content in old leaf tissue during development has indicated FLS as negative regulator of catechin biosynthesis in tea. Opposite relation in the expression of FLS and catechin biosynthesis has been reported in previous studies as well^31^,^34^. A significant higher TC in young tissue during development signifies the importance of young tissue (two leaf and a bud) in producing good quality tea. Together gene expression and content analysis across the season indicated that dormancy has reduced the quality of tea leaves by reducing the accumulation of major catechins. Moreover, higher TC during ‘A’ season emphasizes the importance of season in tea quality. Finding has suggested the key role of transcriptional regulation on catechins content during distinct stages of activity-dormancy cycle in different tissues and cultivars.

Caffeine is important bioactive ingredient synthesized in young leaves of *C. sinensis* plants. Previously, caffeine content and expression of TCS of caffeine biosynthesis pathway has been reported during development and seasonal variations in Kangra jat tea cultivar^4^. In present study, SAMS, MXMT, TCS genes were upregulated while XDH was downregulated with increase in caffeine content in young tissue and during ‘A’ season in all three cultivars. Results suggested key regulator role of SAMS, MXMT, TCS and XHD genes in caffeine biosynthesis/catabolism in leaves of different tea cultivars.

Transcriptome analysis has identified transcriptional regulation of ABA and GA levels during distinct stages of activity-dormancy cycle in three tea cultivars. ABA has been proposed to promote and maintain bud dormancy in woody plants^35^,^36^,^37^. It has been suggested that ABA levels are maintained by a delicate balance between its biosynthesis and catabolism, rather than simply by biosynthesis alone. High levels of ABA are maintained when both ABA biosynthesis and catabolism are active^26^. Further, ABA biosynthesis-related genes have been shown to upregulate whereas the genes responsible for ABA catalyses were downregulated during dormancy^12^,^15^. In this study, relative expression of both ABA biosynthesis (NCED and zeaxanthin epoxidase) and catabolism (ABA8′-hydroxylase) genes were found to be increased on the onset of dormancy. However, higher expression of NCED and zeaxanthin epoxidase than ABA8′-hydroxylase may describe the dormancy induced ABA accumulation. Conversely, lower expression of NCED and zeaxanthin during dormancy release (at MA season) further suggested reduced biosynthesis of ABA. Moreover, changes in hormonal levels could also change the hormone sensitivity in the pathway which could be another crucial manifestation in the activity-dormancy transitions of plant. Further, during dormancy higher expressions of ABA response genes as well as ABA biosynthetic genes was observed. Expression regulation has suggested the significant role of ABA in dormancy induction and maintenance in tea.

On the other hand, modulation of gibberellin (GA) biosynthesis and signaling could play a key role during the activity-dormancy cycle. Previous reports have shown an increase in GA levels during delayed dormancy in hybrid aspen^38^. Also, decrease in GA20ox expression and increase in GA2ox expression has been reported towards endodormancy release in Japanese pear. Upon dormancy release, higher expression of GA20ox and lower expression of GA2ox has been reported in hybrid aspen^12^. A significant decrease in GA levels has been reported in tea during dormancy^39^. Regulation of GA metabolism related genes in tea cultivars during dormancy and in subsequent dormancy release (MA season) has emphasized that this could be due to downregulation of GA20_OX_ expression and upregulation GA2_OX_ during dormancy and upregulation of GA20_OX_ and downregulation of GA2_OX_ during dormancy release. Hence, GA20_OX_ and GA2_OX_ are suggested to be the key targets for modulating GA levels in tea plant. DELLA protein has been reported to play an important role in maintenance of dormancy in leafy spurge^40^ and *paoenia ostii*^41^. Therefore, higher expression of DELLA protein during onset of dormancy has suggested its role in dormancy maintenance in different tea cultivars. Moreover, the gene expression data presented here, as well as hormonal measurements during dormancy in tea from earlier studies, indicate that ABA^42^ and GA^39^ metabolism display opposite responses during dormancy in tea.

Beside transcriptional regulation, epigenetic modifications such as chromatin remodeling, histone modification and DNA methylation have been known to play an important role in regulating gene expression in perennial plants during development and seasonal dormancy^14^. However, involvement of such epigenetic regulatory mechanisms is so far unknown in tea plant. There is growing evidence that genome-wide epigenetic regulation of gene expression is involved in dormancy regulation^14^,^43^. Higher DNA methylation and lower H4 acetylation levels were shown to induce dormancy in *Castanea sativa*^12^,^44^. Further, Bertoni *et al.*^45^ study has strengthen the importance of epigenetic regulation during dormancy by reporting histone deacetylases and histone lysine methyltransferase (SUVR3) upregulation in hybrid aspen. In poplar too, SWI2/SNF2-like genes responsible for chromatin modification have displayed upregulation during dormancy^46^, while its expression was reported to be significantly downregulated during dormancy release in leafy spurge^47^. In pursuance, the expression of chromatin remodeling components has been observed to be inducing the expression of different histone deacetylases, SUVR3 and SWI/SNF**-**like homolog during dormancy. The transcript abundance of histone deacetylases and SWI/SNF-like homolog during ‘D’ and ‘MD’ seasons and in old leaf tissue has suggested their role in facilitating the compaction of chromatin and suppression of gene expressions.

Apart from chromatin remodeling, *de novo* DNA methylation is also known to be involved in dormancy. DNA methylation can be precisely estimated by the transcript level of two genes such as DNA methyltransfereases and DNA glycosylases. Higher DNA methylation level was reported in *Castanea sativa* during bud rest period^41^ and in strawberry during induction of dormancy^48^. Expression of cytocine-5 methyltransferase too was reported higher in Japanese pears during endodormancy^15^. DEMETER-like and repressor of silencing (ROS) gene which encode DNA glycosylase are known to demethylate and activate genes contributing in dormancy release^12^. Moreover, Bertoni *et al.*^45^ reported that DEMETER-like DNA glycosylases are downregulated during dormancy. In agreement with these findings, higher expression of methyltransferase gene and lower expression of DEMETER-like as well as ROS genes observed during ‘D’ and ‘MD’ seasons in old tissue of three tea cultivars has suggested that higher DNA methylation could be one of the factors responsible for initiating dormancy in tea plant. Thus it appears that modulating expression of genes governing DNA methylation could represent a potent strategy for overcoming dormancy in tea.

**Figure 7:**
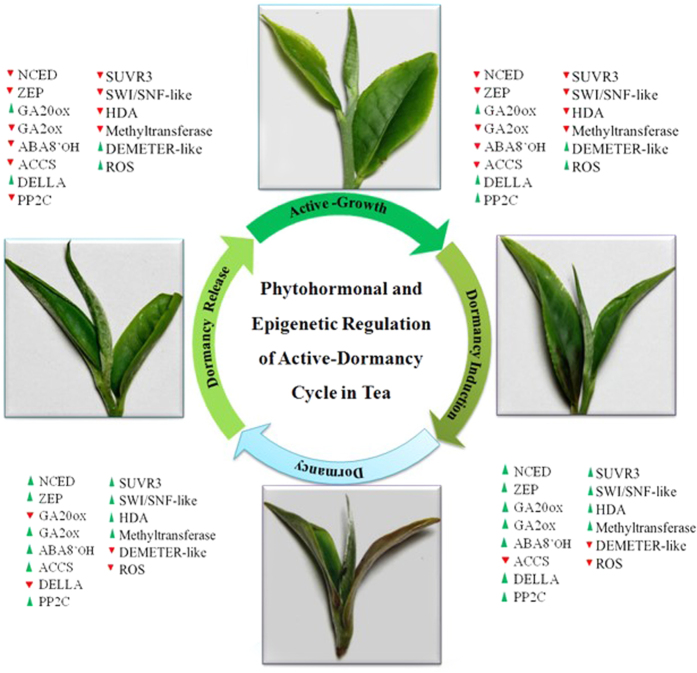
Importance of phytohormone metabolism and epigenetics related genes in transitions of seasonal active-dormancy cycling in *C. sinensis.* The expression of different phytohormone metabolism and epigenetics related genes are shown during complete seasonal active-dormancy cycle of tea. Genes were either up-regulated (green triangle) or down-regulated (red triangle).

Further, transcriptome analysis has revealed that expression of several genes related to phytohormones and histone/DNA modification was modulated during seasonal active-dormancy cycle in tea (Fig. 7). Thus, transcriptional control of phytohormones and histone/DNA modification are seem to be associated with transitions of active-dormancy cycle.

## Conclusion

Present study provides a global and a comparative survey of transcriptomes of three tea cultivars and thus may serve as an available genetic diversity resource for the tea plant. Study has generated gene expression profiles for two tissues at different seasons in tea plant. Data has also identified regulation of catechins and caffeine pathways in three tea cultivars during development and seasonal variation. Regulation of phytohormone metabolism and signaling at the transcriptional level was found to be important during development and seasonal variation in tea. Data has further revealed that along with transcriptional regulation, epigenetic control could play a key role in regulation of development and seasonal variation in three different tea cultivars.

## Experimental procedure

### Plant material and RNA isolation

Young tissue (two leaf and a bud) and old tissue (6^th^ position leaf from top bud) were collected from three different cultivars of tea (*Camellia sinensis* L.) viz. TV23 (Cambod), UPASI-9 (Assamica) and Him Sphurti (Chinary) during four different seasons viz. active (July), mid-dormant (October), dormant (December) and mid-active (April) from the experimental tea farm of the CSIR-Institute of Himalayan Bioresource Technology (32°6′ N latitude; 76°33′ E longitude; 1289 m above mean sea level)^5^.Total twenty four samples namely TBA, TSA, HBA, HSA, UBA, USA, TBMD, TSMD, HBMD, HSMD, UBMD, USMD, TBD, TSD, HBD, HSD, UBD, USD, TBMA, TSMA, HBMA, HSMA, UBMA, USMA were taken for the present study. ‘T’, ‘H’ and ‘U’ stand for TV23, Him Sphurti and UPASI-9 cultivars respectively. Bud sample tissue is denoted by ‘B’ while 6^th^ position leaf is denoted by ‘S’. A, MD, D, MA represents four different seasons active, mid-dormant, dormant and mid-active respectively (Fig. 1). Total RNA was isolated from three biological replicates of each tissue using RNeasy plant mini kit (Qiagen, Germany). Further, the quality and quantity of each RNA sample was determined using bioanalyzer (Agilent technologies, USA) and NanoDrop (Thermo Scientific).

### cDNA library preparation and illumina sequencing

Total 5 μg high quality RNA (pool of three biological replicates for individual tissue) was used for preparing cDNA library (Illumina TruSeq RNA sample preparation kit v2, Illumina Inc., USA). Quantification and the validation of insert size was done on Qubit fluorometer using Qubit dsDNA BR assay kit (Life Technologies, USA) and Bioanalyzer respectively. The clusters were generated for prepared libraries and sequenced according to manufacturer’s protocol employing TruSeq PE Cluster Kit v5-CS-GA; Illumina Inc; USA as well as Illumina GAIIx platform. The fluorescent images thus obtained were further processed to sequence and base-calling as well as quality value calculations were performed using Illumina data processing pipeline (RTA version 1.9).

### *De novo* assembly and homology search

Pair end (PE) sequence reads were generated with 72 bases length using CASAVA 1.8 package tool provided by Illumina. Reads’ quality was assessed using FilteR tool^28^. High quality reads were used for *de novo* assembly till scaffold level using SOAPdenovo-trans tool for three tea cultivars separately^49^. *K*-mer size of 61 was selected for each of the assembly as it achieved the best balance between the numbers of transcripts produced, average length of transcripts, N50 length value and average coverage of total assembly. For more effective assembly, gap filling was carried out by GapCloser to complete scaffolds using the average insert length of 300 bases with paired-end information^50^. Sequence redundancy was removed employing CD-HIT-EST (similarity cut-off ≥90%)^51^ and TGICL-CAP3 (identity ≥90%) tools^52^. Further, dissimilar sequence clustering (DS) was performed using BLASTX hits^28^. A transcript sequence with highest bit score and longest sequence length was selected as unigene representative from each DS cluster.

### Annotation and differential expression analysis

Annotations for Gene Ontology (GO), Kyoto Encyclopedia of Genes and Genomes (KEGG) and Enzyme Commission Codes (EC) for assembled transcripts were carried out using Annot8r annotation tool against UniProt database^53^. Based on highest bit score and E-value, top-hits were selected. For expression measurement as FPKM (Fragments per kilobase of transcript per million mapped) by RSEM^54^, reads from all the conditions were mapped back to assembled transcripts using Bowtie tool for each cultivar individually^55^.

For differential expression (DE) calculation, mapped read count for each tea transcript, according to each conditions were used. DE was carried out using EdgeR package in R^56^. EdgeR estimates the mean and variance of raw read counts under a negative binomial distribution and use exact test to identify differentially expressed transcripts. MA plot was also plotted for DE during various conditions. MA-plot is a scatter plot of logarithmic fold changes (on the y-axis) versus the mean of normalized counts (on the x-axis).

AgriGO tool^57^ based singular enrichment analysis was carried out to identify the enriched Gene Ontology terms in all comparative conditions with DE genes at significance level of 0.05. To counterbalance the problem of multiple comparisons, hyper-geometric statistical test was applied with Bonferroni correction method. Similarly, KEGG pathways based enrichment analysis was also performed as described earlier^58^. Where, hyper-geometric test was applied with adjust P-values for multiple comparisons with BH method^59^ in R package^60^. Further, differentially enriched pathways with p-value ≤ 0.05 and correction value/FDR (False discovery rate) ≤ 0.05 were selected as significant.

### Quantitative real-time polymerase chain reaction (qRT-PCR) analysis

For the validation of reliability of RNA-Seq data, the relative expression levels of 10 selected genes were measured using qRT-PCR. First strand cDNA was synthesized from1μg of DNaseI- treated total RNA using high capacity cDNA reverse transcription kit (Applied Biosystems, USA). Primer express 3.0 software (Applied Biosystems, USA) was used to design gene specific primers for qRT-PCR (Supplementary Table S10). Glyceraldehyde-3-phosphate dehydrogenase (GAPDH) from tea was used as reference gene. Further, qRT amplification was performed in triplicate on a Step One real-time PCR machine (Applied Biosystems, USA) using SYBR Green qPCR Master Mix (Thermo Scientific, USA). The conditions for qRT-PCR were 94 °C for 4 min followed by 40 cycles of 94 °C for 30 s, 58–61 °C for 30 s and 72 °C for 30 s (data collection) with a final melting curve analysis. The relative expression of each gene was calculated using Ct method, a comparative 2^ddCT method^61^.

### Quantification of catechins and caffeine

Extraction and quantification of six tea catechins namely catechin (C), epi-catechin (EC), gallocatechin (GC), epi-gallocatechin (EGC), epi-catechin gallate (ECG), epigallocatechingallate (EGCG) and caffeine was carried out using the method of Joshi *et al.*^62^. Identification and quantification of C, GC, EC, ECG, EGC, EGCG and caffeine was performed by matching retention time, co-injections and spectral matching with authentic standards. Mean area of three replicate injections was considered for finding the concentrations in samples.

### Statistical analysis of catechins and caffeine content

Different catechins and caffeine content was expressed as the mean ± standard deviation (SD) of three technical replicates. The data were analyzed by two-way ANOVA followed by Duncan’s multiple range test at P ≤ 0.05. Further, statistical significance of differences between samples was assessed via t-test and f-test. The correlation was analyzed via Pearson correlation at P ≤ 0.05.

## Additional Information

**How to cite this article**: Kumar, A. *et al.* Comparative Transcriptome Analysis of Chinary, Assamica and Cambod tea (*Camellia sinensis*) Types during Development and Seasonal Variation using RNA-seq Technology. *Sci. Rep.* **6**, 37244; doi: 10.1038/srep37244 (2016).

**Publisher’s note**: Springer Nature remains neutral with regard to jurisdictional claims in published maps and institutional affiliations.

## Supplementary Material

Supplementary Information

Supplementary Table S1

Supplementary Table S2

Supplementary Table S3

Supplementary Table S4

Supplementary Table S5

Supplementary Table S6

Supplementary Table S7

Supplementary Table S8

Supplementary Table S9

Supplementary Table S10

## Figures and Tables

**Table 1:** Details of various samples taken for study and read sequence information.

Sample description	Total PE read pairs before quality filtering	Total PE read pairs after quality filtering	%Quality reads
Him Sphurti Bud Active season (HBA)	8383013	6811932	81.259
Him Sphurti Bud Dormant season (HBD)	5844616	4210713	72.044
Him Sphurti Bud Mid-active season (HBMA)	5478981	4308535	78.638
Him Sphurti Bud Mid-dormant season (HBMD)	2810901	2268152	80.691
Him Sphurti 6^th^ leaf Active season (HSA)	5873325	4728513	80.508
Him Sphurti 6^th^ leaf Dormant season (HSD)	4080183	2917224	71.497
Him Sphurti 6^th^ leaf Mid-active season (HSMA)	8600606	6689386	77.778
Him Sphurti 6^th^ leaf Mid-dormant season (HSMD)	6302186	5089903	80.764
Total	47373811	37024358	78.154
TV23 Bud Active season (TBA)	7843963	6377985	81.311
TV23 Bud Dormant season (TBD)	4471405	3209323	71.774
TV23 Bud Mid-active season (TBMA)	6378741	5098306	79.927
TV23 Bud Mid-dormant season (TBMD)	5069530	4024902	79.394
TV23 6^th^ leaf Active season (TSA)	5738215	4440860	77.391
TV23 6^th^ leaf Dormant season (TSD)	7440203	5324285	71.561
TV23 6^th^ leaf Mid-active season (TSMA)	7754754	6148422	79.286
TV23 6^th^ leaf Mid-dormant season (TSMD)	5683546	4580869	80.599
Total	50380357	39204952	77.818
UPASI-9 Bud Active season (UBA)	6229751	5074566	81.457
UPASI-9 Bud Dormant season (UBD)	2239045	1611499	71.973
UPASI-9 Bud Mid-active season (UBMA)	6712883	5356217	79.790
UPASI-9 Bud Mid-dormant season (UBMD)	4161823	3369880	80.971
UPASI-9 6^th^ leaf Active season (USA)	8281493	6739057	81.375
UPASI-9 6^th^ leaf Dormant season (USD)	2394138	1684328	70.352
UPASI-9 6^th^ leaf Mid-active season (USMA)	5220964	4142548	79.345
UPASI-9 6^th^ leaf Mid-dormant season (USMD)	2942491	2384964	81.053
Total	38182588	30363059	79.521

**Table 2:** Profile of different catechins and caffeine concentration in three tea cultivar during development and seasonal variation.

Sample	GC mg/g	CV%	C mg/g	CV%	EC mg/g	CV%	ECG mg/g	CV%	EGC mg/g	CV%	EGCG mg/g	CV%	TC mg/g	CV%	Caffeine mg/g	CV%
**HBA**	8.06 ± 0.14^d^	1.737	14.03 ± 0.03^g^	0.214	19.94 ± 0.01^a^	0.050	30.57 ± 0.15^e^	0.491	18.19 ± 0.11^dc^	0.605	107.48 ± 0.07^c^	0.065	198.27 ± 0.02^c^	0.008	44.40 ± 0.68^b^	1.533
**HSA**	6.57 ± 0.34^g^	5.175	4.22 ± 0.31^nop^	7.346	12.27 ± 0.08^e^	0.652	14.94 ± 1.65^k^	11.044	11.68 ± 0.36^g^	3.039	18.76 ± 0.12^on^	0.640	68.42 ± 1.75^jk^	2.558	19.65 ± 0.37^l^	1.895
**HBMD**	7.49 ± 0.05^e^	0.668	12.09 ± 0.61^h^	5.045	13.38 ± 0.12^d^	0.897	20.15 ± 0.22^h^	1.092	17.88 ± 0.25^dc^	1.370	97.66 ± 2.99^fe^	3.062	168.62 ± 3^e^	1.779	33.18 ± 0.26^e^	0.786
**HSMD**	0	0.000	4.68 ± 0.44^mn^	9.402	11.63 ± 0.05^f^	0.430	8.45 ± 0.0.4^m^	4.734	10.21 ± 0.03^ih^	0.294	15.55 ± 0.04^srq^	0.257	50.51 ± 0.79^ml^	1.554	18.39 ± 0.9^n^	4.824
**HBD**	8.69 ± 0.32^c^	3.682	6.39 ± 0.02^j^	0.313	10.24 ± 0.04^ji^	0.391	18.78 ± 0.51^i^	2.716	16.03 ± 0.48^e^	2.994	53.83 ± 0.49^i^	0.910	113.97 ± 1.85^h^	1.623	29.25 ± 0.20^g^	0.684
**HSD**	0	0.000	4.03 ± 0.03^op^	0.744	9.85 ± 0.01^h^	0.102	7.29 ± 0.16^on^	2.195	10.25 ± 0.03^ih^	0.244	16.93 ± 0.01^qp^	0.059	48.34 ± 0.1^nm^	0.197	16.51 ± 0.05^o^	0.311
**HBMA**	0	0.000	15.59 ± 0.07^e^	0.449	15.82 ± 0.02^b^	0.126	24.43 ± 0.5^g^	2.047	14.96 ± 0.26^f^	1.638	98.67 ± 0.04^e^	0.041	169.45 ± 0.14^e^	0.083	27.60 ± 0.10^h^	0.397
**HSMA**	0	0.000	4.5 ± 0.39^no^	8.667	10.38 ± 0.04^i^	0.385	3.21 ± 0.01^r^	0.312	10.24 ± 0.02^ih^	0.146	16.75 ± 0.01^rpq^	0.060	45.07 ± 0.45^o^	0.998	18.78 ± 0.05^nm^	0.282
**TBA**	7.34 ± 0.28^e^	3.815	32.68 ± 0.67^a^	2.050	13.31 ± 0.17^d^	1.277	57.22 ± 0.17^a^	0.297	24.91 ± 0.37^a^	1.485	103.87 ± 1.37^d^	1.319	239.31 ± 0.1^b^	0.038	36.52 ± 0.15^c^	0.397
**TSA**	0	0.000	5.28 ± 0.11^kl^	2.083	9.95 ± 0.05^h^	0.503	9.11 ± 0.01^m^	0.110	9.38 ± 0.14^k^	1.439	17.77 ± 0.1^po^	0.563	51.48 ± 0.17^l^	0.330	18.68 ± 0.08^nm^	0.446
**TBMD**	6.88 ± 0.06^f^	0.872	26.29 ± 0.64^d^	2.434	10.12 ± 0.18^j^	1.779	26.57 ± 0.44^f^	1.656	18.35 ± 0.05^c^	0.272	75.97 ± 0.7^h^	0.921	164.16 ± 2.05 ^f^	1.249	23.14 ± 0.05^i^	0.222
**TSMD**	0	0.000	8.17 ± 0.04^i^	0.490	8.07 ± 0.05^m^	0.620	4.21 ± 0.01^q^	0.238	6.27 ± 0.43^m^	6.778	15.12 ± 0.02^sr^	0.132	41.83 ± 0.44^l^	1.052	16.12 ± 0.03^o^	0.156
**TBD**	10.17 ± 0.15^a^	1.475	6.77 ± 0.03^j^	0.443	5.68 ± 0.25^p^	4.401	11.36 ± 0.62^l^	5.458	18.08 ± 0.25^dc^	1.383	37.95 ± 2.83^k^	7.457	89.99 ± 1.54^i^	1.711	21.39 ± 0.19^j^	0.880
**TSD**	5.84 ± 0.1^h^	1.712	5.04 ± 0.04^lm^	0.794	4.37 ± 0.01^q^	0.229	10.86 ± 0.08^l^	0.737	12.04 ± 0.26^g^	2.159	31.62 ± 0.12^l^	0.380	69.76 ± 0.44^j^	0.631	10.0 ± 0.54^q^	5.365
**TBMA**	7 ± 0.07^f^	1.000	30.44 ± 0.02^b^	0.066	9.4 ± 0.03^k^	0.319	33.97 ± 0.17^d^	0.500	23.44 ± 0.21^b^	0.896	92.87 ± 0.83^g^	0.894	197.11 ± 1.18^dc^	0.599	31.14 ± 0.49^f^	1.574
**TSMA**	0	0.000	3.96 ± 0.03^p^	0.758	8.73 ± 0.06^l^	0.687	5.43 ± 0.06^p^	1.105	8.75 ± 0.37^l^	4.171	13.28 ± 0.01^t^	0.075	40.14 ± 0.45^p^	1.121	23.40 ± 0.46^i^	1.958
**UBA**	8.75 ± 0.26^c^	2.971	27.19 ± 0.01^c^	0.037	15.42 ± 0.08^c^	0.519	39.35 ± 0.14^c^	0.356	23.31 ± 0.11^b^	0.450	154.61 ± 1.18^a^	0.763	268.61 ± 1.37^a^	0.510	34.58 ± 0.11^d^	0.325
**USA**	6.03 ± 0.14^h^	2.322	4.2 ± 0.02^nop^	0.476	10.76 ± 0.01^h^	0.093	16.45 ± 0.23^j^	1.398	10.03 ± 0.33^ji^	3.290	19.99 ± 0.06^nm^	0.300	67.46 ± 0.13^k^	0.185	19.09 ± 0.04^ml^	0.218
**UBMD**	0	0.000	6.71 ± 0.07^j^	1.043	9.52 ± 0.01^k^	0.105	14.55 ± 0.08^k^	0.550	11.73 ± 0.29^g^	2.430	96.03 ± 0.01^f^	0.010	138.53 ± 0.1^g^	0.043	27.24 ± 0.61^h^	2.231
**USMD**	0	0.000	5.6 ± 0.07^k^	1.250	7.98 ± 0.0^2m^	0.251	7.61 ± 0.28^n^	3.679	10.52 ± 1.02^ih^	9.696	18.26 ± 0.59^po^	3.231	49.96 ± 0.21^nml^	0.420	23.13 ± 0.75^i^	3.231
**UBD**	9.23 ± 0.2^b^	2.167	4.56 ± 0.06^n^	1.316	8.09 ± 0.02^m^	0.247	11.64 ± 0.44^l^	3.780	10.7 ± 0.05^h^	0.467	45.69 ± 0.31^j^	0.678	89.9 ± 1.07^i^	1.190	20.63 ± 0.26^k^	1.263
**USD**	0	0.000	3.74 ± 0.01^p^	0.267	6.9 ± 0.06^o^	0.870	5.51 ± 0.24^p^	4.356	8.74 ± 0.4^l^	4.577	14.76 ± 0.23^ts^	1.558	39.64 ± 0.13^p^	0.315	7.48 ± 0.02^p^	0.034
**UBMA**	0	0.000	14.98 ± 0.33 ^f^	2.203	10.94 ± 0.08^g^	0.731	37.04 ± 1.2^d^	3.240	17.66 ± 0.53^d^	2.973	114.62 ± 1.58^b^	1.378	195.23 ± 3.71^d^	1.900	32.63 ± 0.21^e^	0.644
**USMA**	0	0.000	3.84 ± 0.15^p^	3.906	7.09 ± 0.03^n^	0.423	6.49 ± 0.04^o^	0.616	9.49 ± 0.07^kj^	0.685	20.96 ± 0.01 ^m^	0.048	47.86 ± 0.15^n^	0.313	15.45 ± 0.01^p^	0.065

The values are mean ± SD; Mean values having different superscript lowercase letters are significantly different according to Duncan’s multiple range test at P ≤ 0.05; SEM 3.01.

**Table 3:** Correlation between expression of catechins and caffeine biosynthesis-related genes and total catechin and caffeine content among three tea cultivars during development and seasonal variation.

TC					Caffeine				
Gene	A	MD	D	MA	Gene	A	MD	D	MA
**PAL**	0.903*	0.619	0.758*	0.829*	**IMPDH**	0.831*	−0.786*	−0.427	0.556
**C4H**	0.922*	0.845*	0.606	0.735	**AMPD**	0.725*	0.741	−0.584	0.959*
**4CL**	0.791*	0.829*	0.672	0.661	**SAMS**	0.816*	0.829*	0.528	0.962*
**CHS**	0.796*	0.872*	0.668	0.682	**MXMT**	0.323	−0.009	0.259	0.649
**CHI**	0.845*	0.734	0.402	0.767*	**TCS**	0.524	0.459	0.431	0.840*
**F3′H**	0.914*	0.337	0.632	0.805*	**XDH**	−0.911*	−0.788*	−0.715	−0.733
**F3′5′H**	0.881*	0.641	0.258	0.691	**5′NT**	0.873*	0.804*	0.007	0.309
**FNS**	0.268	0.832*	0.746*	−0.524					
**F3H**	0.592	0.666	0.402	0.625					
**DFR**	0.980**	0.805*	0.431	0.864*					
**LAR**	0.978**	0.787*	0.416	0.958**					
**ANS**	0.957**	0.833*	−0.206	0.965**					
**ANR**	0.361	0.603	0.333	0.391					
**FLS**	0.352	0.112	0.003	−0.450					

Asterisks indicate that the genes from catechin and caffeine pathways are significantly correlated with the catechin and caffeine content (*P ≤ 0.05 and 0.01, **P ≤ 0.001).

**Table 4:** Effect of cultivar, season, tissue, cultivar*season, cultivar*tissue, season*tissue and cultivar*season*tissue on the accumulation of different catechins and caffeine in tea plants.

Effect	DF	GC			C			EC			ECG			
		SS	MS	F-value	SS	MS	F-value	SS	MS	F-value	SS	MS	F-value	p-value
cultivar	2	32.778	16.389	824.52	641.422	320.711	4185.3	239.376	119.688	15930	184.32	92.16	372.81	0.0005*
season	3	319.342	106.447	5355.28	882.081	294.027	3837.1	335.952	111.984	14904	3031.39	1010.46	4087.69	0.0005*
tissue	1	380.190	380.190	19127.07	2465.424	2465.424	32174.3	143.439	143.439	19091	6388.74	6388.74	25844.74	0.0005*
cultivar*season	6	172.128	28.688	1443.27	306.135	51.023	665.9	16.175	2.696	359	465.52	77.59	313.87	0.0005*
cultivar*tissue	2	34.923	17.462	878.48	412.982	206.491	2694.8	11.615	5.807	773	335.65	167.82	678.91	0.0005*
season*tissue	3	61.425	20.475	1030.07	898.680	299.560	3909.3	47.615	15.872	2112	1593.97	531.32	2149.39	0.0005*
cultivar*season*tissue	6	116.131	19.355	973.74	298.360	49.727	648.9	21.722	3.620	482	885.88	147.65	597.28	0.0005*
Error	48	0.954	0.020		3.678	0.077		0.361	0.008		11.87	0.25		
Total	71	1117.871			5908.763			816.253			12897.33			
		**EGC**			**EGCG**			**TC**			**Caffeine**			
**Effect**	**DF**	**SS**	**MS**	**F-value**	**SS**	**MS**	**F-value**	**SS**	**MS**	**F-value**	**SS**	**MS**	**F-value**	**p-value**
cultivar	2	69.29	34.65	294.9	1775.6	887.8	884.3	271.9	135.9	78.1	188.48	94.24	670.4	0.0005*
season	3	164.09	54.70	465.5	13003.1	4334.4	4317.3	50648.5	16882.8	9704.1	1177.47	392.49	2792.0	0.0005*
tissue	1	1191.94	1191.94	10144.5	92344.7	92344.7	91981.9	249465.2	249465.2	143389.8	3003.55	3003.55	21366.1	0.0005*
cultivar*season	6	99.70	16.62	141.4	2095.8	349.3	347.9	5838.6	973.1	559.3	363.86	60.64	431.4	0.0005*
cultivar*tissue	2	140.51	70.26	597.9	2038.7	1019.3	1015.3	609.6	304.8	175.2	56.68	28.34	201.6	0.0005*
season*tissue	3	127.02	42.34	360.4	15199.9	5066.6	5046.7	40420.3	13473.4	7744.4	282.62	94.21	670.2	0.0005*
cultivar*season*tissue	6	108.05	18.01	153.3	1532.2	255.4	254.4	7159.1	1193.2	685.8	187.05	31.18	221.8	0.0005*
Error	48	5.64	0.12		48.2	1.0		83.5	1.7		6.75	0.14		
Total	71	1906.25			128038.1			354496.6			5266.47			

DF = degree of freedom, SS = Sum of squares, MS = Mean square. Asterisks indicate that the content was significantly different (*P ≤ 0.05).
