# Supplementary material for: Comparative Transcriptome Analysis of Chinary, Assamica and Cambod tea (Camellia sinensis) Types during Development and Seasonal Variation using RNA-seq Technology
Source: Sci Rep. 2016 Nov 17;6:37244. doi: 10.1038/srep37244 (PMC5112563; doi:10.1038/srep37244)
Supplement: Supplementary Information [file srep37244-s1.doc]

SUPPORTING INFORMATION

**Comparative Transcriptome Analysis of Chinary, Assamica and Cambod tea** (***Camellia sinensis*) Types during Development and Seasonal Variation using RNA-seq Technology**

Ajay Kumar^1,3^,†, Vandna Chawla^2,4^,†, Eshita Sharma^5^, Pallavi Mahajan^2^, Ravi Shankar^2,3,*^, Sudesh Kumar Yadav^1,3,6,*^

^1^Plant Metabolic Engineering Laboratory, Biotechnology Division, CSIR-Institute of Himalayan Bioresource Technology, Palampur-176061 (HP)

^2^Studio of Computational Biology & Bioinformatics, Biotechnology Division, CSIR-Institute of Himalayan Bioresource Technology, Palampur-176061 (HP)

^3^Academy of Scientific and Innovative Research, New Delhi, India

^4^Department of Biotechnology, Guru Nanak Dev University, Amritsar, Punjab

**^5^Food and Nutraceutical Division,** CSIR-Institute of Himalayan Bioresource Technology, Palampur-176061 (HP), India.

^6^Center of Innovative and Applied Bioprocessing (CIAB), Mohali-160071, Punjab, India

†Contributed equally

*Corresponding authors

E-mail: [**ravish@ihbt.res.in**](mailto:ravish@ihbt.res.in); [**ravish9@gmail.com**](mailto:ravish9@gmail.com)

[**sudesh@ciab.res.in**](mailto:sudesh@ciab.res.in); skyt@rediffmail.com


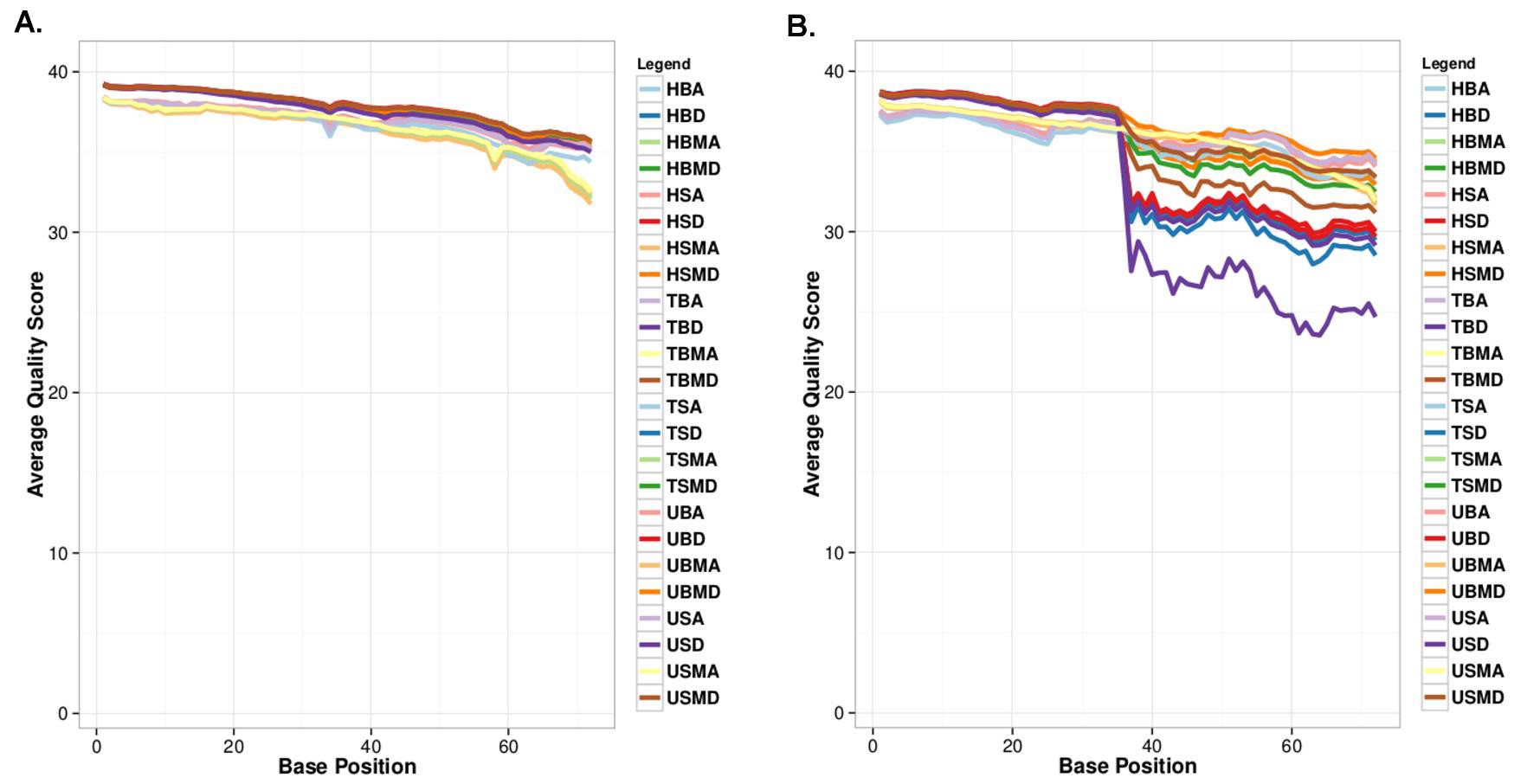


**Supplementary figure 1.** Plots showing read quality-score for different tea samples in PE data. The line plot represents average quality score along Y-axis and read length per base position on X-axis.


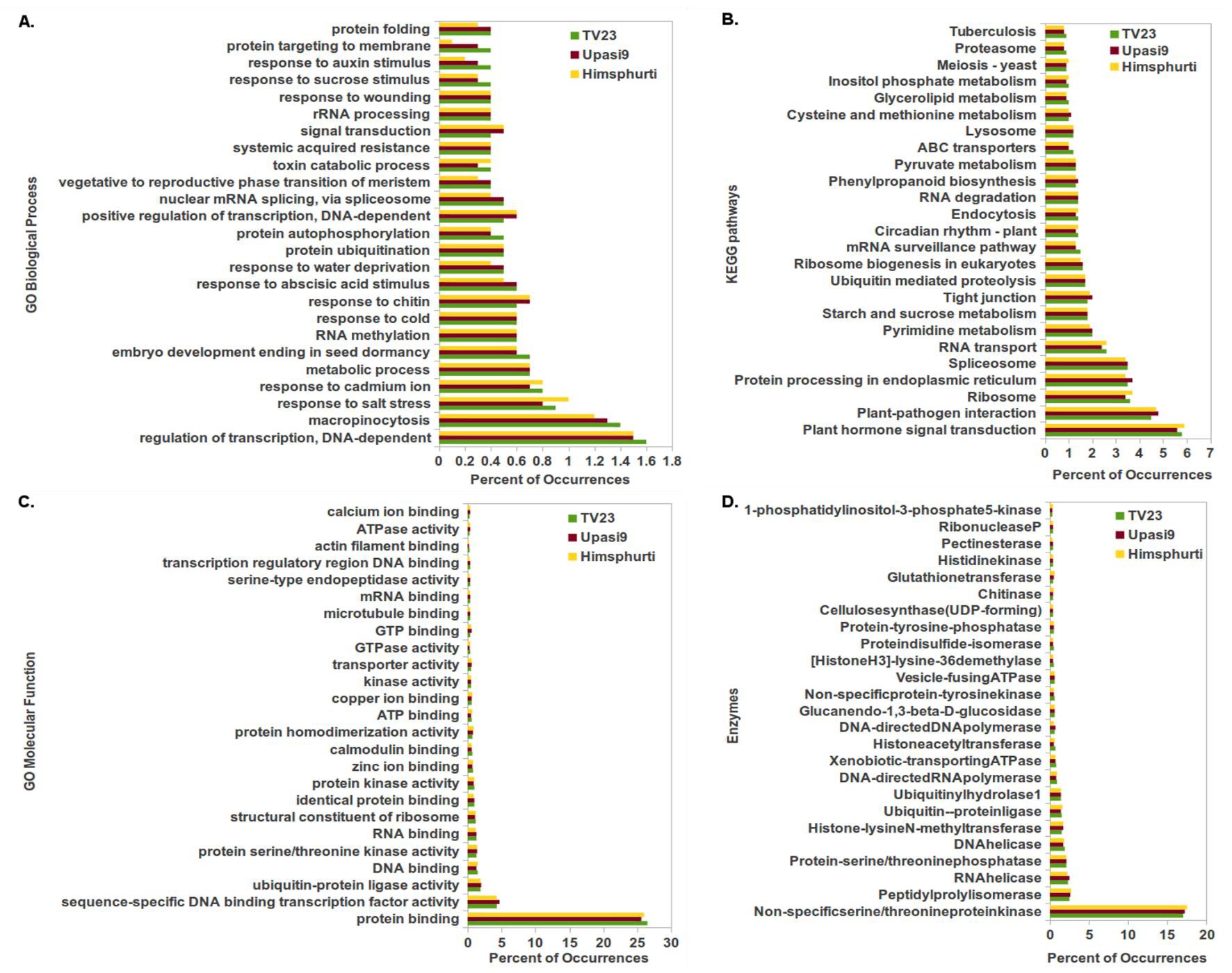


**Supplementary figure 2.** Relative abundance of different biological processes (A), KEGG pathways (B), molecular functions (C), and enzyme classes (D) among three tea cultivars with corresponding percentage.

A.

- 1. HBA vs HSA b.HBDvsHSD c.HBMD vs HSMD d.HBMA vs HSMA

I.

*';!*

,..

*';!*

0 ...· e e

/


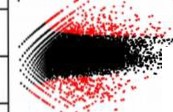
....·. Ill ../· -:

Ill Ill

Ill

(.) (.) 0 (.) (.)

u. 0 0 *:* ..

u. u. u.

0

I I I - .

I ., ·

Ill Ill Ill Ill

.

................ . .

0 0 I

0 '-.

I ·-.

0

I

10 8 10 12

-.............._

·- . I

''

logCoun1s logCounts

| 6 | 10 | 12 | 14 | 0 | 10 | 15 |
| --- | --- | --- | --- | --- | --- | --- |
| logCounts |  |  |  |  | logCounts |  |

II.

*';!*

a. TBA vs TSA b.TBD vs TSD c. TBMD vs TSMD d.TBMA vs TSMA


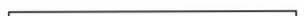

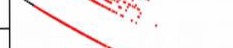

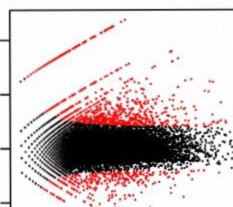


e .....··

Ill ./·

Ill Ill

(.) (.) (.) (.)

Ill

u. u. 0 u. u. ...

0 0

0

Ill Ill

I I Ill

0 ··..·

I

Ill

I

I 0

·-

Ill I 0

I I

•..........

Ill.

0 10 15 10 12 10 15 0 8 10 12

togCounts logCounts logCounts logCounts

1. UBAvs USA b. UBDvs USD c.UBMD vs USMD d.UBMA vs USMA


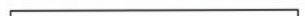

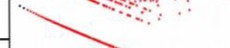

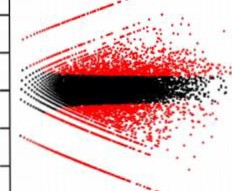


*';!*

-· *';! ';!*

Ill


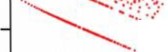


,......."' ..-

Ill Ill Ill

(.) (.) 0 (.) (.)

u. 0 u. u. u. 0

*9*"' 0

Ill

I

Ill

I

0

................... ..

Ill Ill

I I

Ill

I

-..

I - -

0

I Ill

I

10 12 14 10 12 10 12 10 12 14

togCounts logCounts logCounts logCounts

B.

1. a.HBAvs HBD


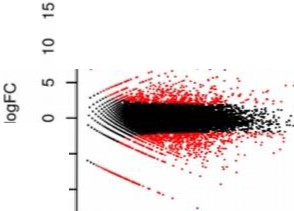


1. HBA vs HBMD
2. HBA vs HBMA

__.........,--

0

I

.- ·.
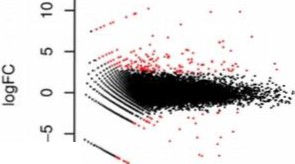


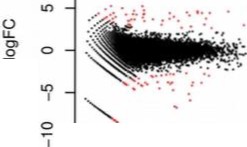


0

I

0 2 6 8 10 12 14

logCounts

1. HBMA vs HBD

0 2 4 6 8 10 12 14

1. HBMA vs HBMD

0 5 10 15

logCounts

1. HBMD vs HBD


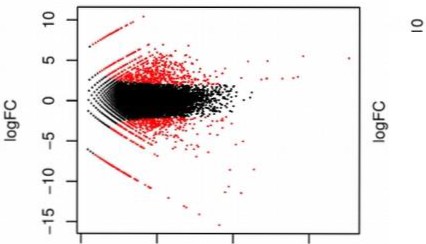

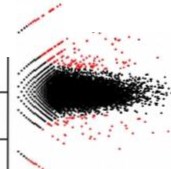
.../


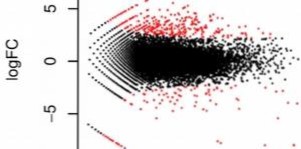
"'

0

"'

0 5 10 15

logCounts

II. a.TBAvs TBD

0

I

5 10

logCounts

- 1. TBA vs TBMD

0

I

15 2 4 6 8 10 12 14

logCounts

- 1. TBA vs TBMA

0


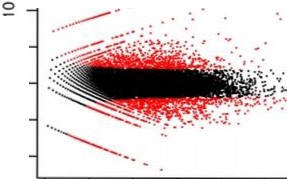


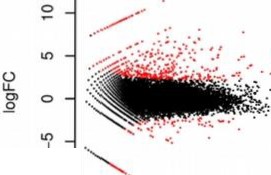
.···

"' *) _,.*

0

0 ·- ··:.. ···.; · ·· ...

'? ··. .

0 0

I I

0 2 4 6 8 10 12

logCounts

- 1. TBMA vs TBD

0 10

logCounts

- 1. TBMA vs TBMD

15 0 2 6 8 10 12

nts

- 1. TBMD vs TBD

"'


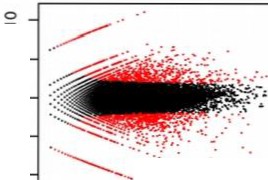
0 0

'6- '?

I"'

"'··-.

,...,·-'"

../· ..


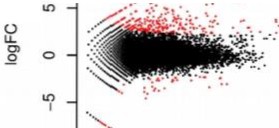


0

I

-.,

I -----,-------.------


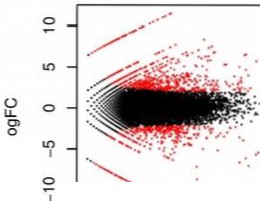


2 6 8 10 12

logCounts


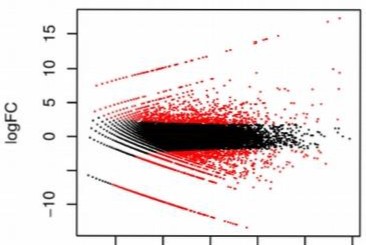
Ill. a.UBA vs UBD

fr

0

"'

0

"' ··

5 10

logCounts

1. UBA vs UBMD


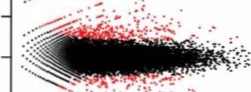
_,_....

-.....


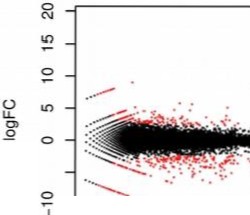


15 5 10 15

logCounts

1. UBA vs UBMA

........

0

I

2 4 6 8 10 12

logCounts

1. UBMA vs UBD

,-----------------

2 6 8 10 12

logCounts

1. UBMA vs UBMD


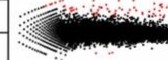

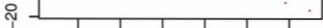


....-·-·

"'

0

..,

0

I

0 2 4 6 8 10 12

logCounts

1. UBMD vs UBD


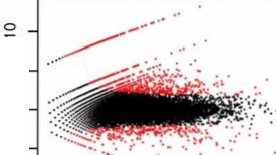


"'

0

0

'?


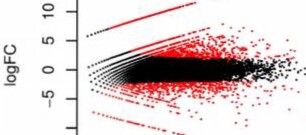
0 -........

I"' I

2 4 6 8 10 12

logCounts

2 6 8 10 12

logCounts

2 4 6 8

logCounts

10 12


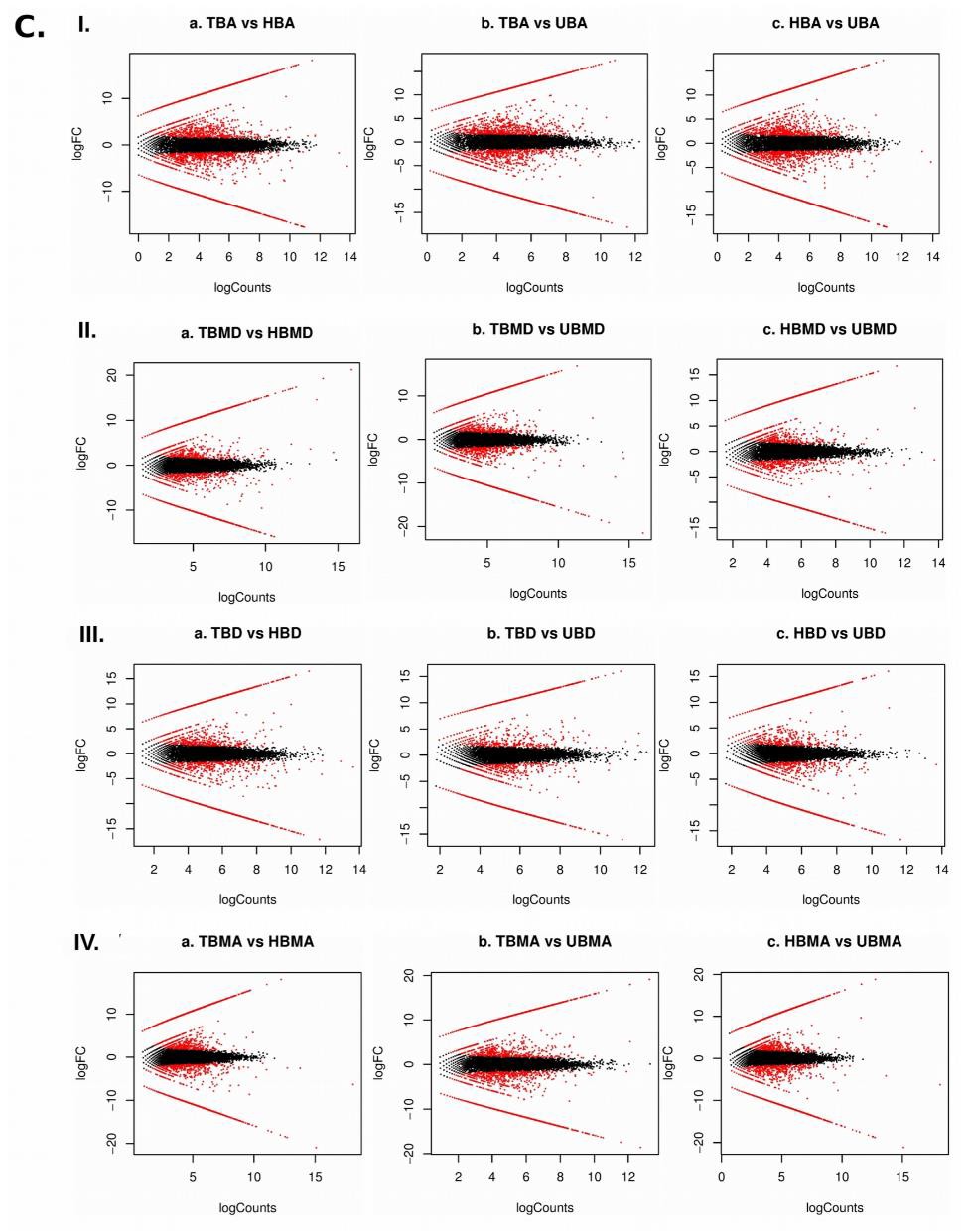


**Supplementary figure 3.** MA-plots representing differentially expressed genes (DEGs) during development (A), seasons (B) and cultivars (C) comparisons. Plot shows logarithmic fold changes on the y-axis against the logarithmic mean of counts on the x-axis. The transcripts with similar expression levels in comparative conditions appearing around the horizontal line (y=0) and represented in black color, while the transcripts showing significant difference

between comparative conditions (p-value <=0.05) were represented in red color.

1. Development Comparisons
2. Molecular Function (UP-regulated)
3. HBA vs HSA


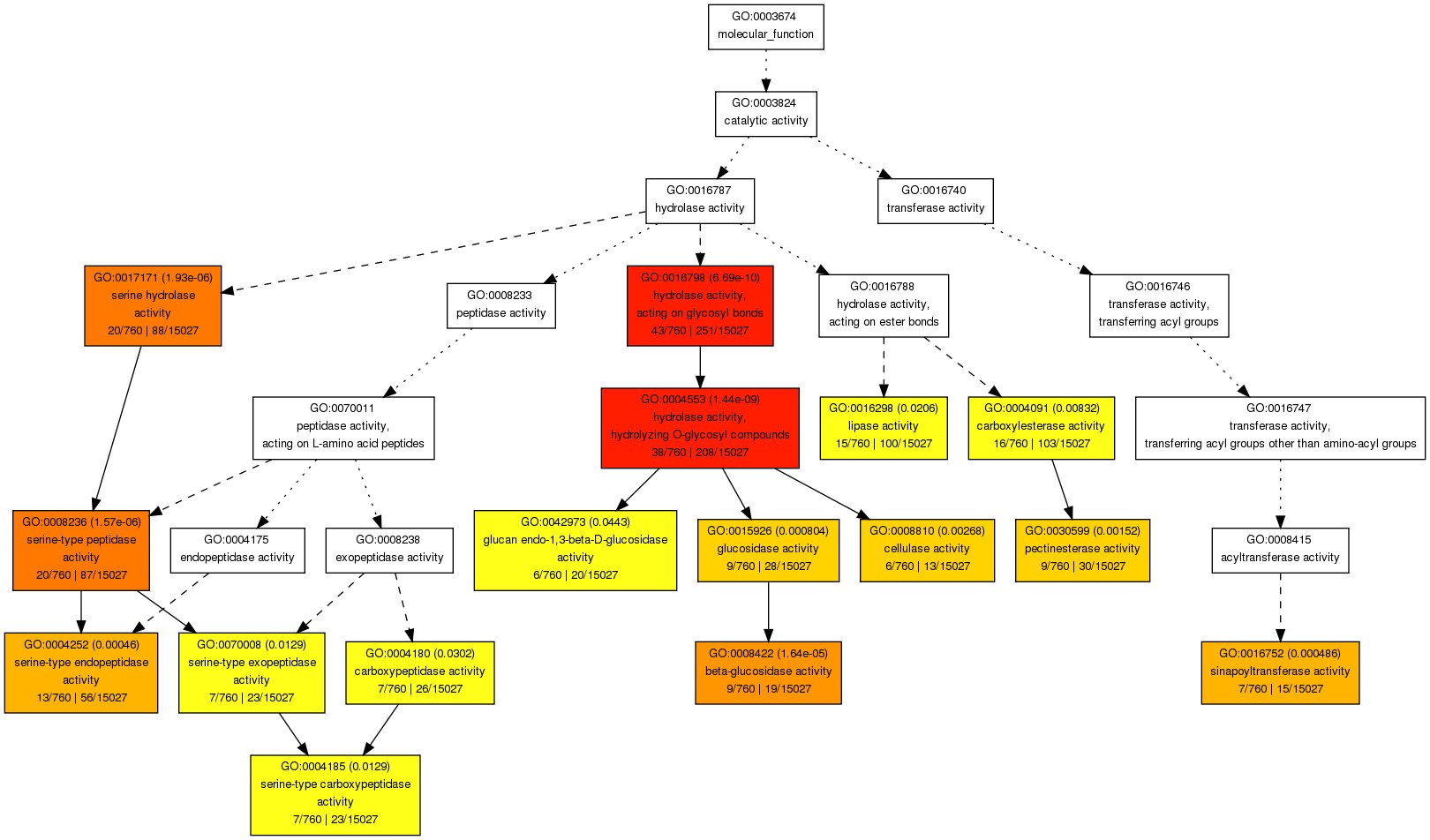


1. HBD vs HSD


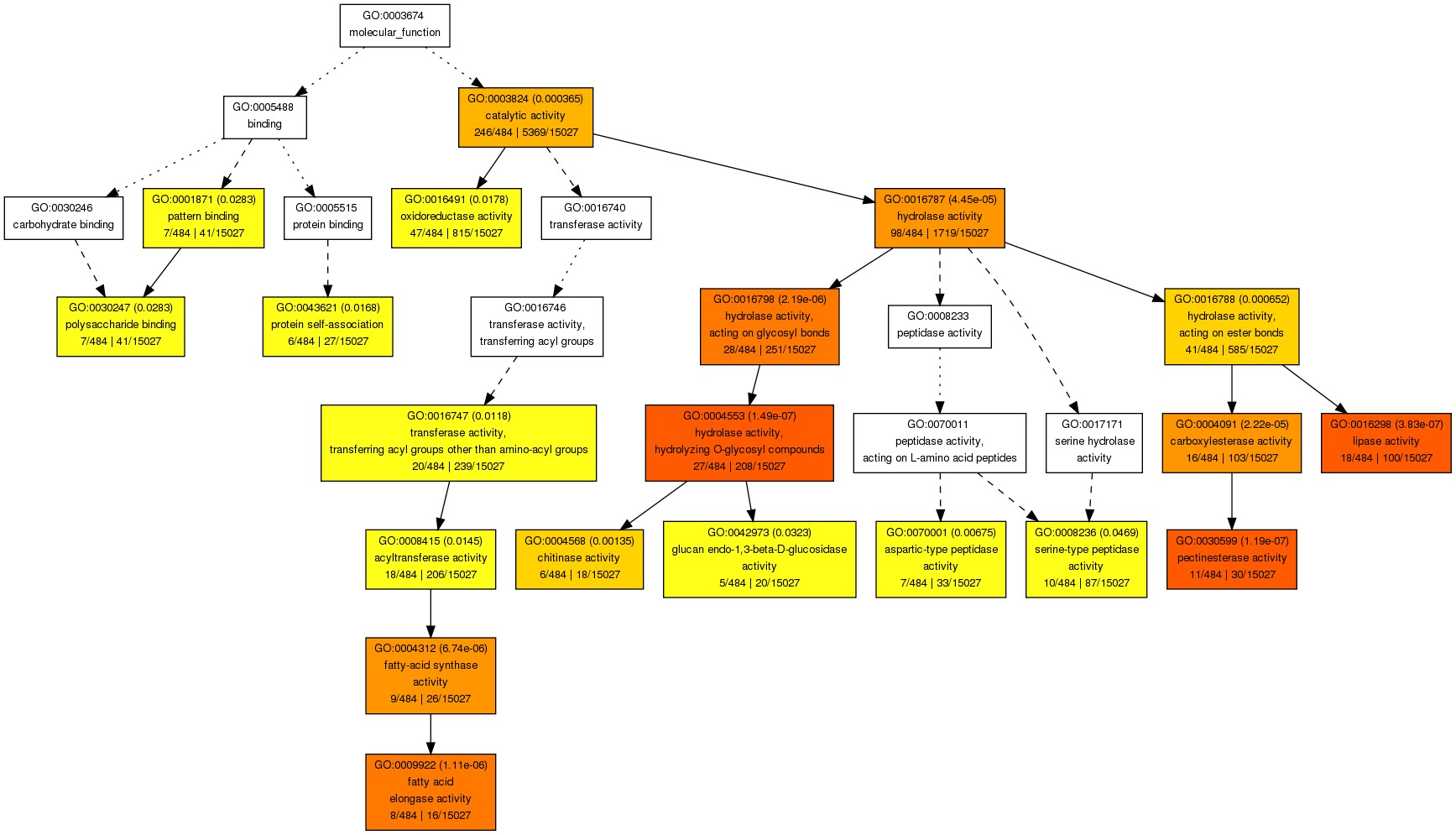


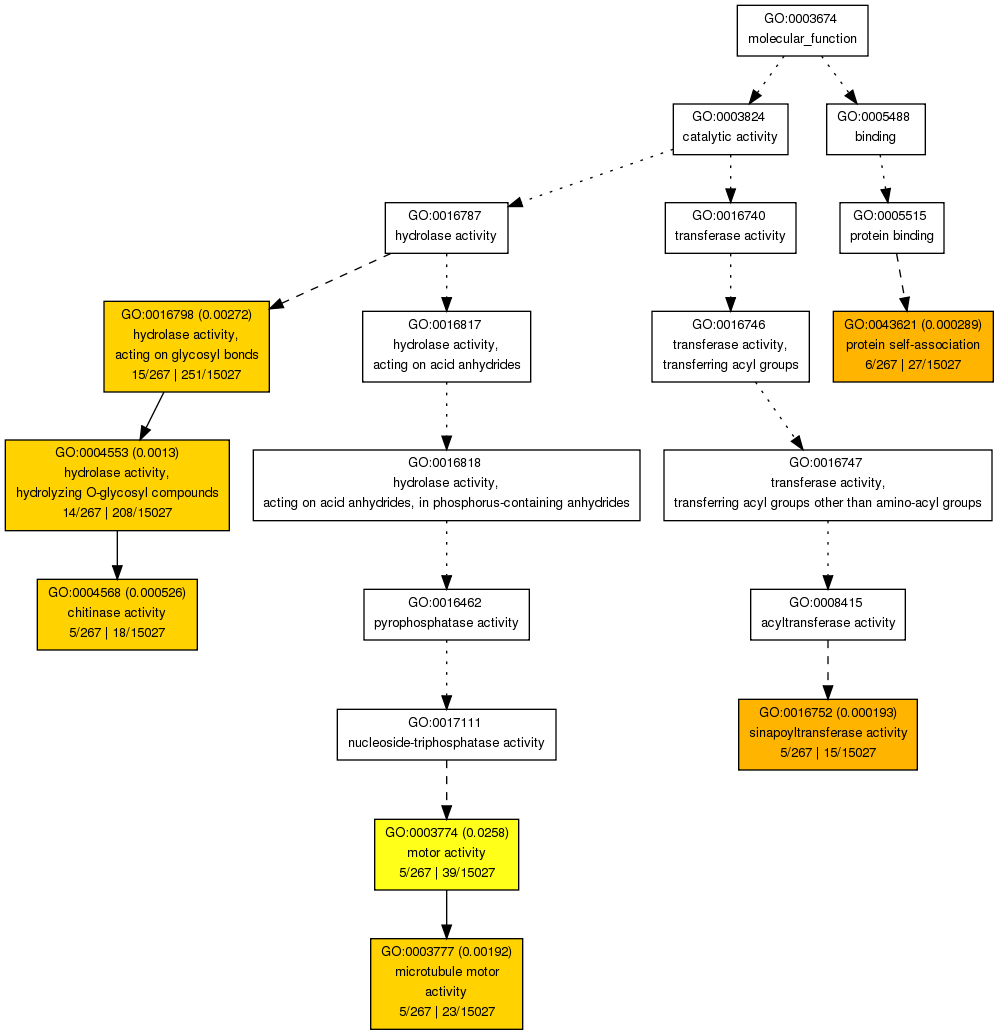


1. HBMD vs HSMD


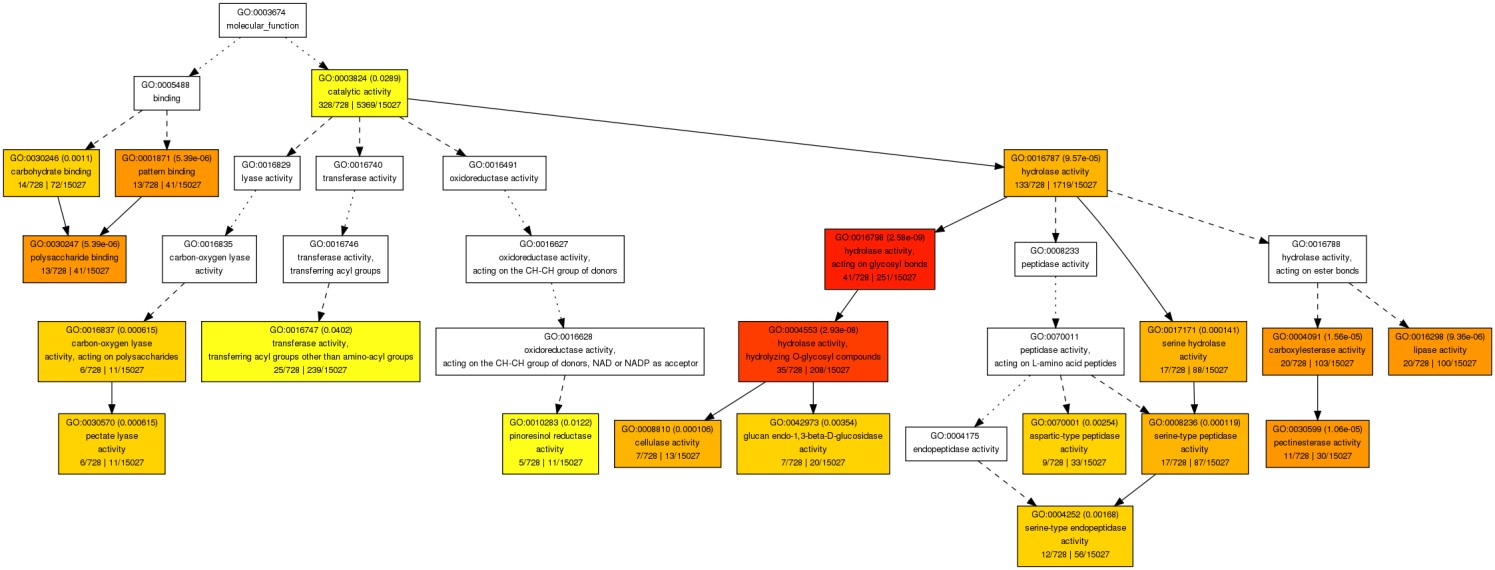


Molecular Function (Down-regulated)

1. HBA vs HSA


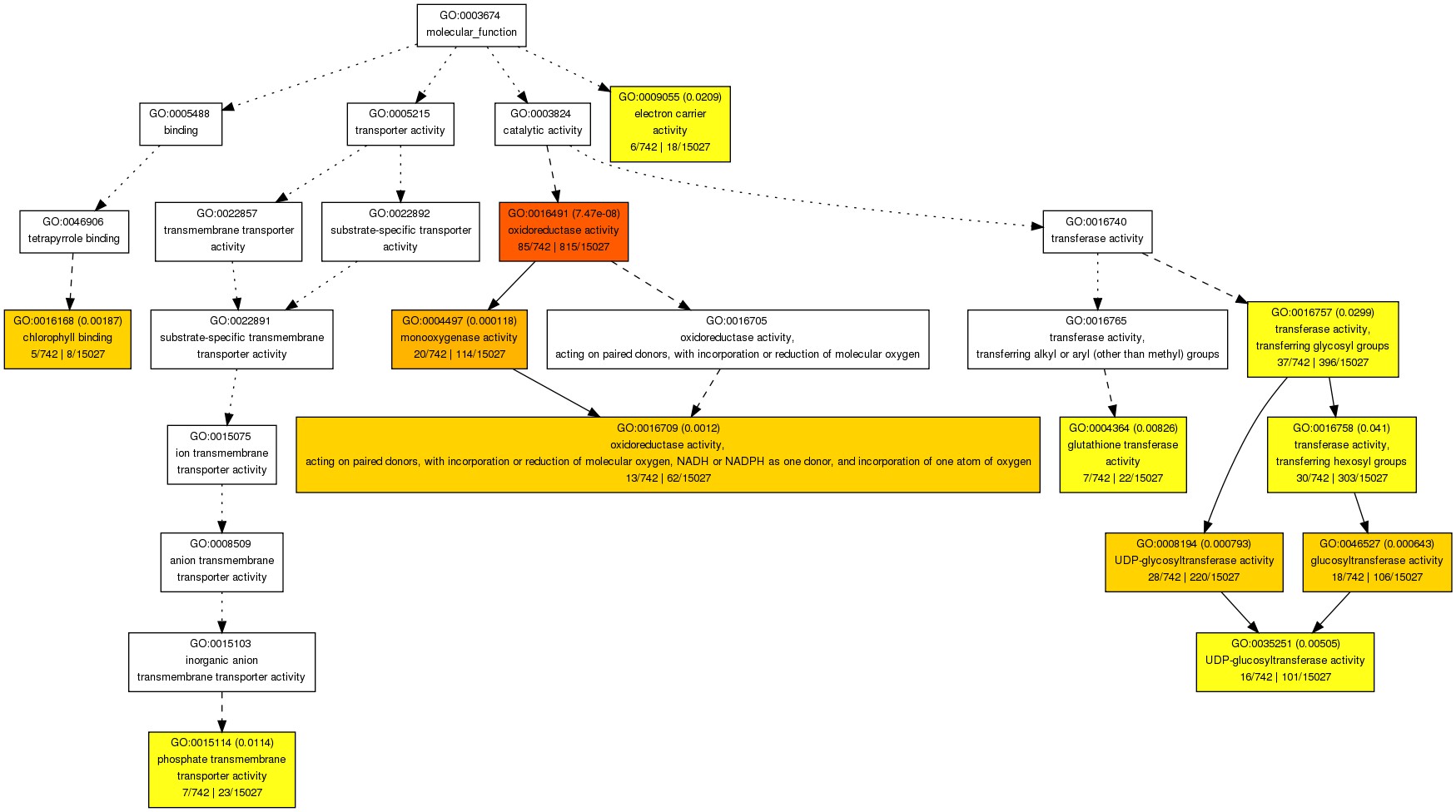


1. HBD vs HSD


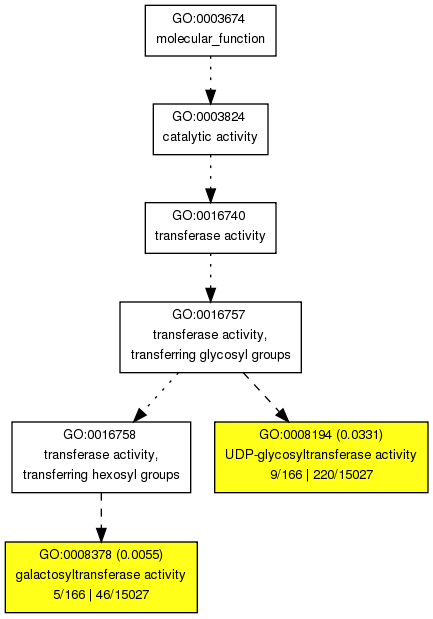


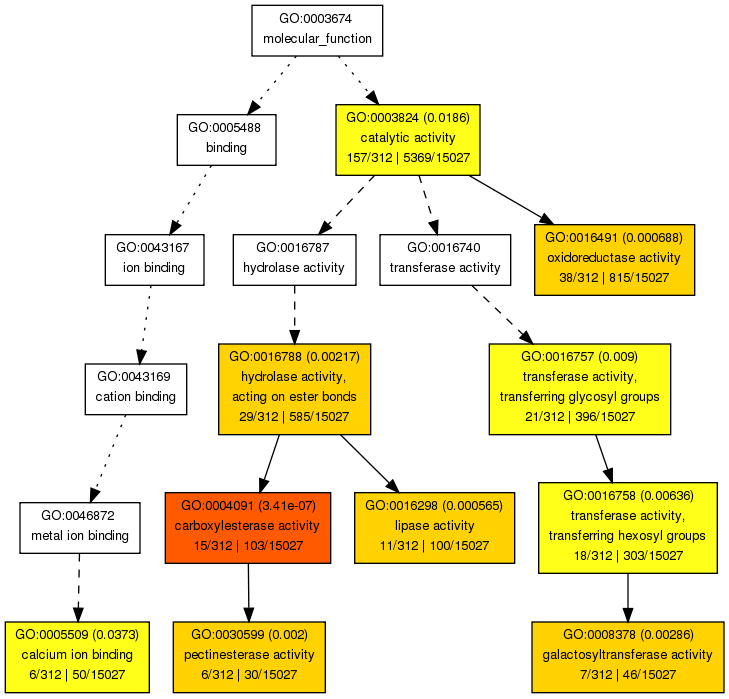


h. HBMD vs HSMD


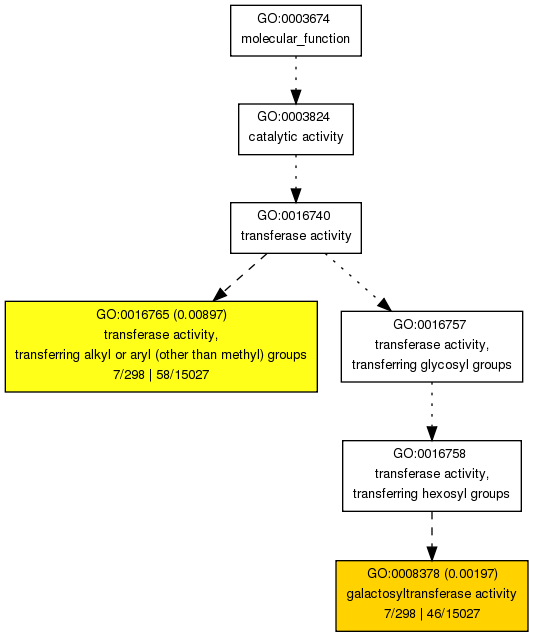


1. Biological Process (Up-regulated)
2. HBA vs HSA


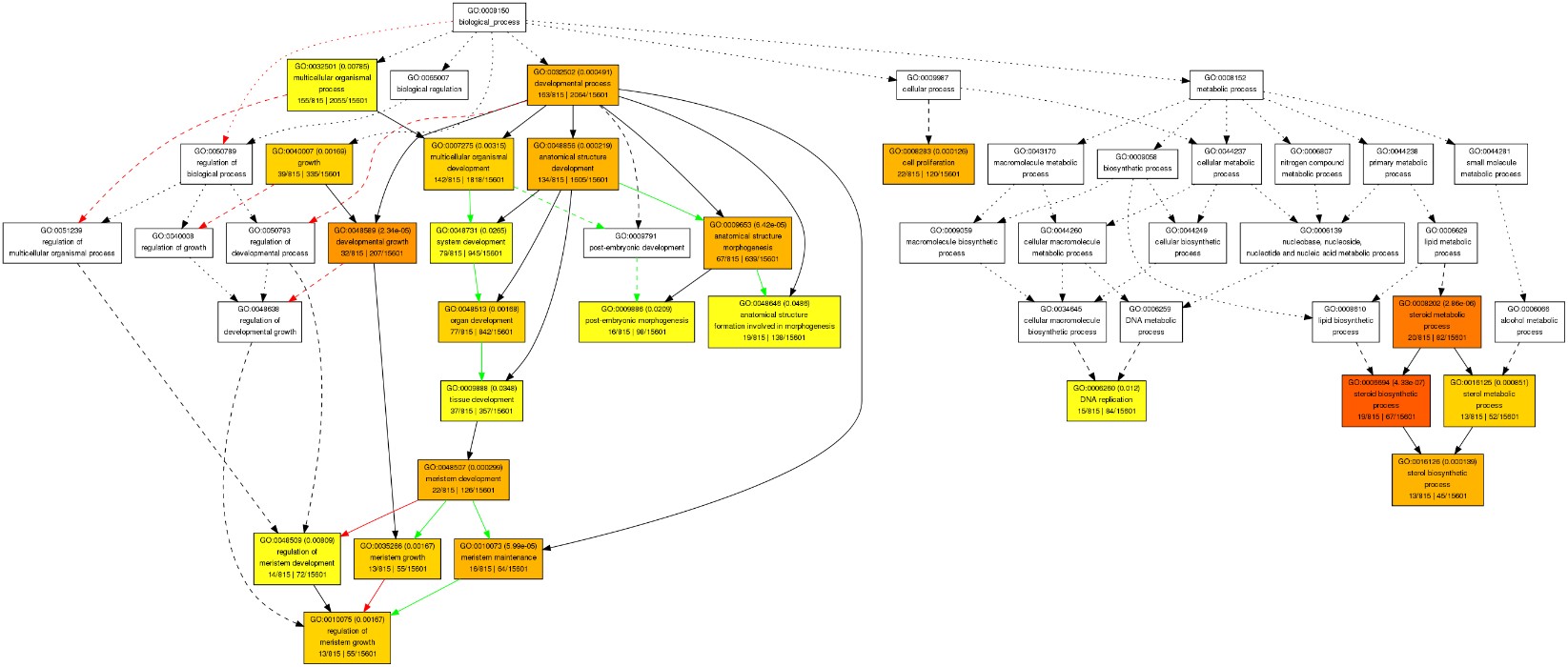


1. HBD vs HSD


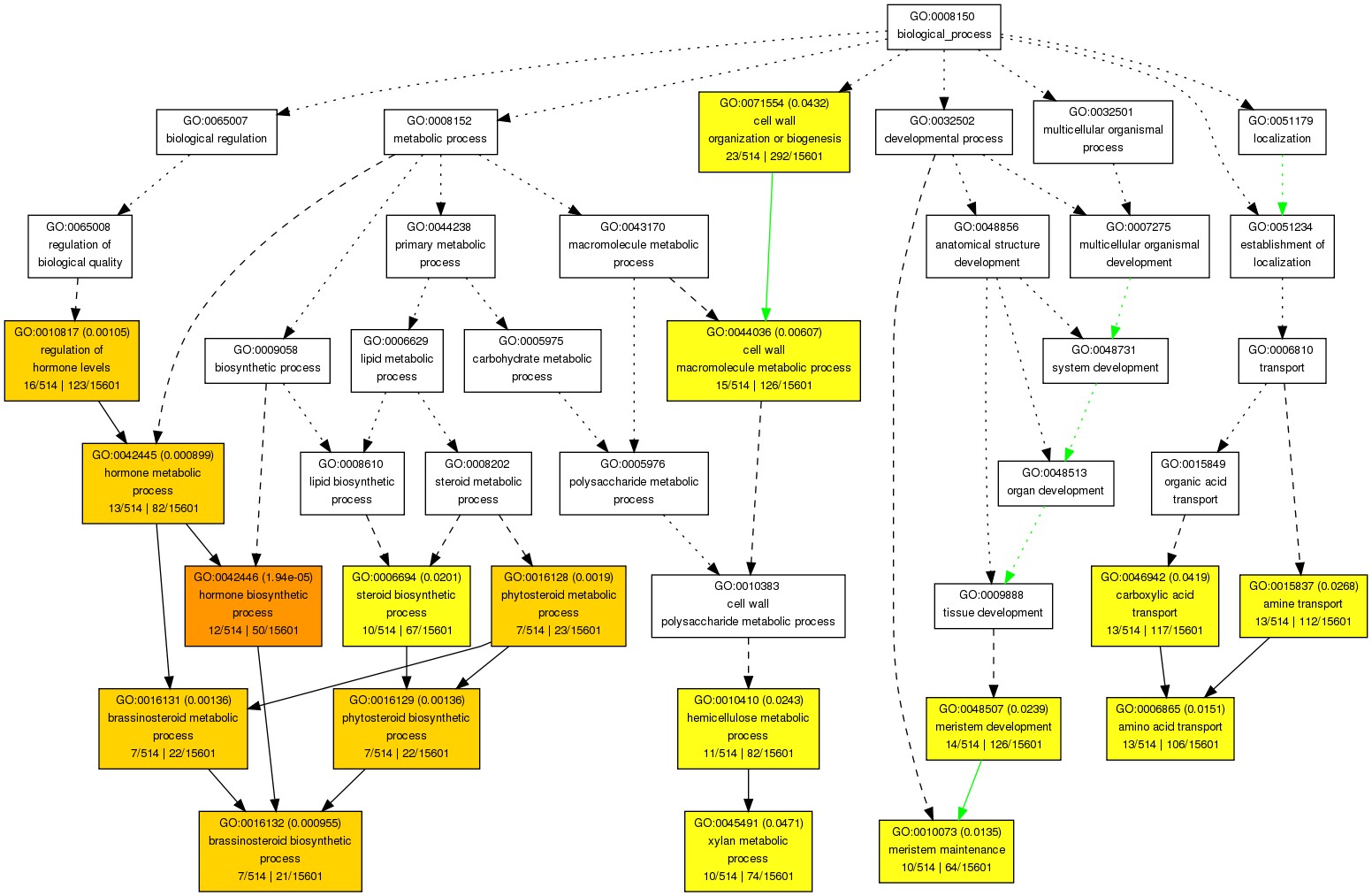


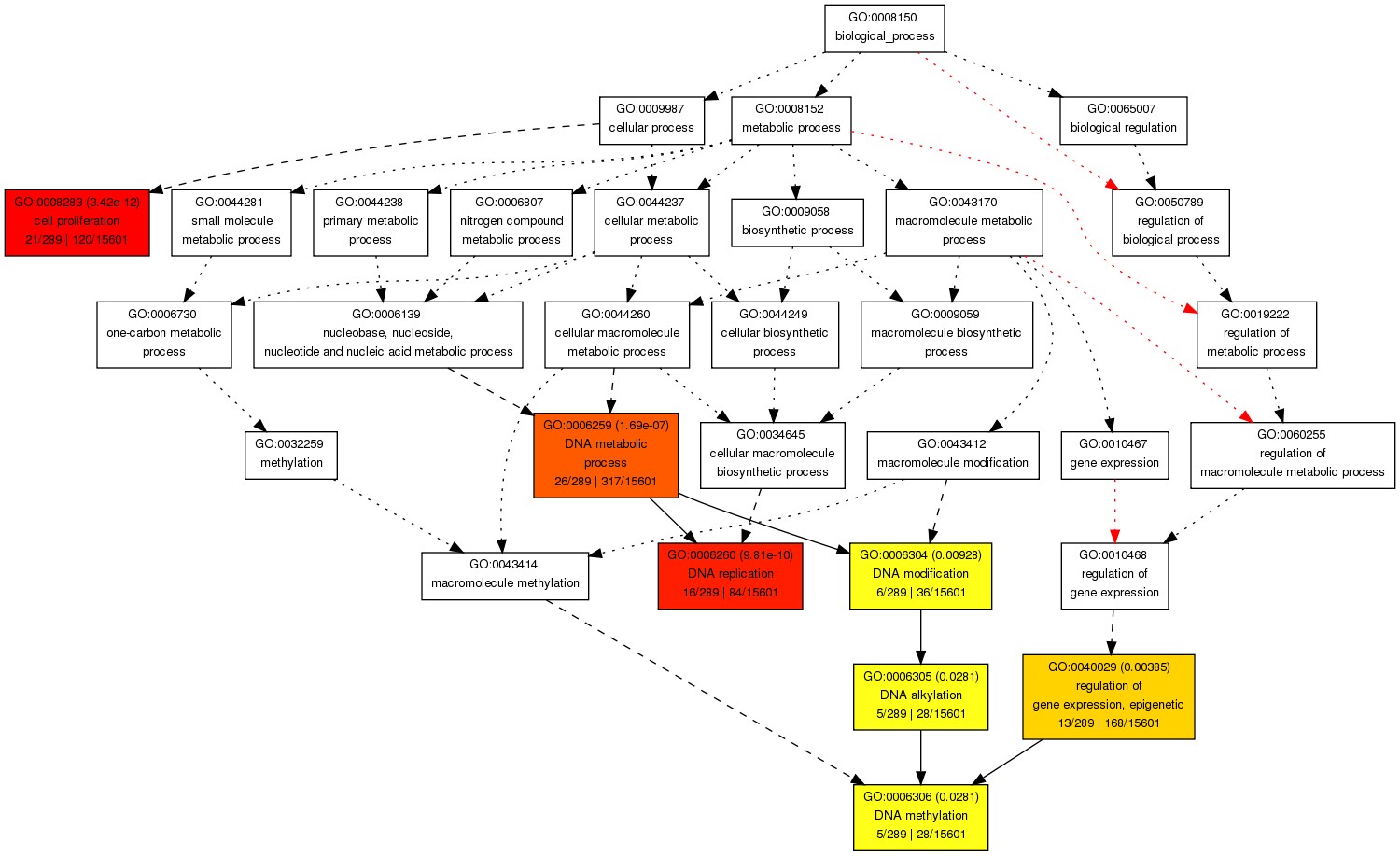


1. HBMD vs HSMD


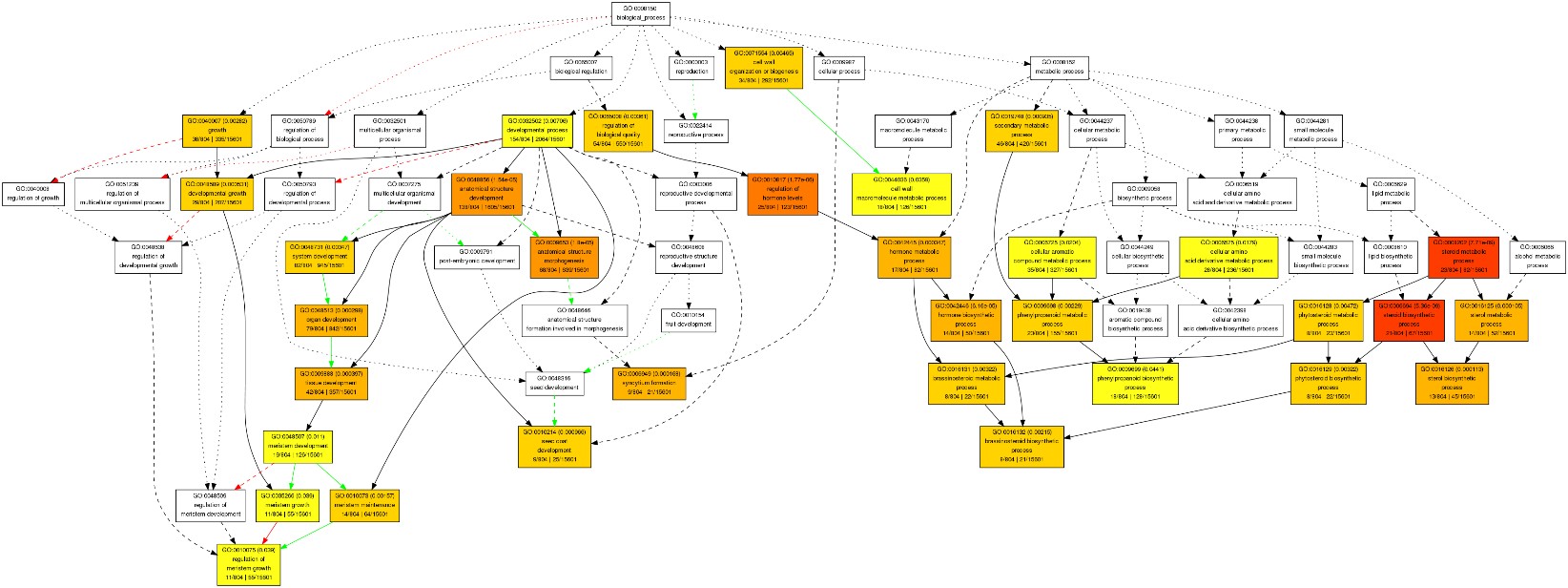


Biological Process (Down-regulated)

1. HBA vs HSA


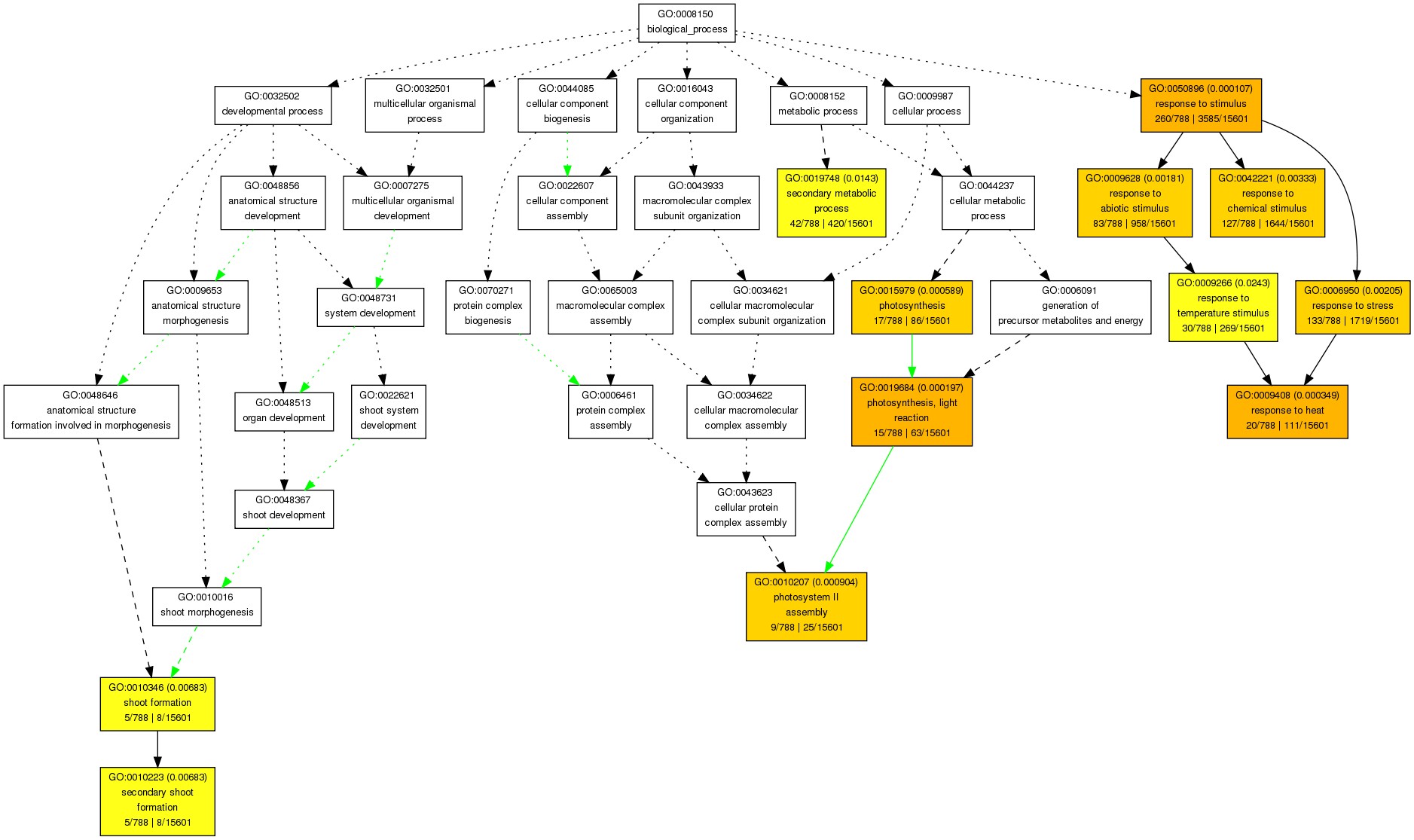


1. HBD vs HSD


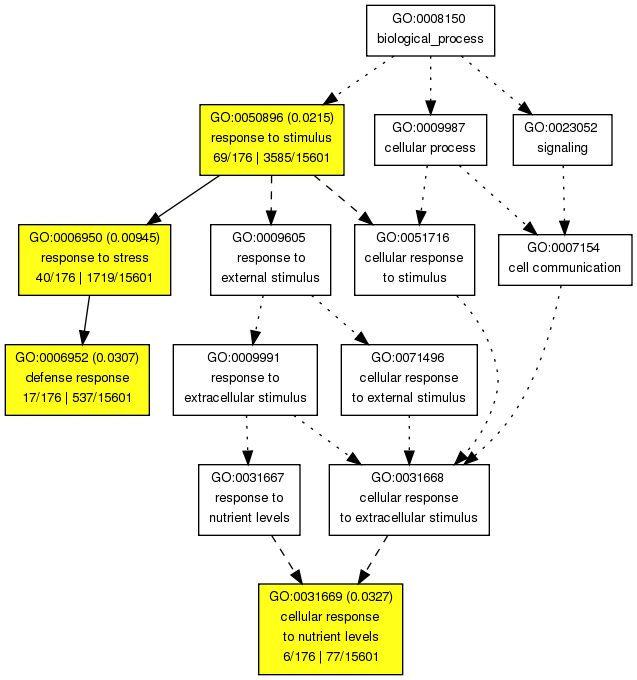


**g. HBMA vs HSMA**

G0:0008150

biological_process

.... .

.----"'-----,·A....r -.

G0:0008152

metabolic process

G0:0009987

cellular process

G0:0016043

cellular component organization

G0:0044085

cellular component

biogenesis

.... **t** ....

G0:0044237

cellular metabolic process

'


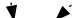


G0:0043933 G0:0022607 G0:0070271

macromolecular complex cellular component protein complex

subunit organization assembly biogenesis


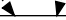
.... **t**


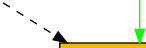

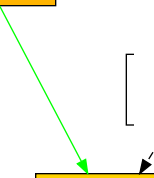

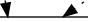

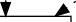


G0:0006091

generation of

precursor metabolites and energy

G0:0015979 (0.00101)

photosynthesis

11/353 1 86/15601

G0:0019684 (0.000367)

photosynthesis, light reaction

101353 1 63115601

G0:0034621

cellular macromolecular complex subunit organization

G0:0034622

cellular macromolecular complex assembly

G0:0043623

cellular protein complex assembly

G0:0010207 (0.00375)

photosystem II

assembly

6/353 1 25/15601

G0:0065003

macromolecular complex assembly


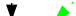


G0:0006461

protein complex assembly

1. Molecular Function (Up-regulated)
   1. TBA vs TSA


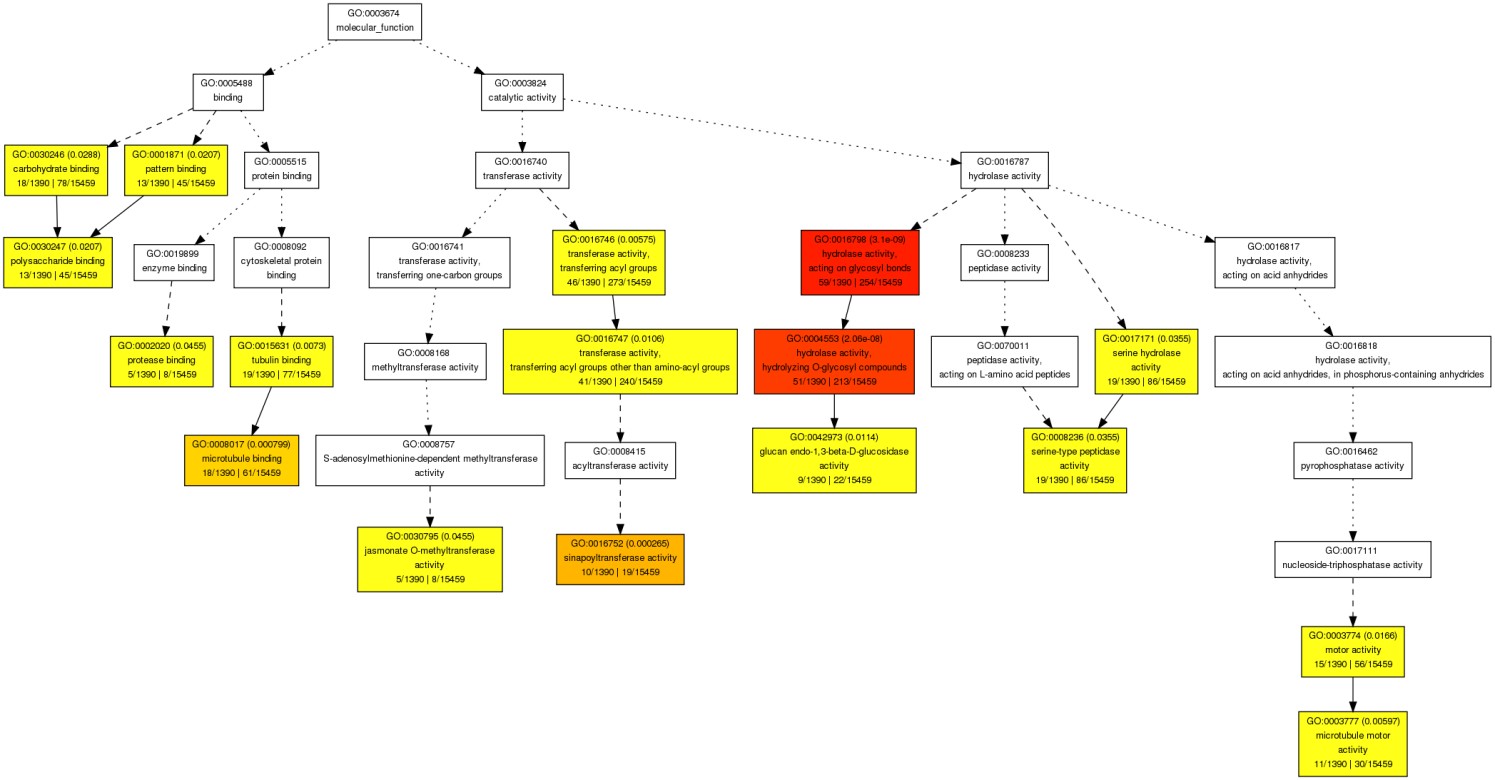


- 1. TBD vs TSD


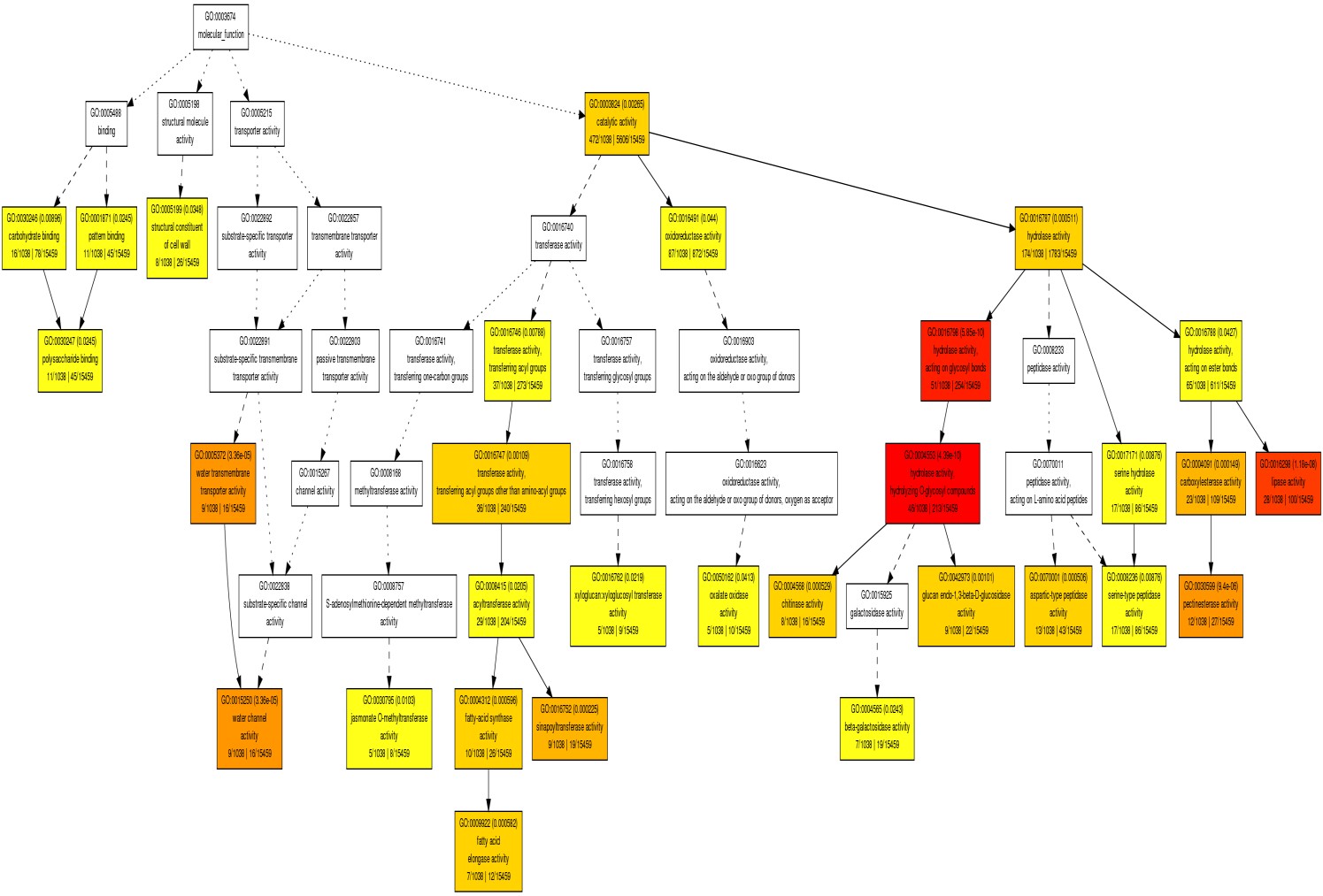


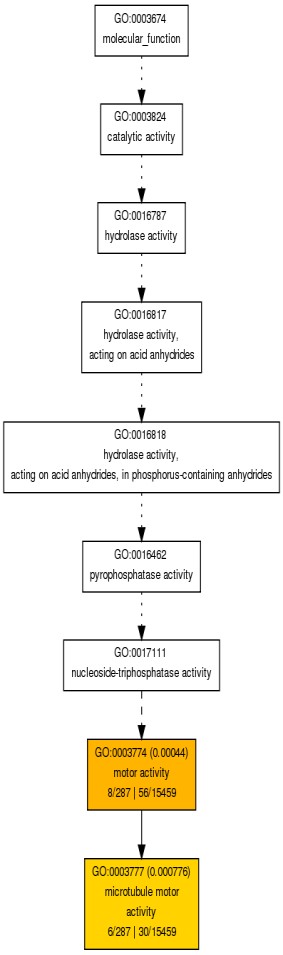


1. TBMD vs TSMD


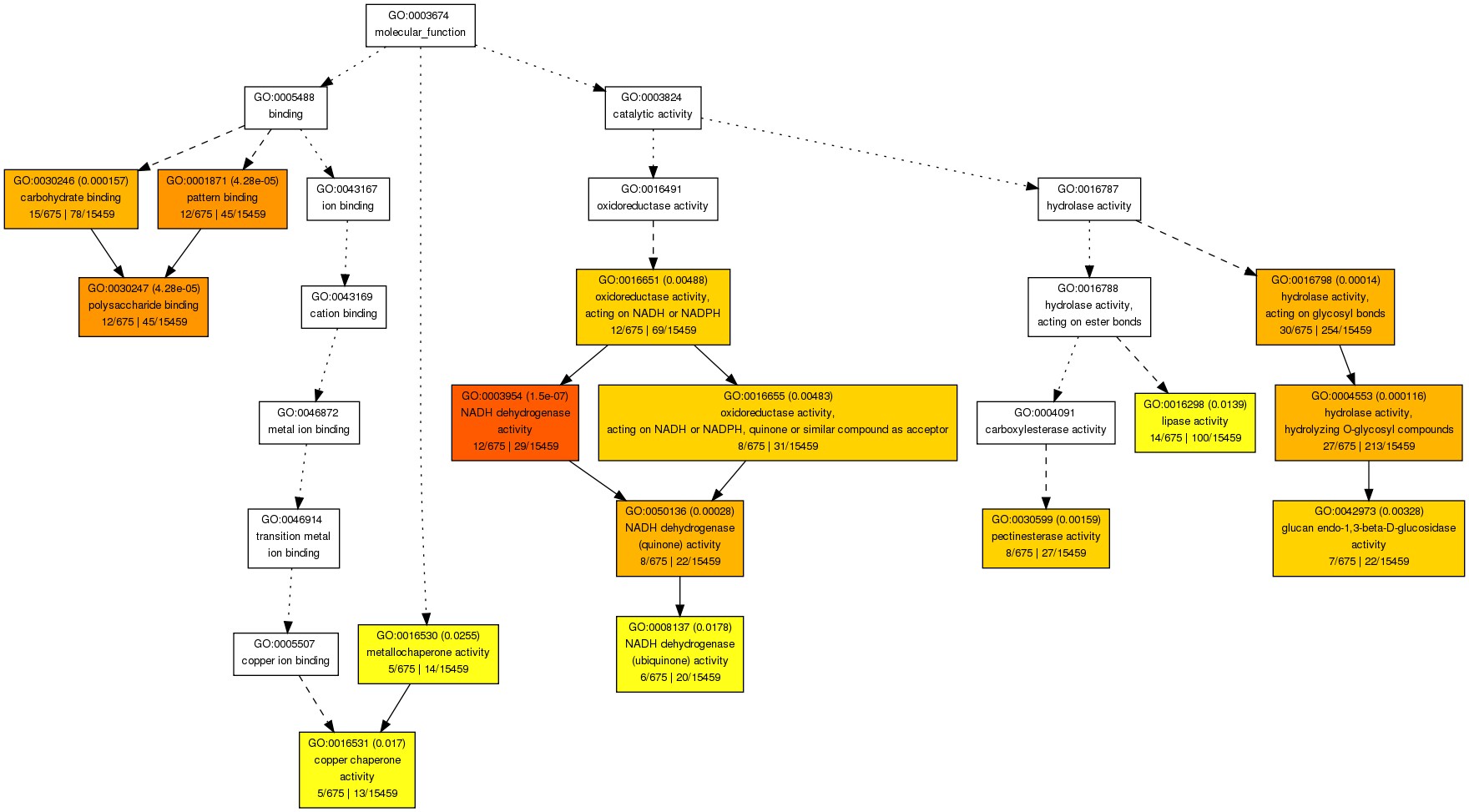


Molecular Function (Down-regulated)

1. TBA vs TSA


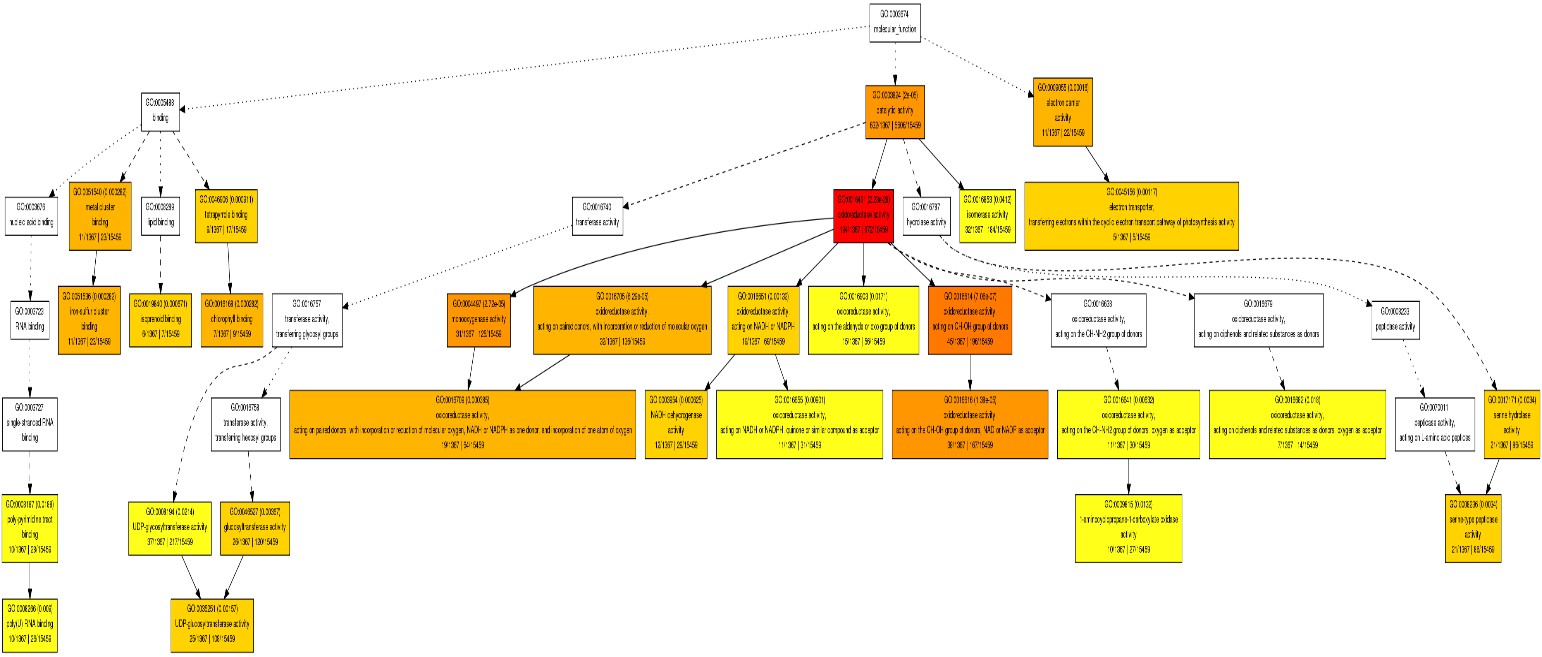


1. TBD vs TSD


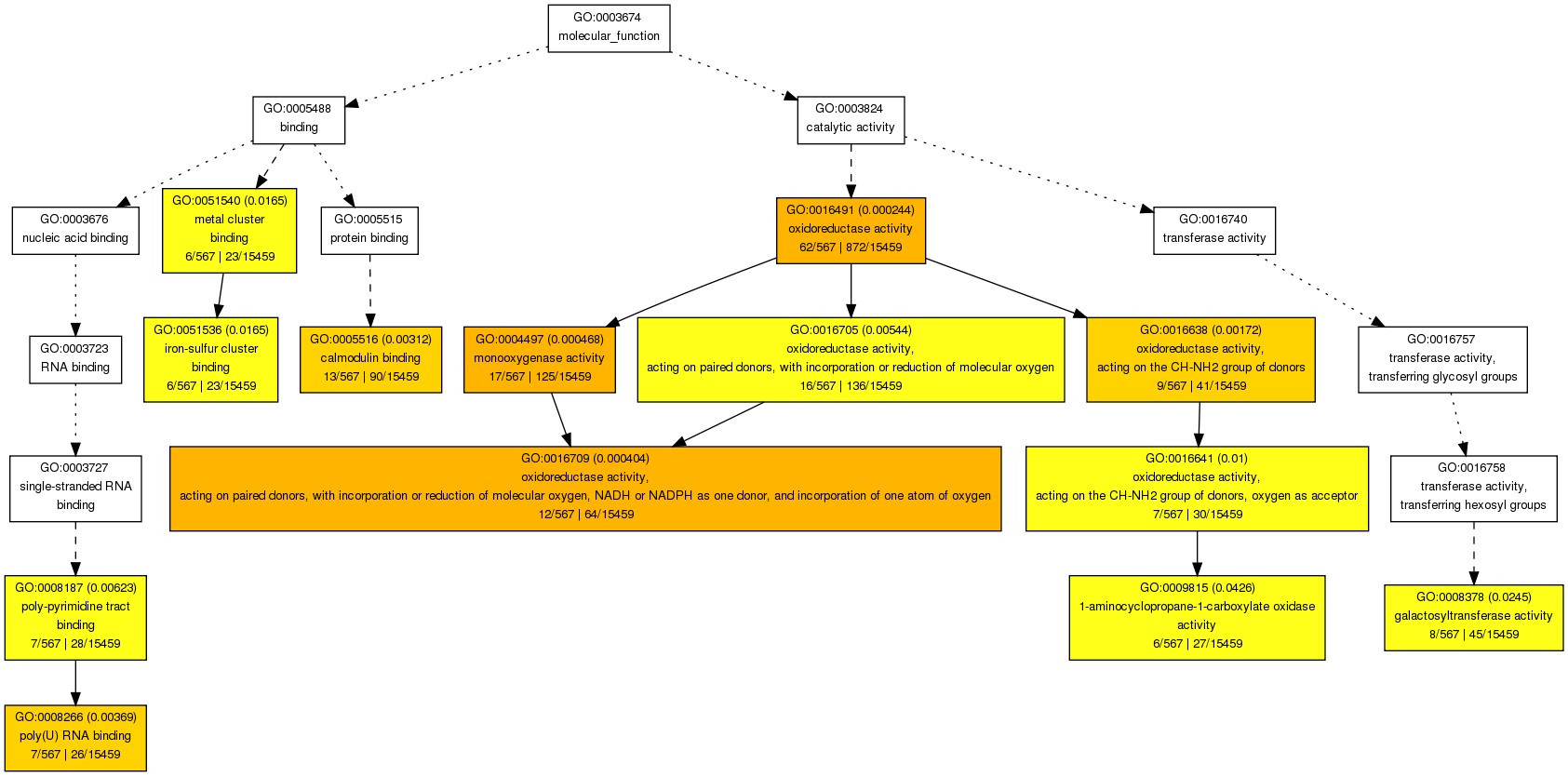


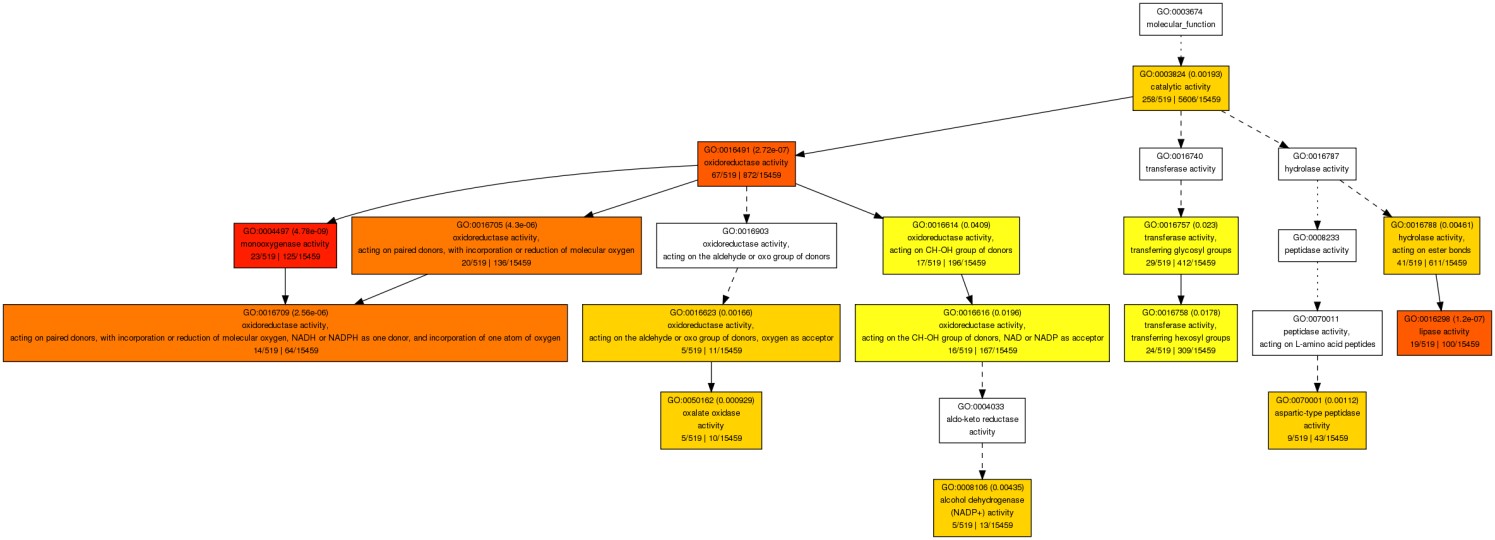


h. TBMD vs TSMD


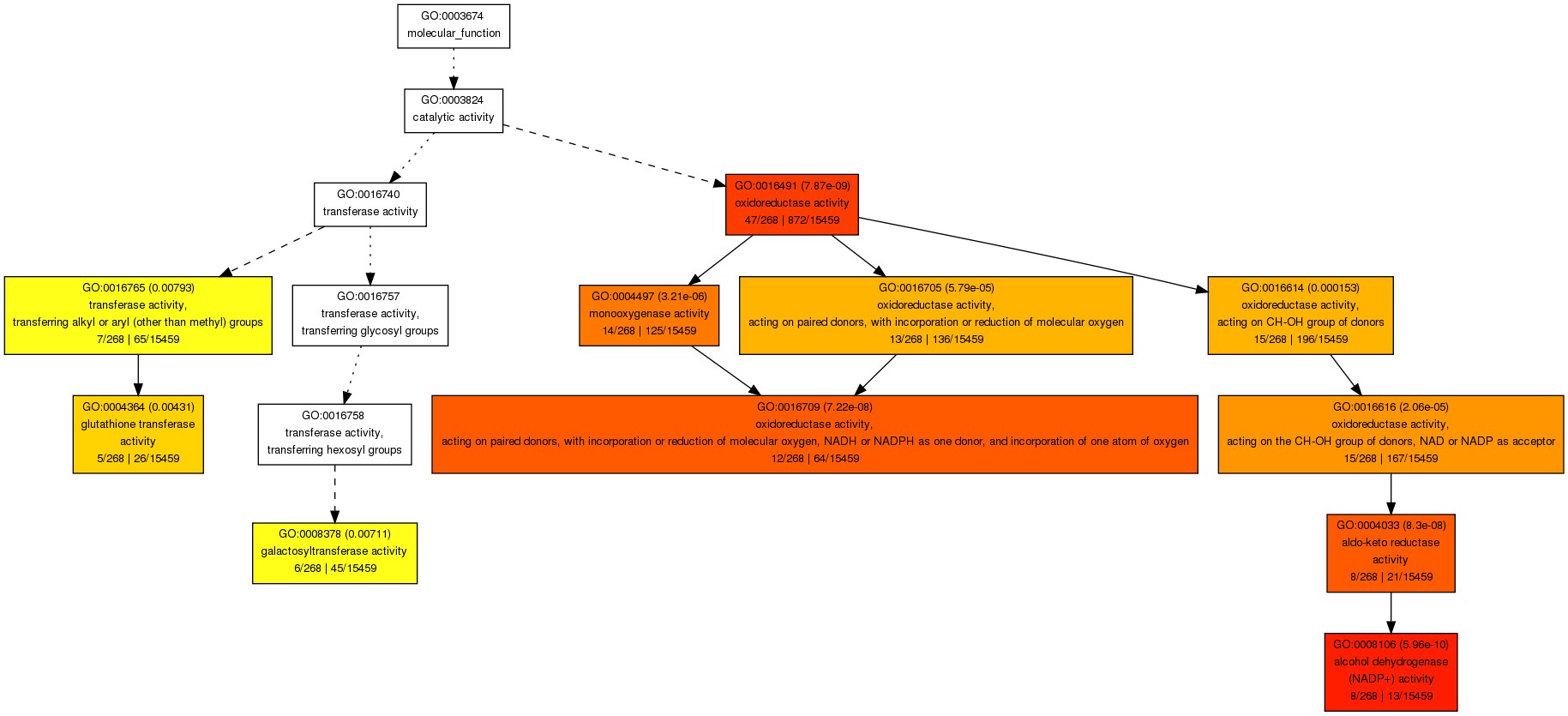


1. Biological Process (Up-regulated)
2. TBA vs TSA


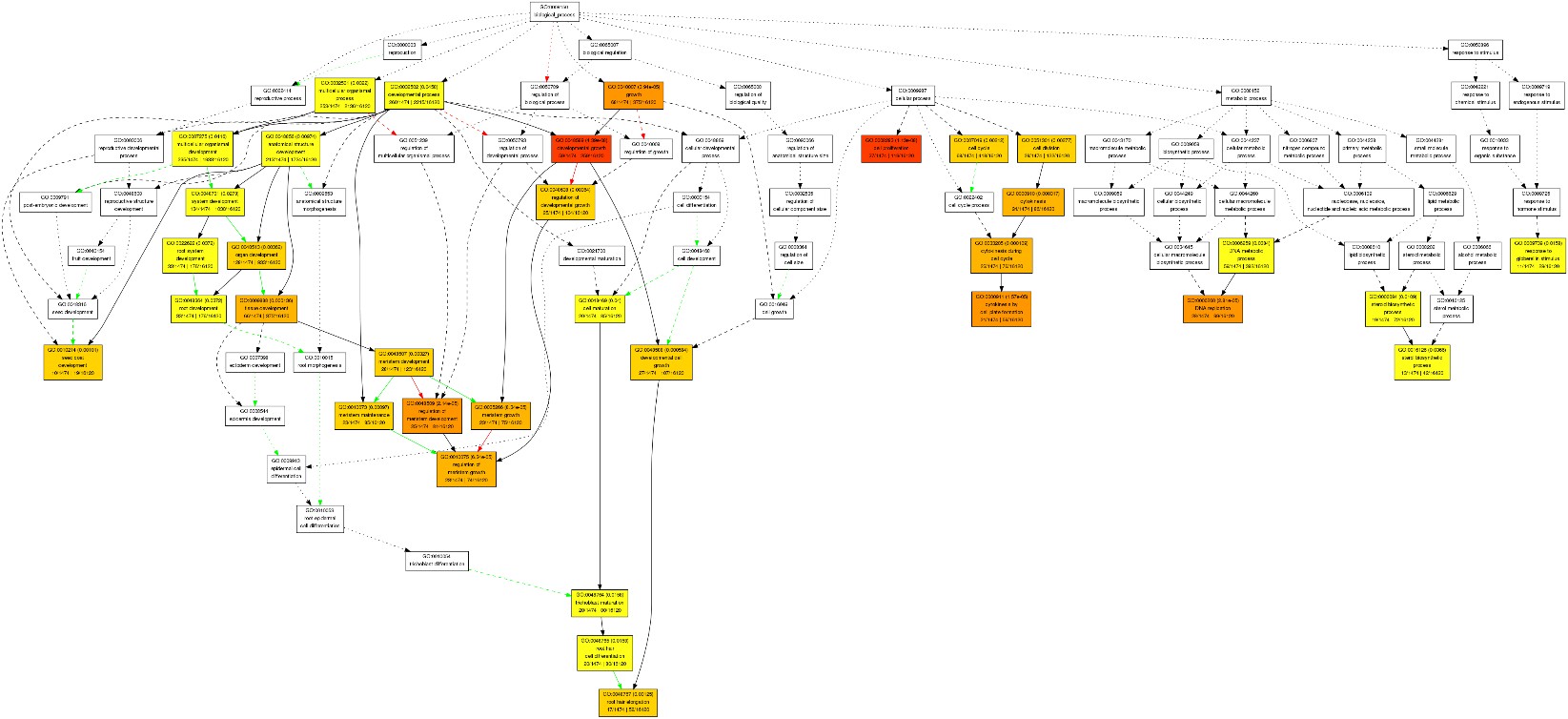


1. TBD vs TSD


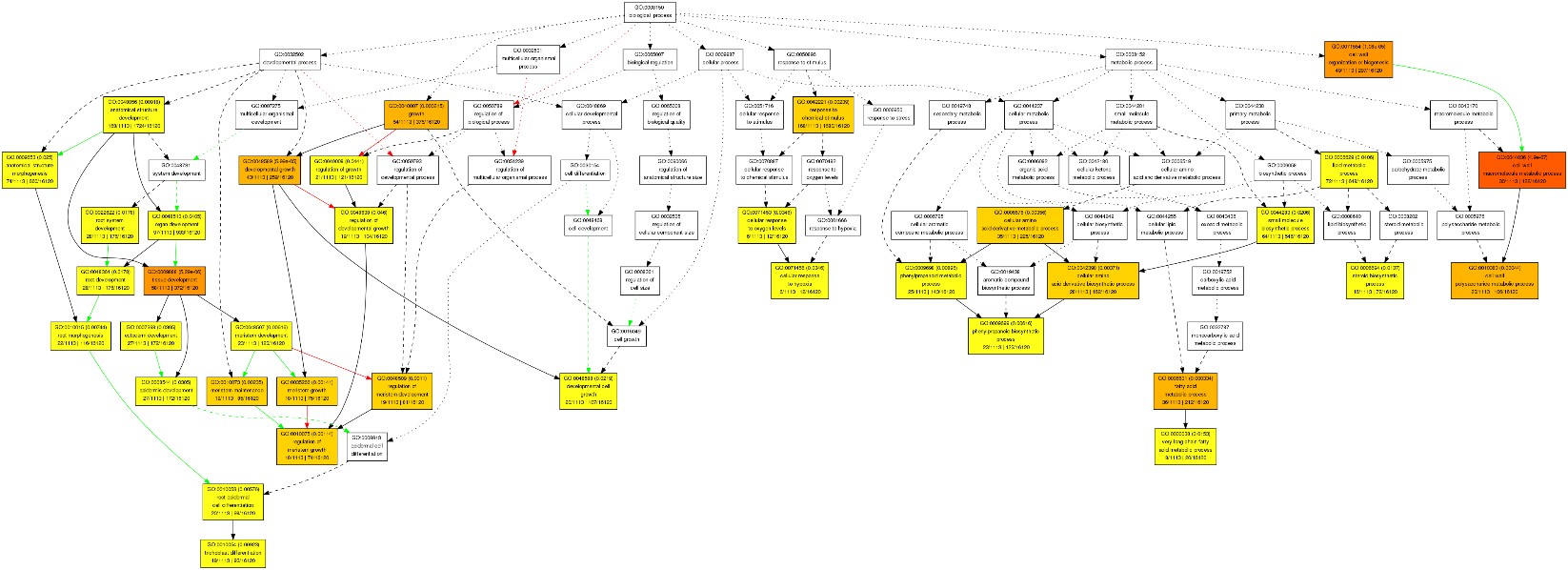


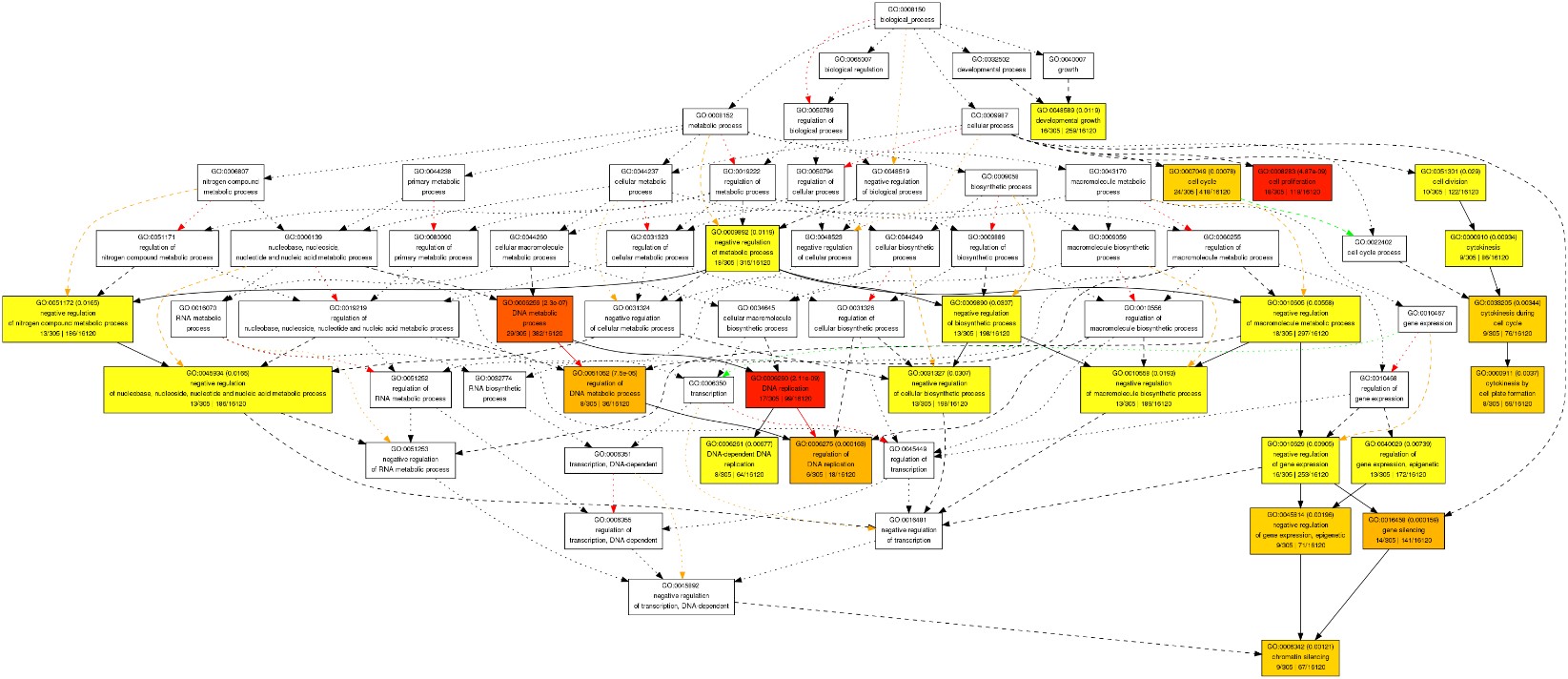


1. TBMD vs TSMD


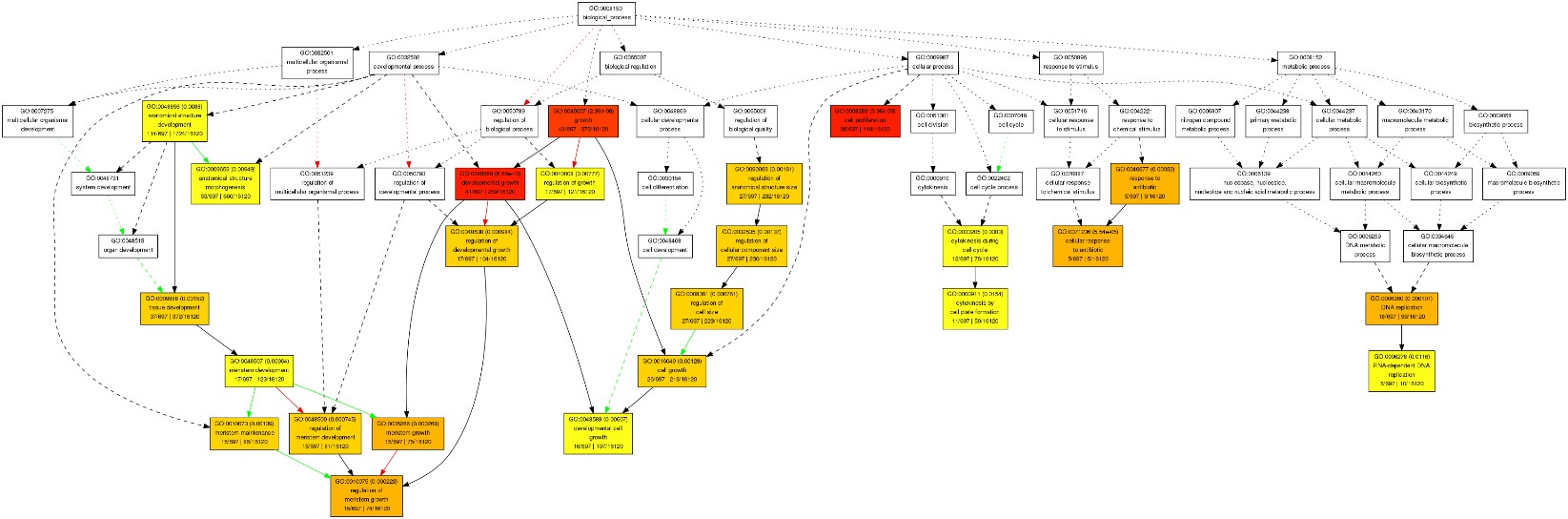


Biological Process (Down-regulated)

1. TBA vs TSA


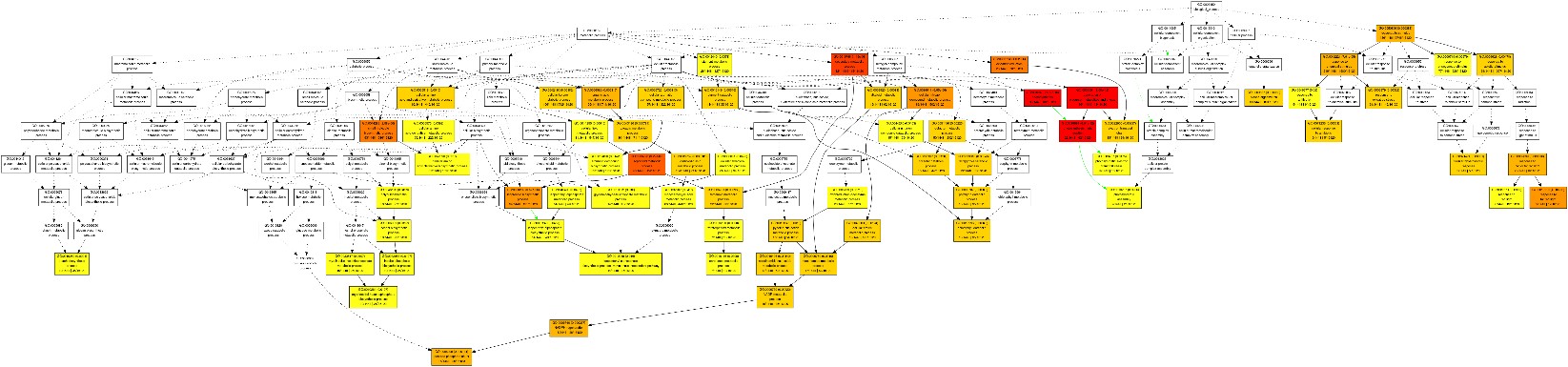


1. TBD vs TSD


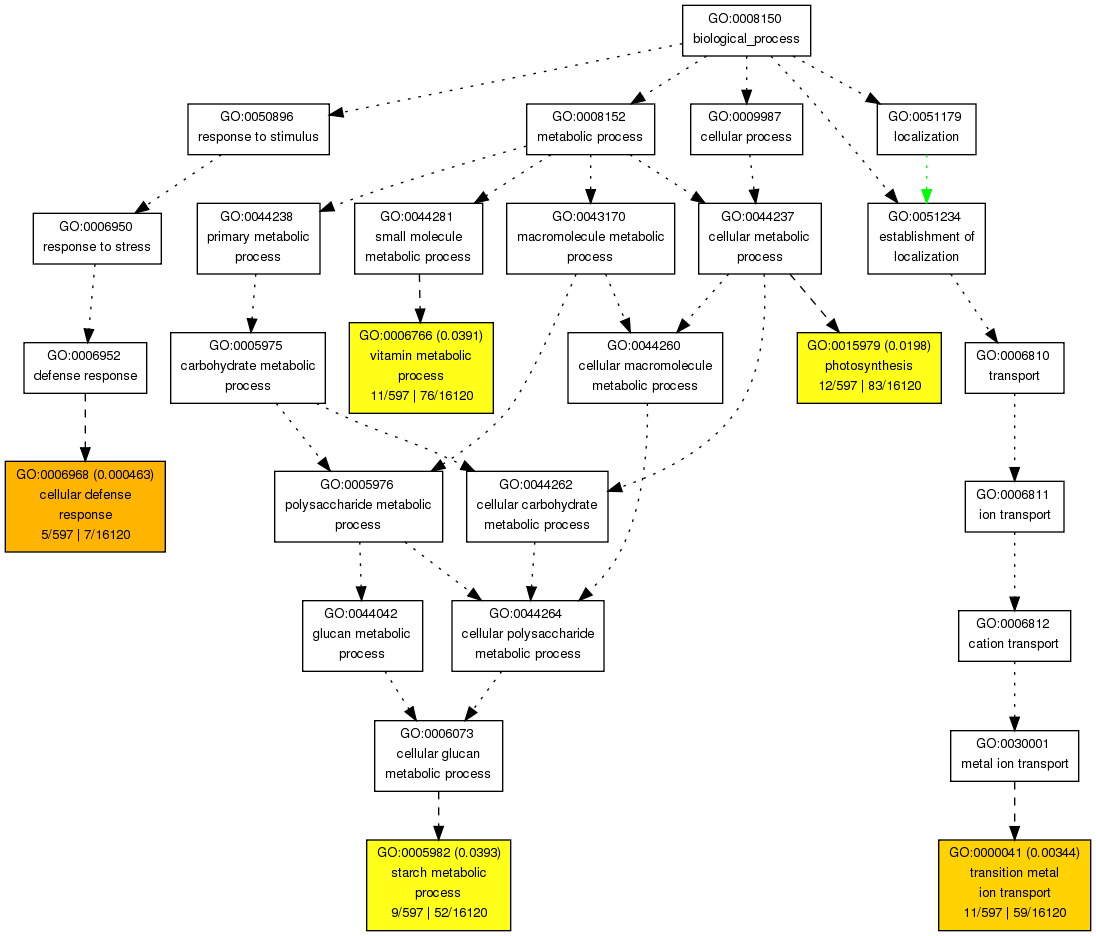


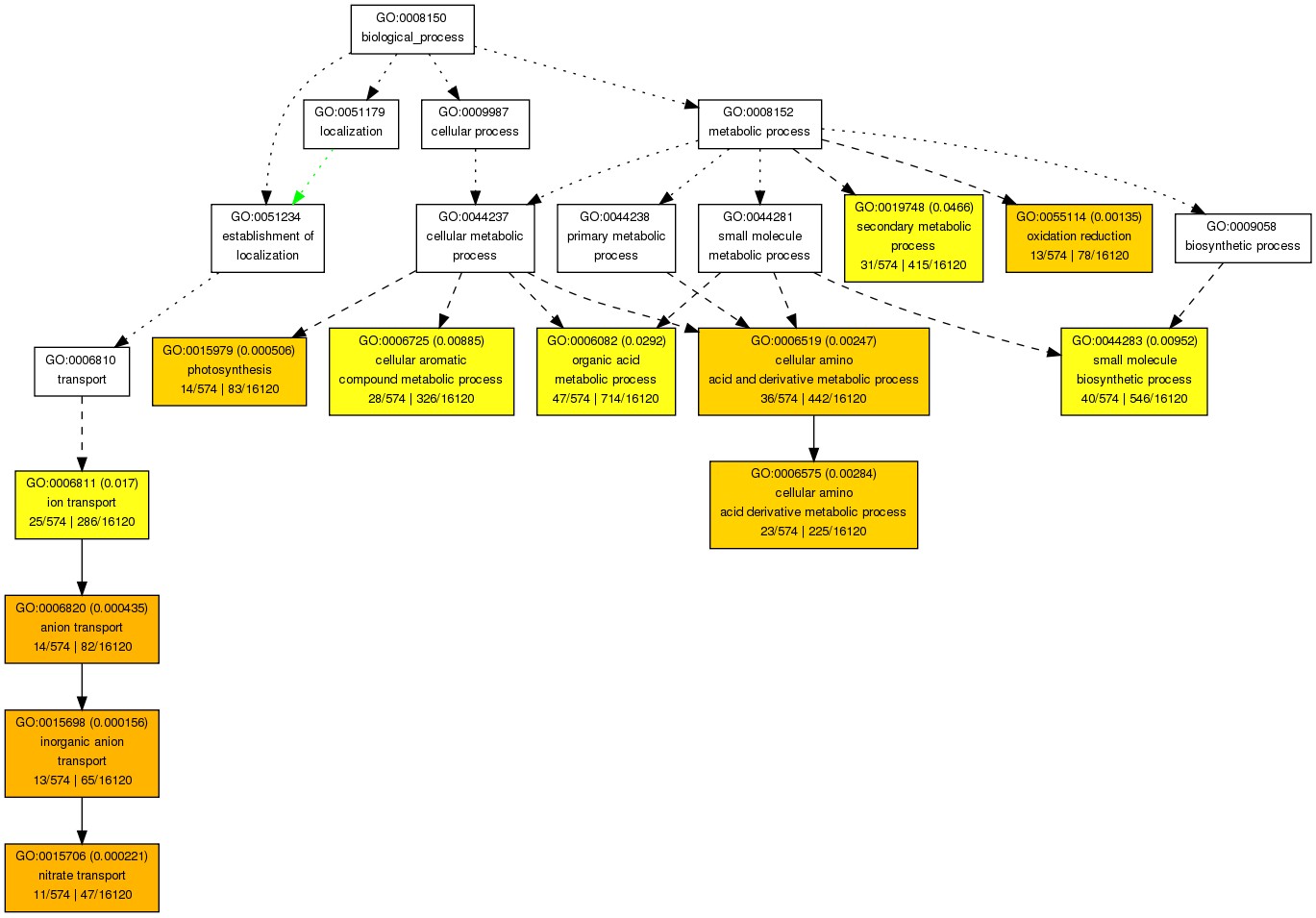


h. TBMD vs TSMD


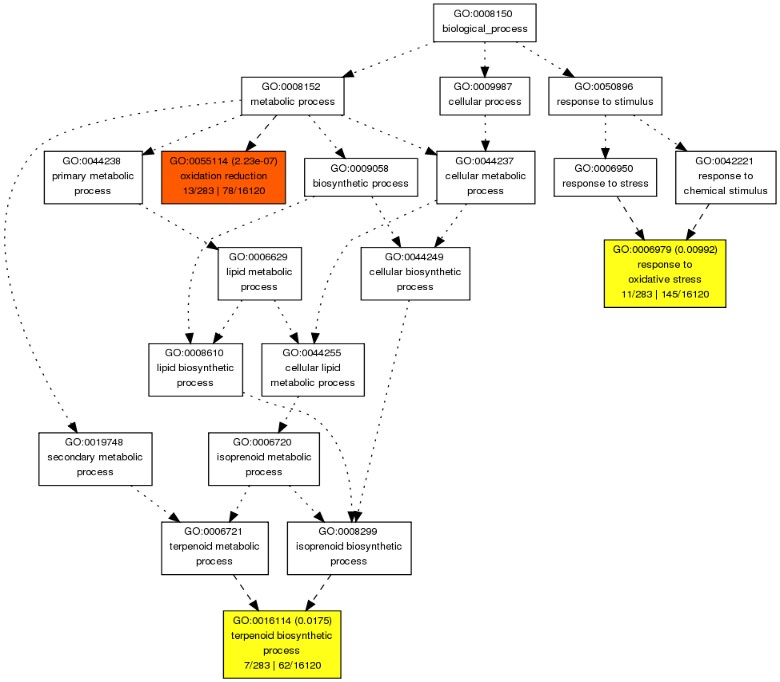


1. Molecular Function (Up-regulated)
2. UBA vs USA


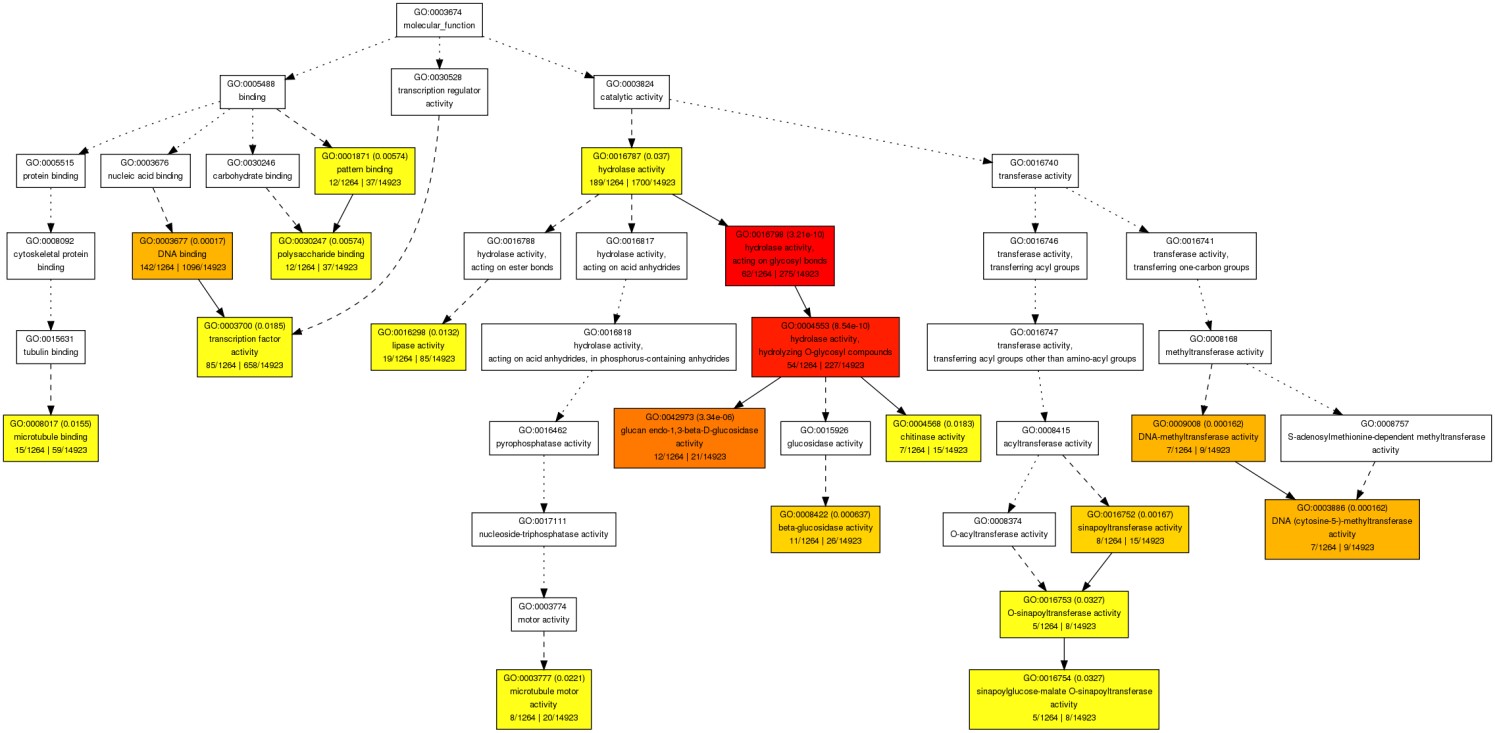


1. UBD vs USD


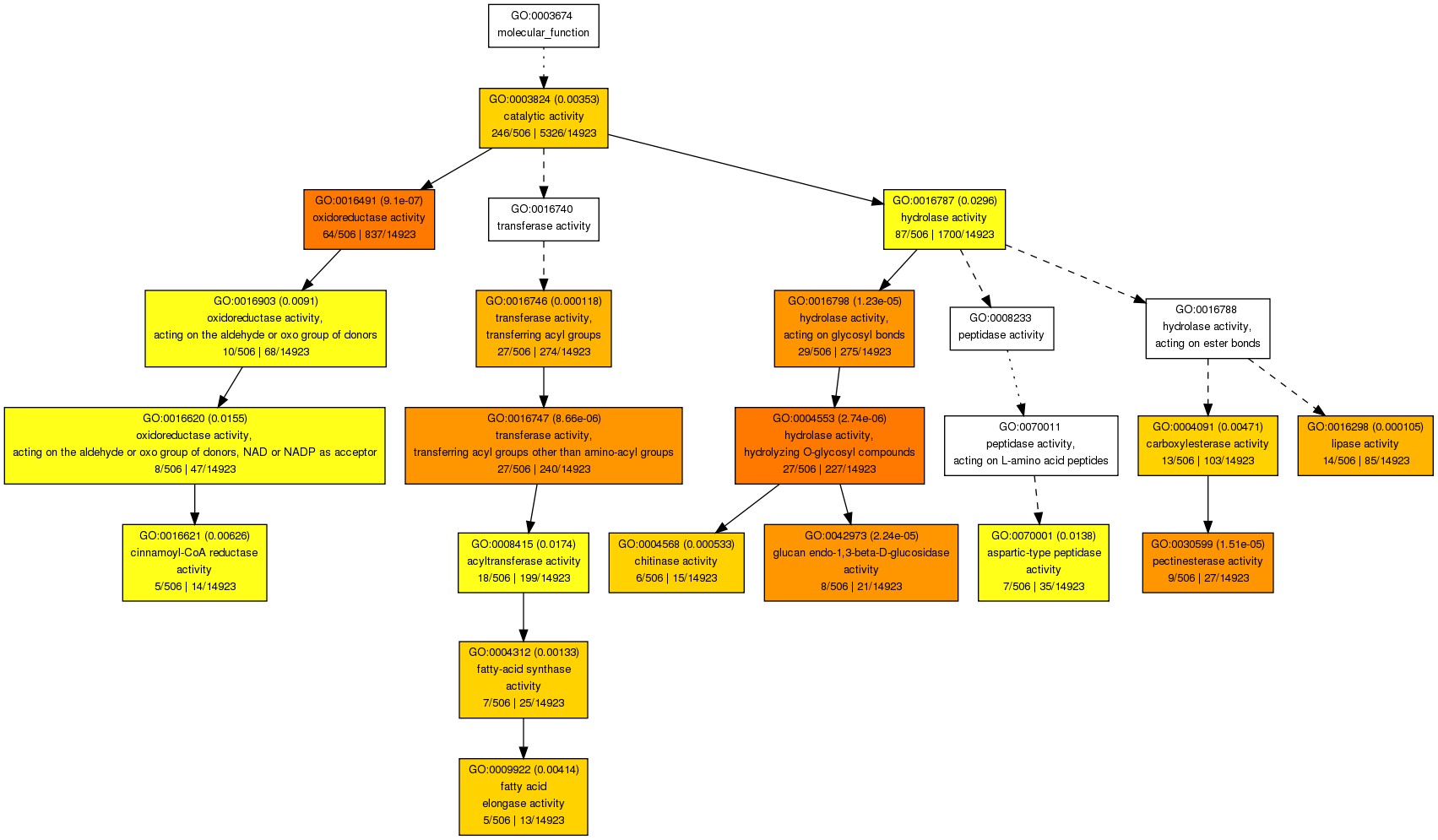


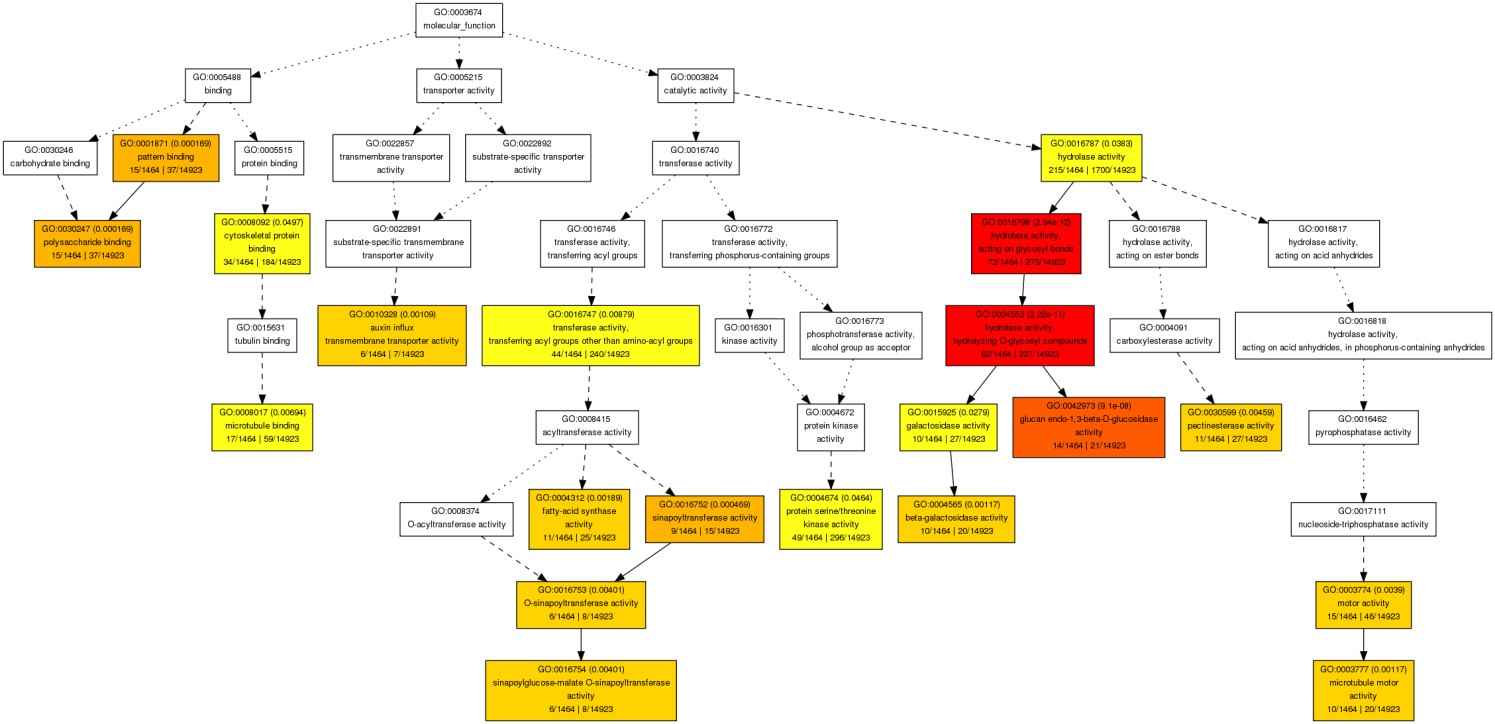


1. UBMD vs USMD


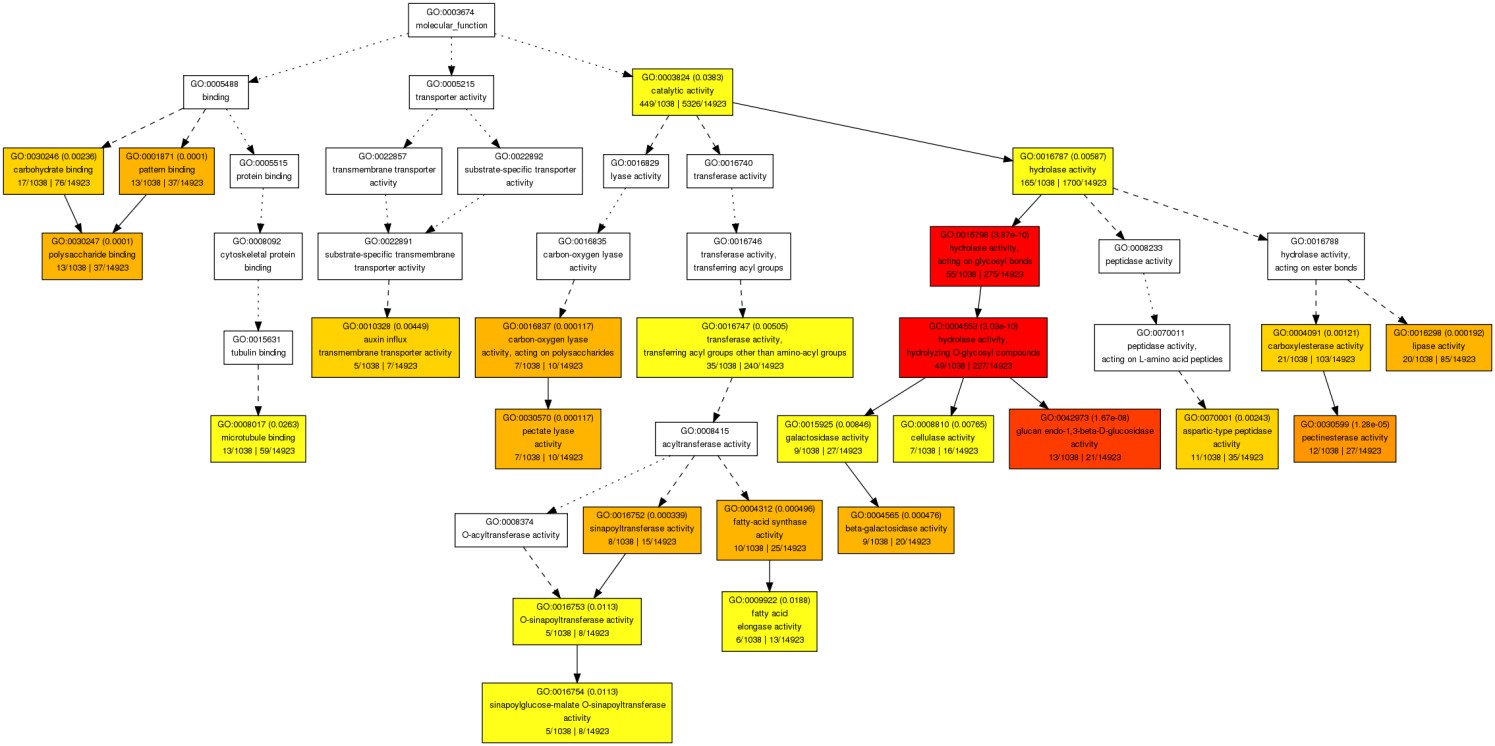


Molecular Function (Down-regulated)

1. UBA vs USA


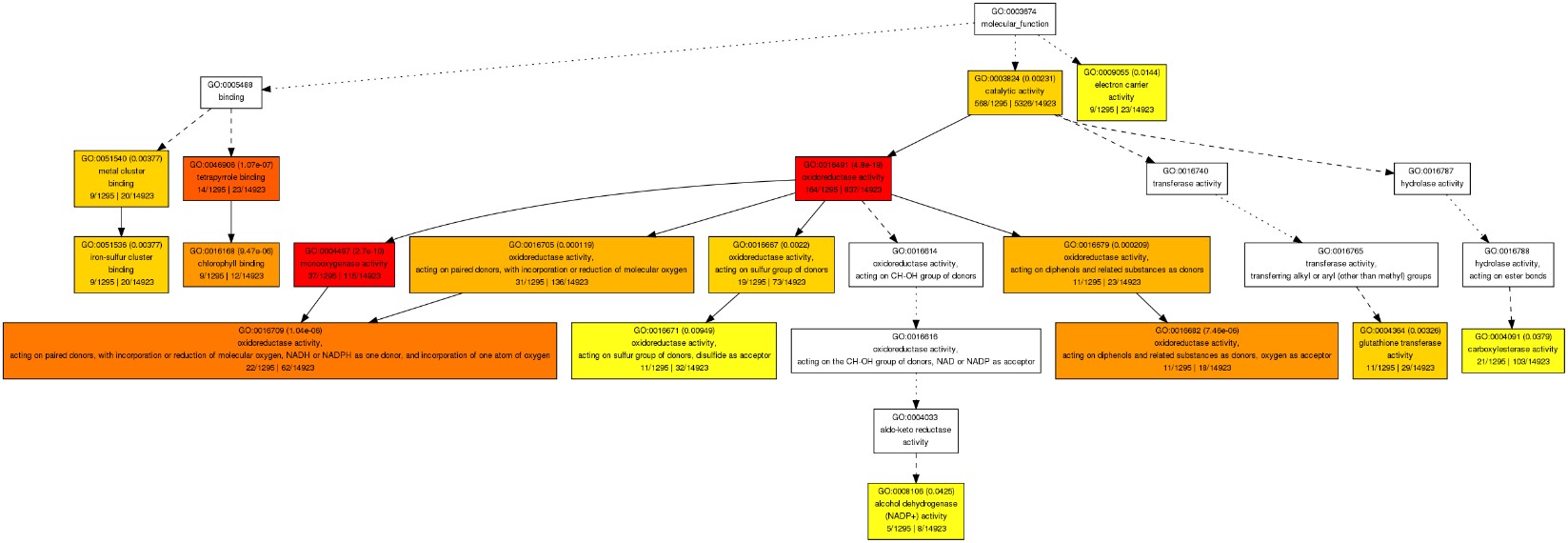


1. UBD vs USD


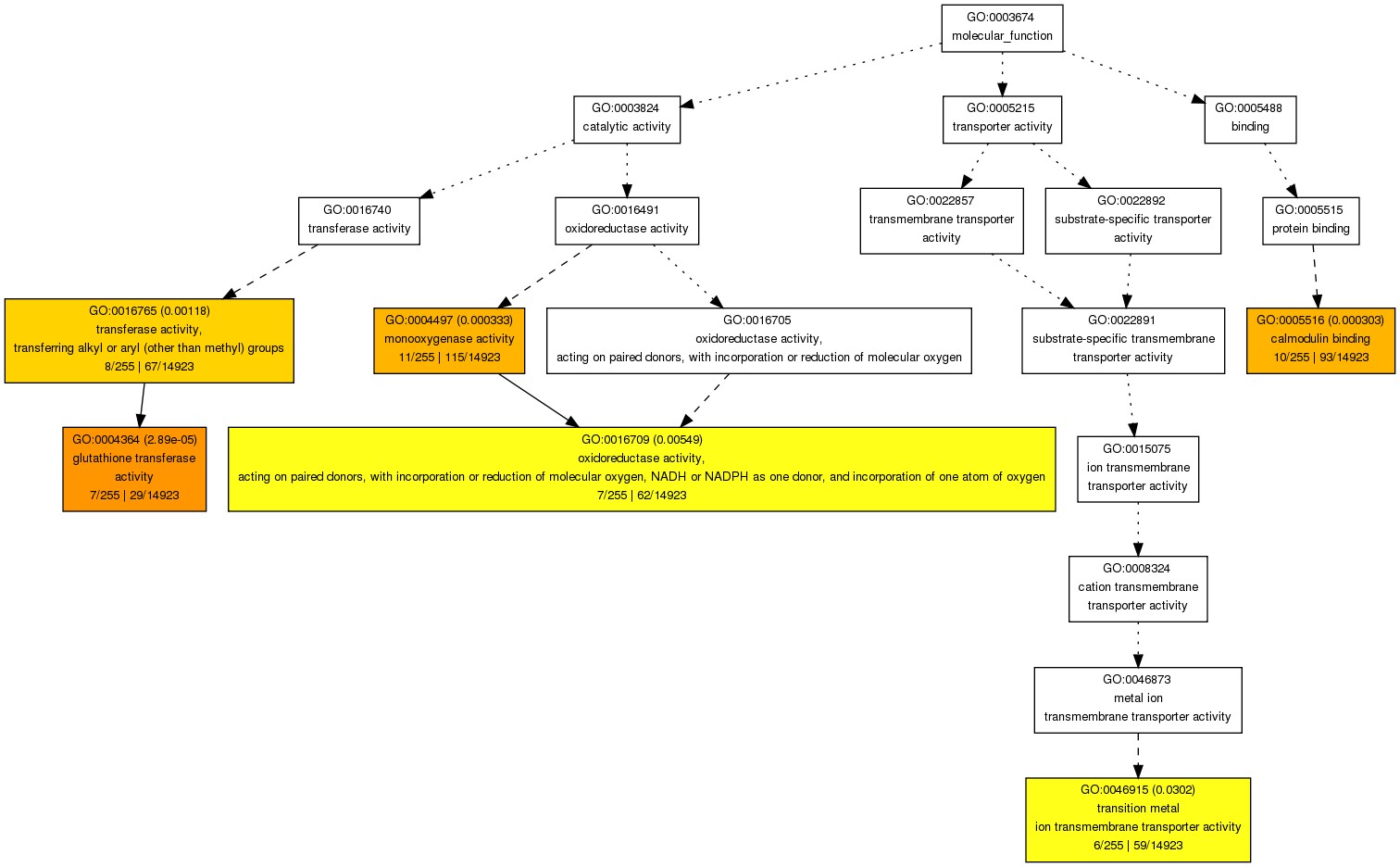


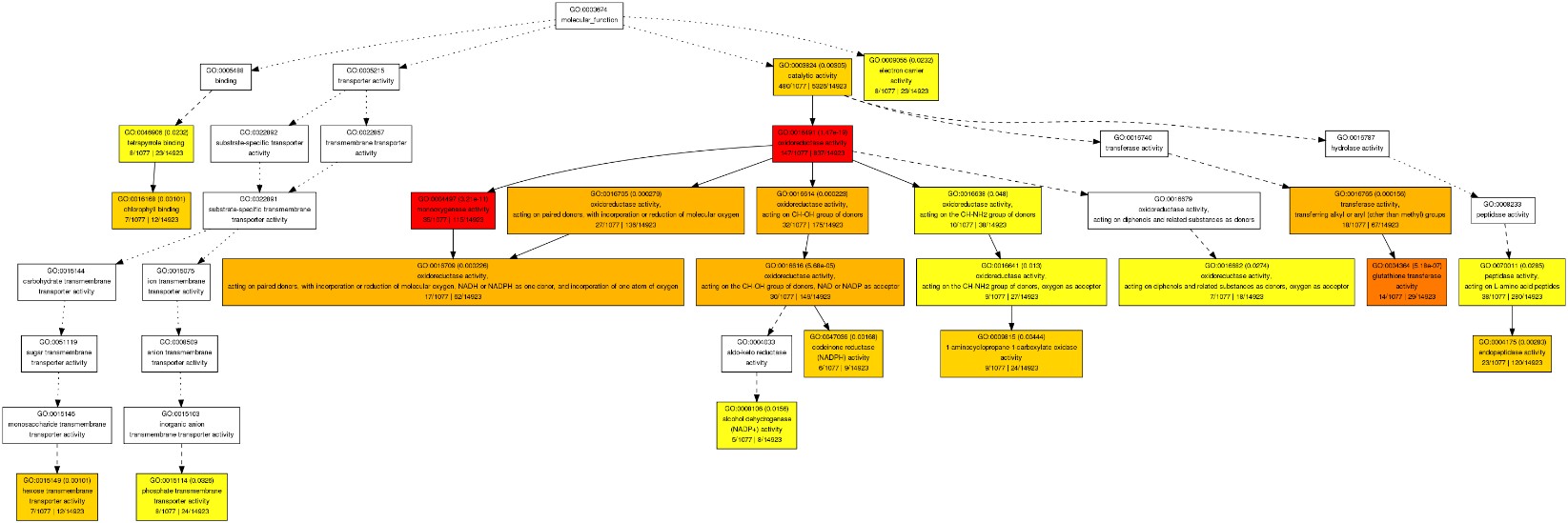


h. UBMD vs USMD


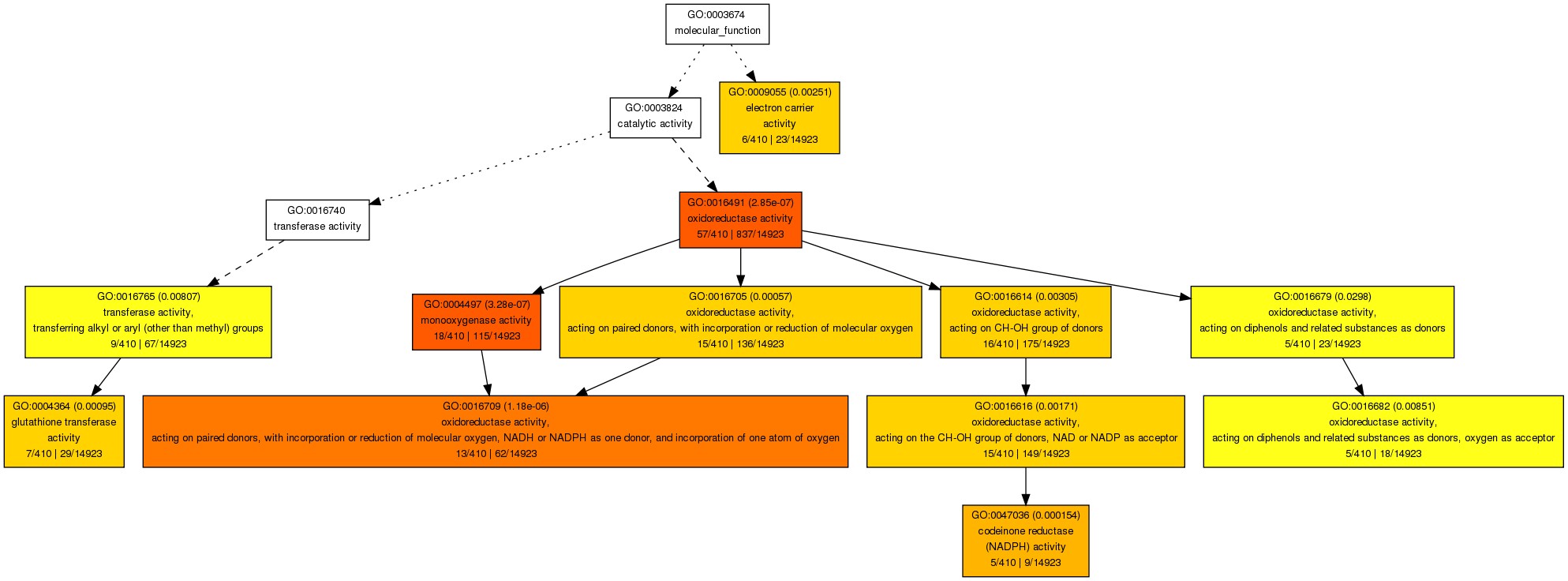


1. Biological Process (Up-regulated)
2. UBA vs USA


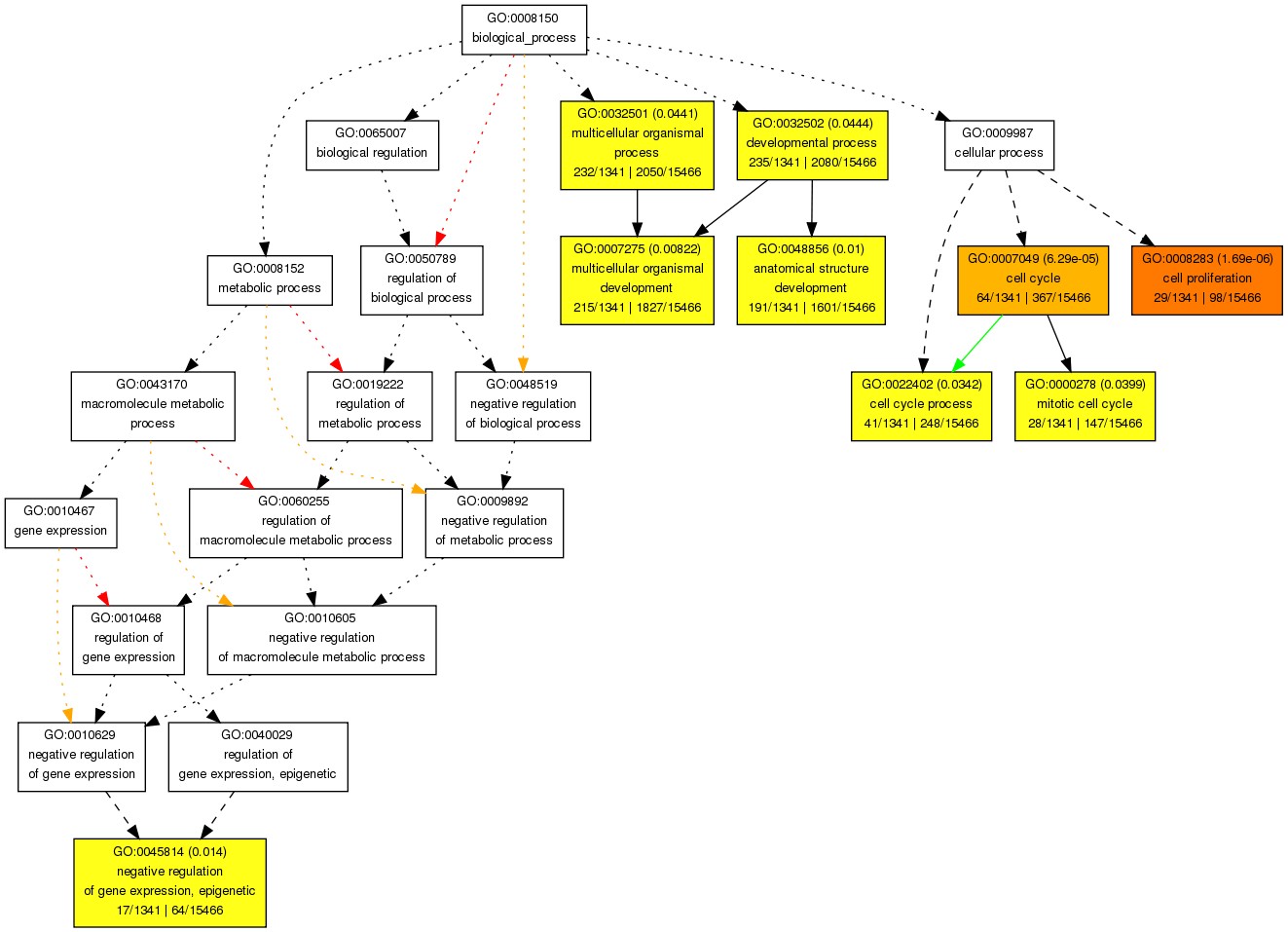


1. UBD vs USD


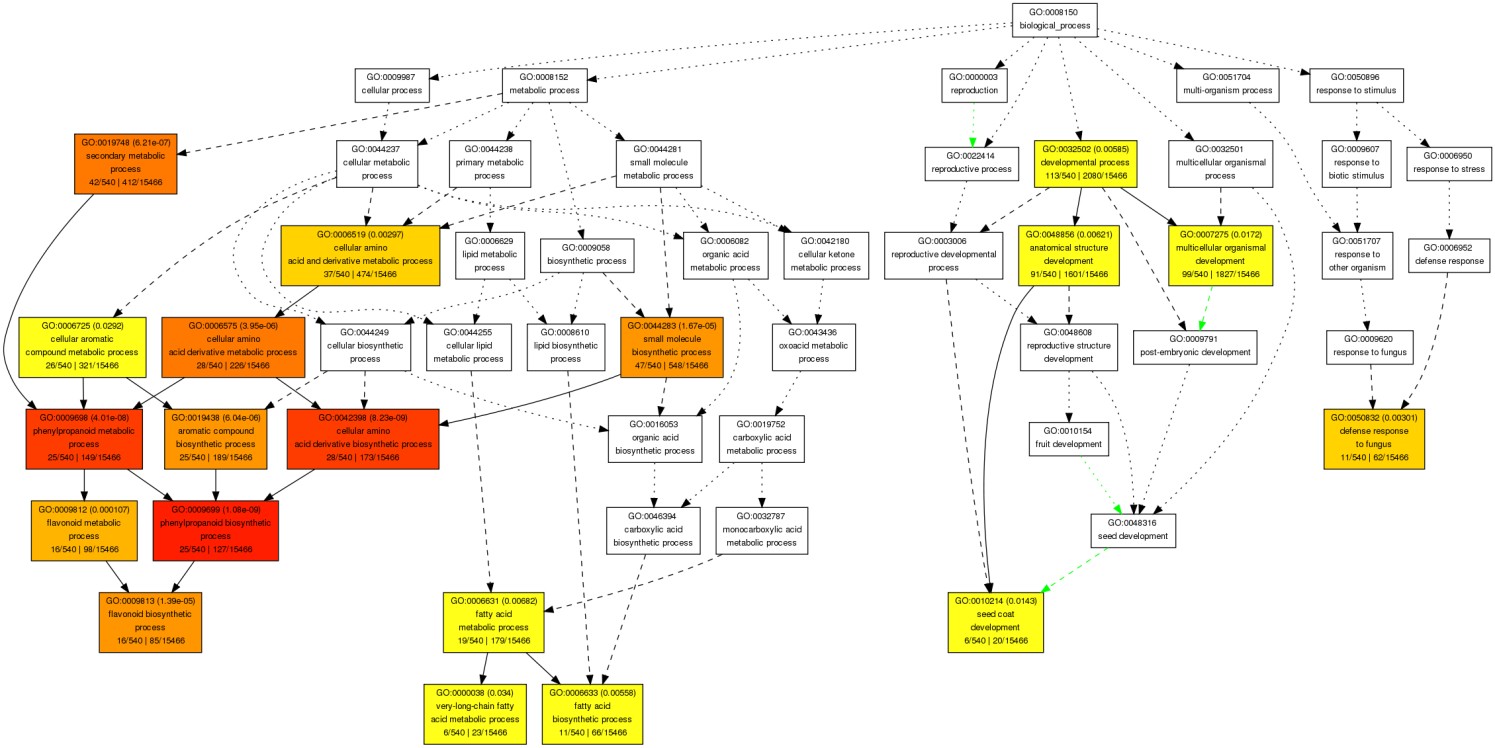


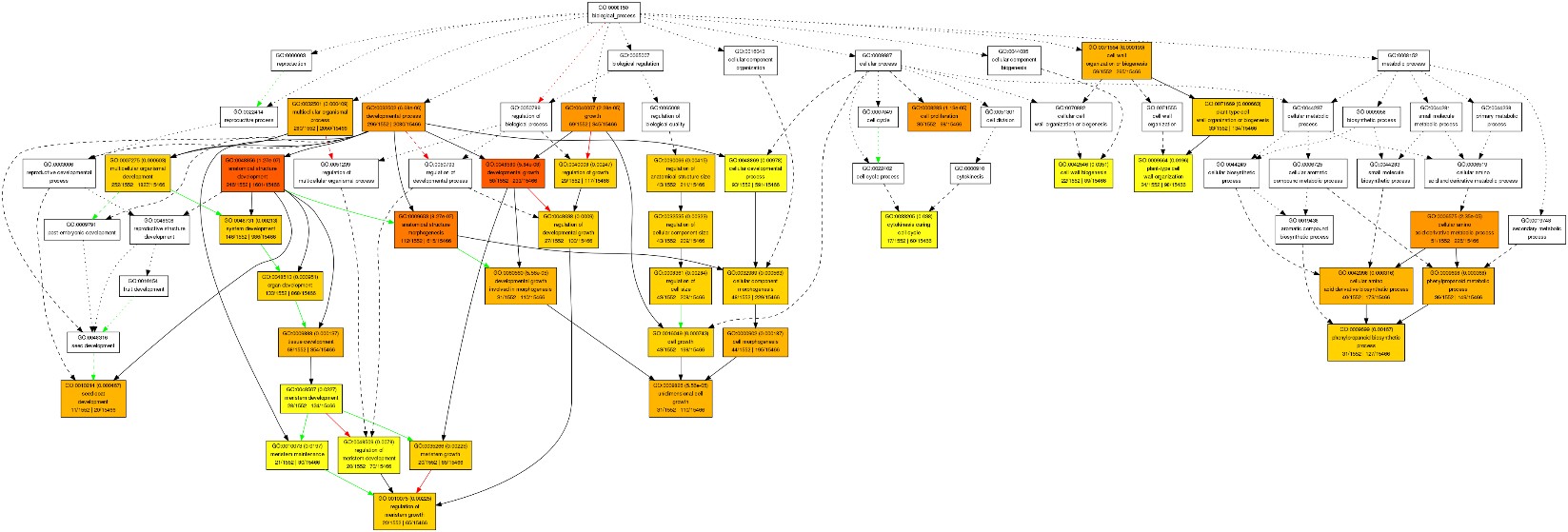


1. UBMD vs USMD


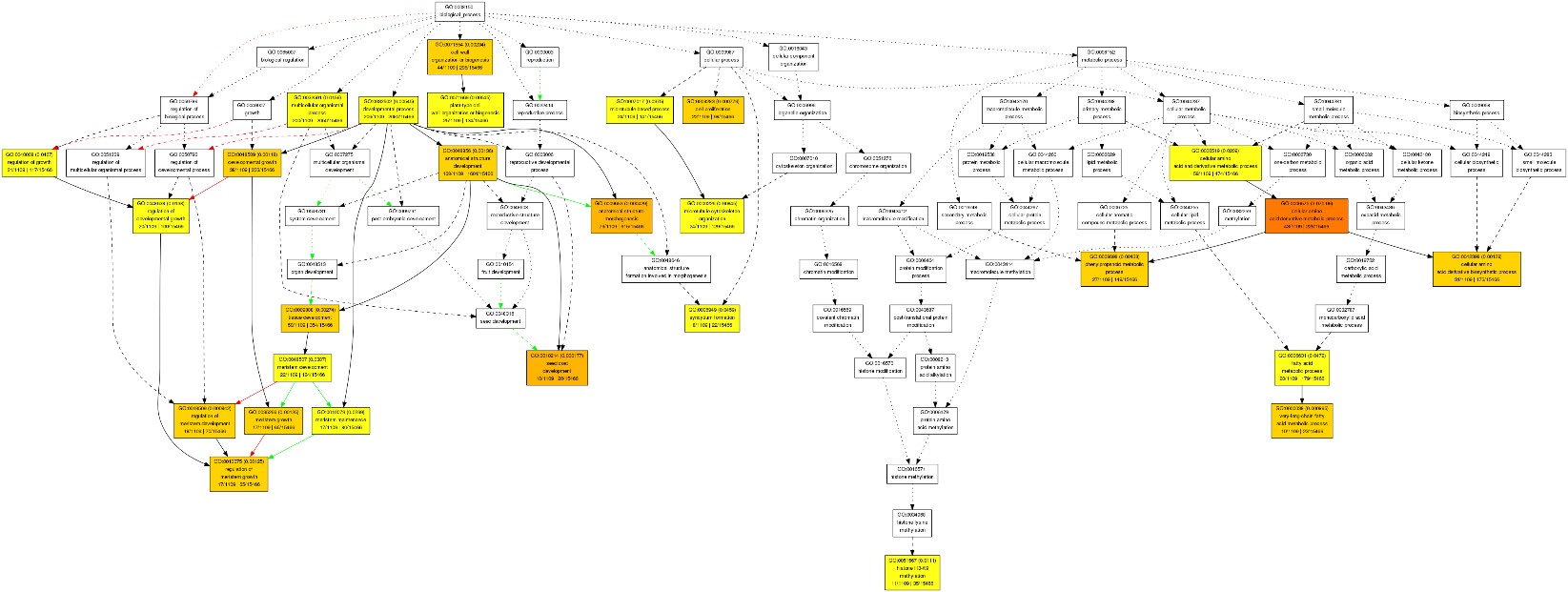


1. UBA vs USA


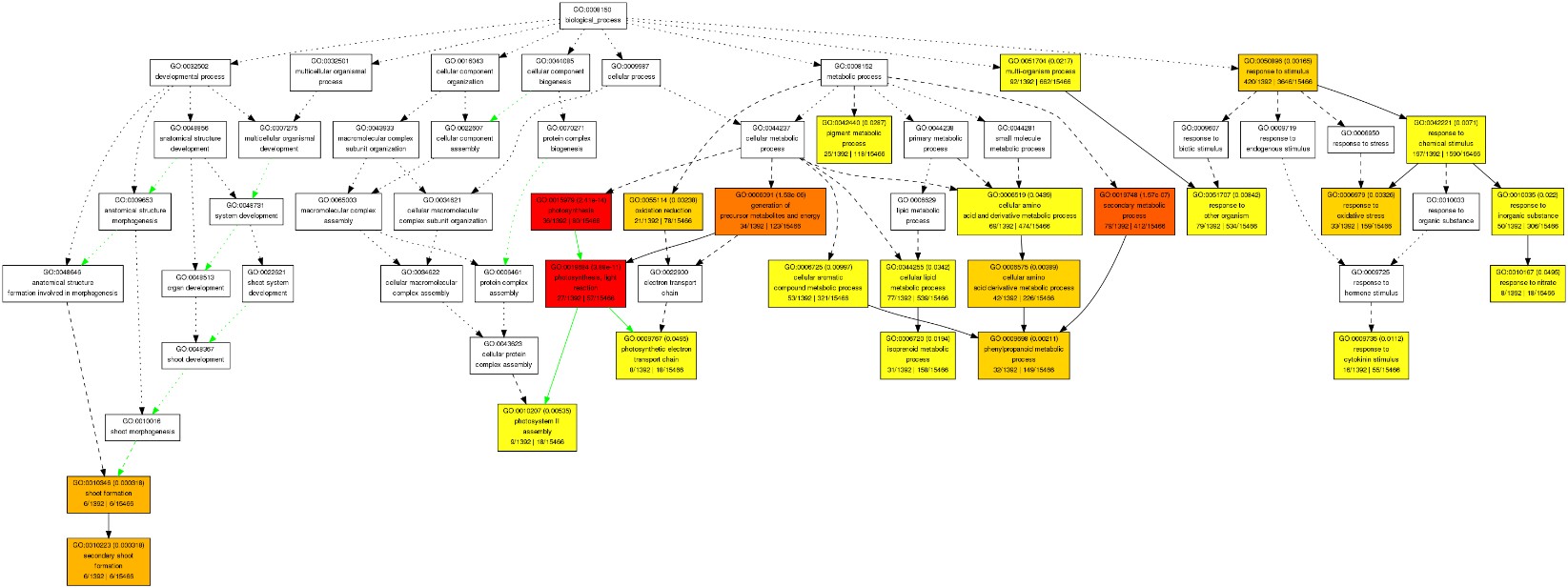


1. UBD vs USD


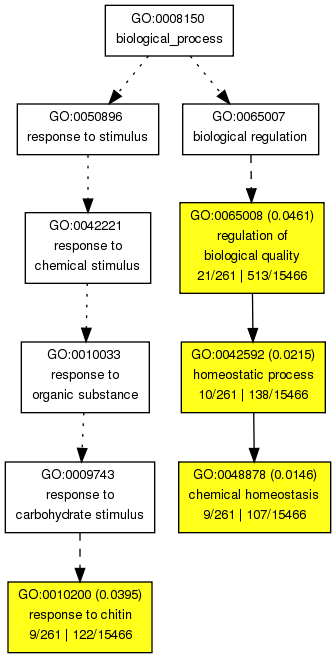


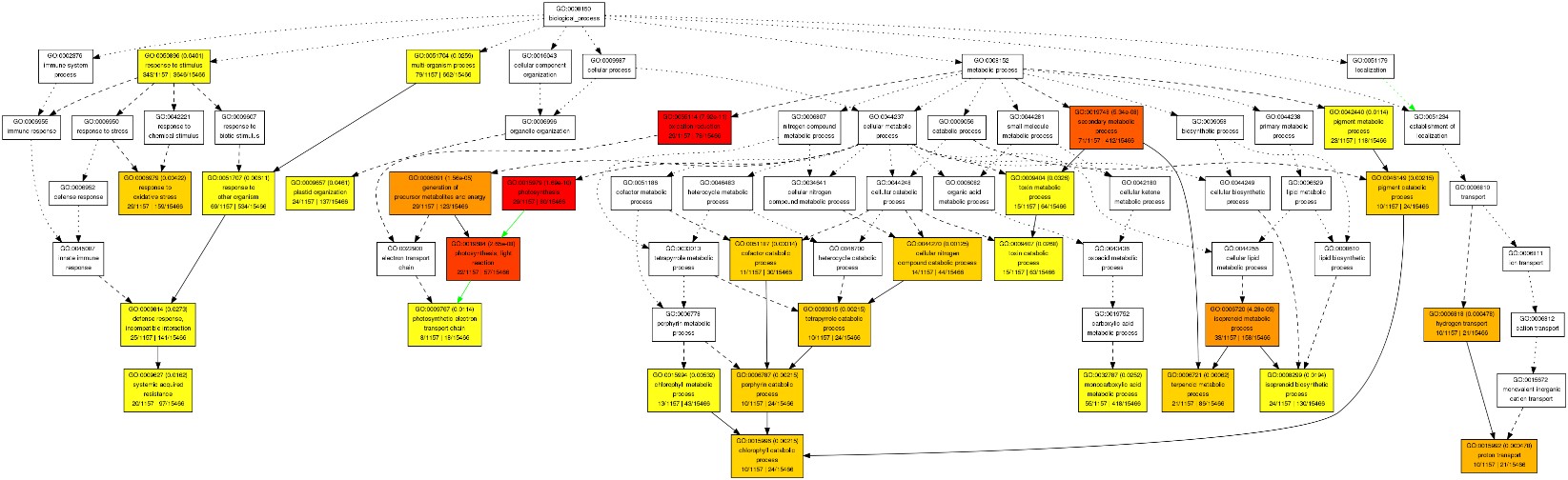


h. UBMD vs USMD


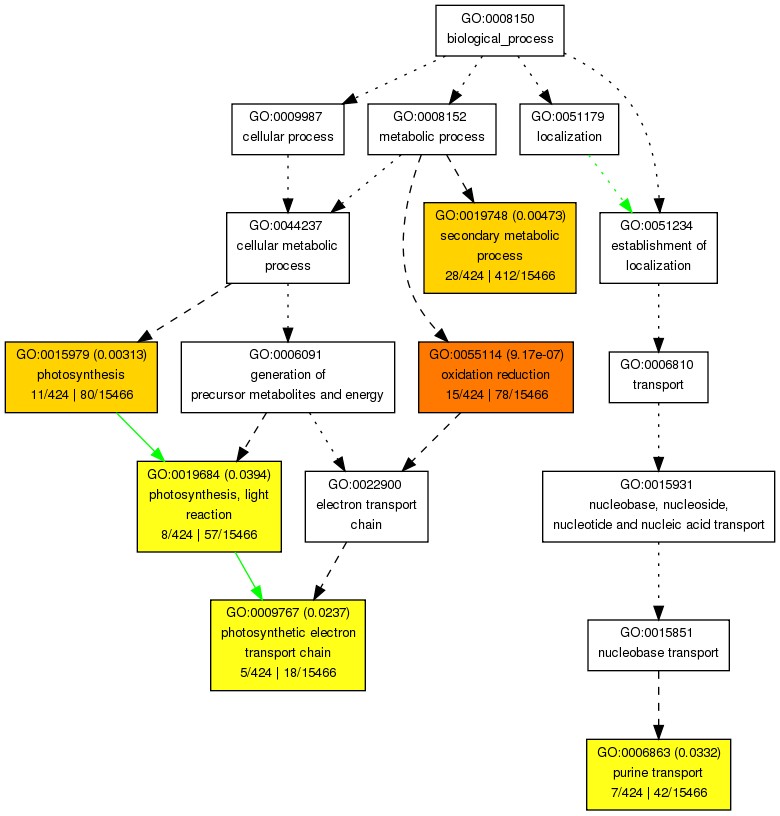


1. Seasonal Comparisons
2. Molecular Function (Up-regulated)
   1. HBA vs HBD


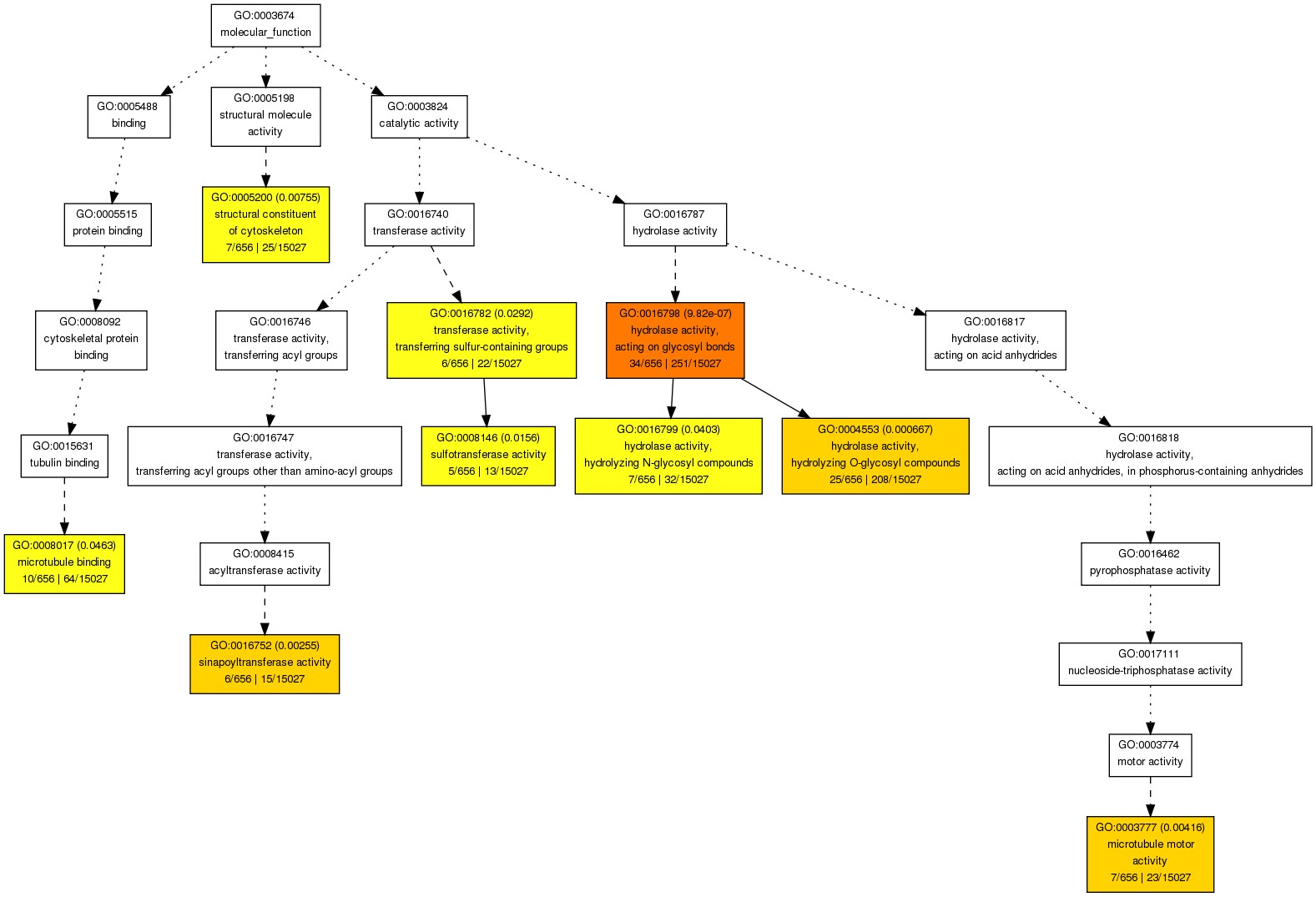


- 1. HBA vs HBMA


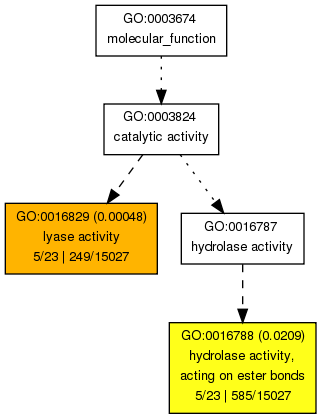


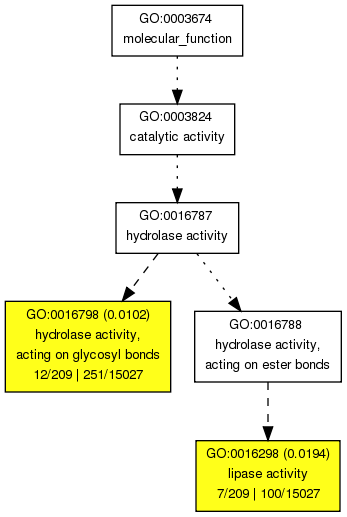


d. HBMA vs HBD


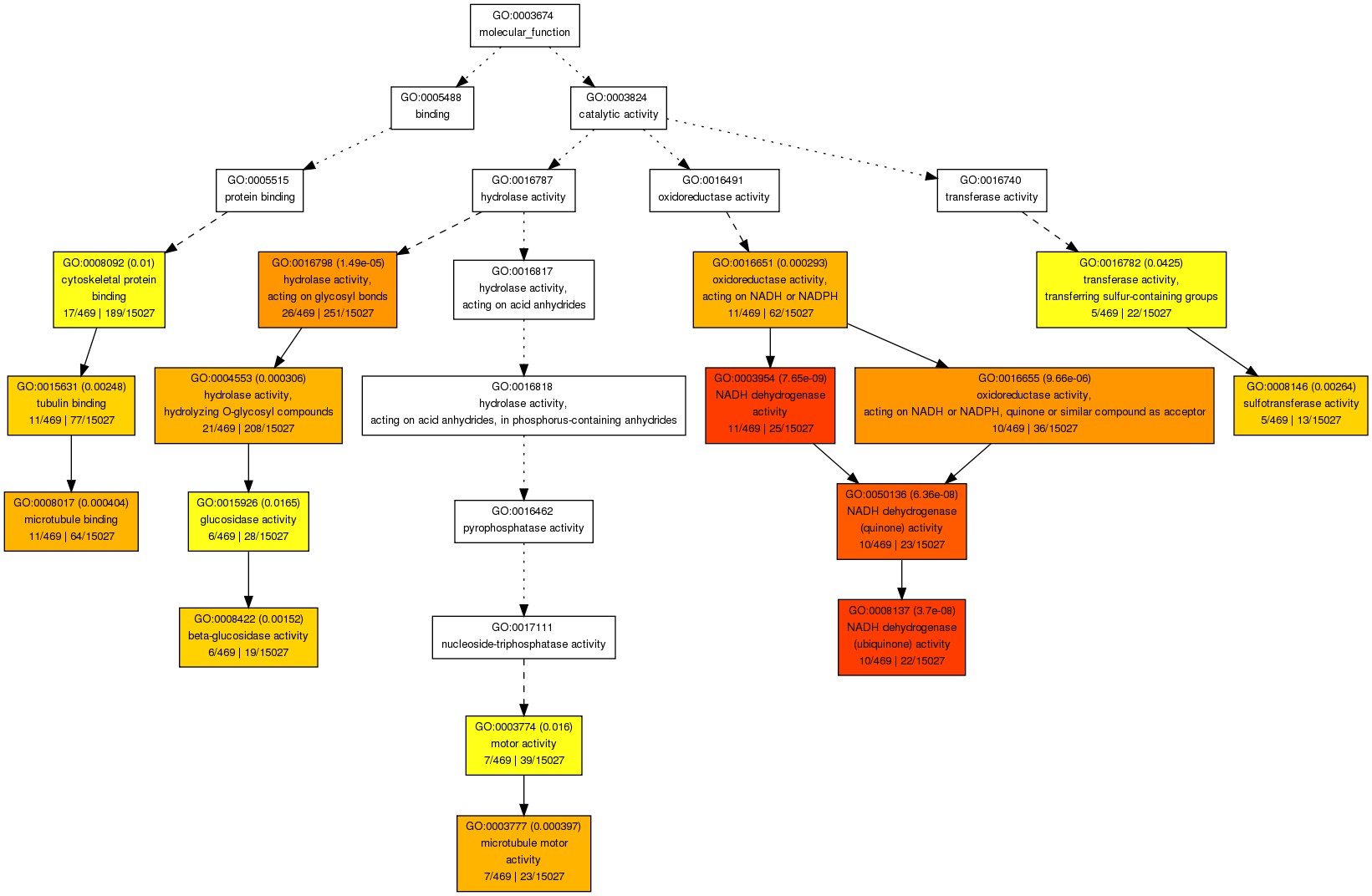


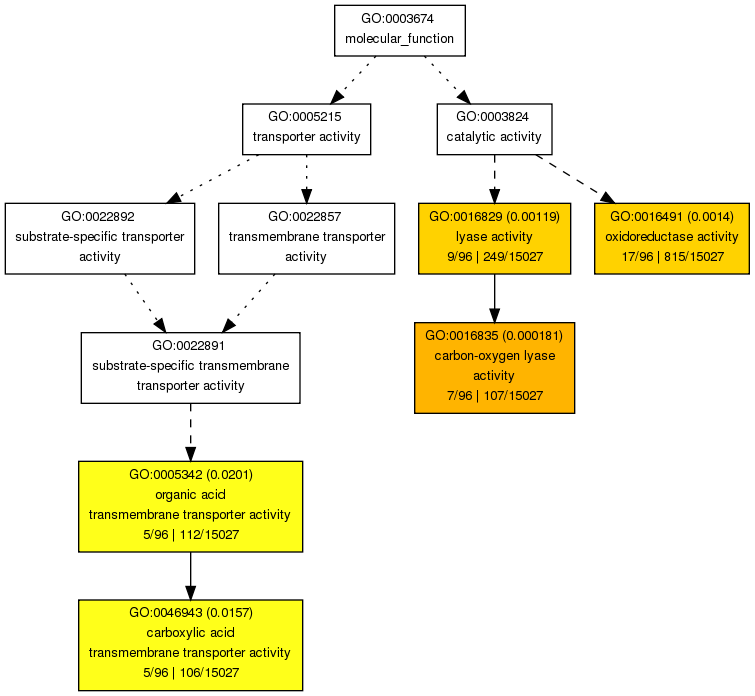


Molecular Function (Down-regulated )

f. HBA vs HBD


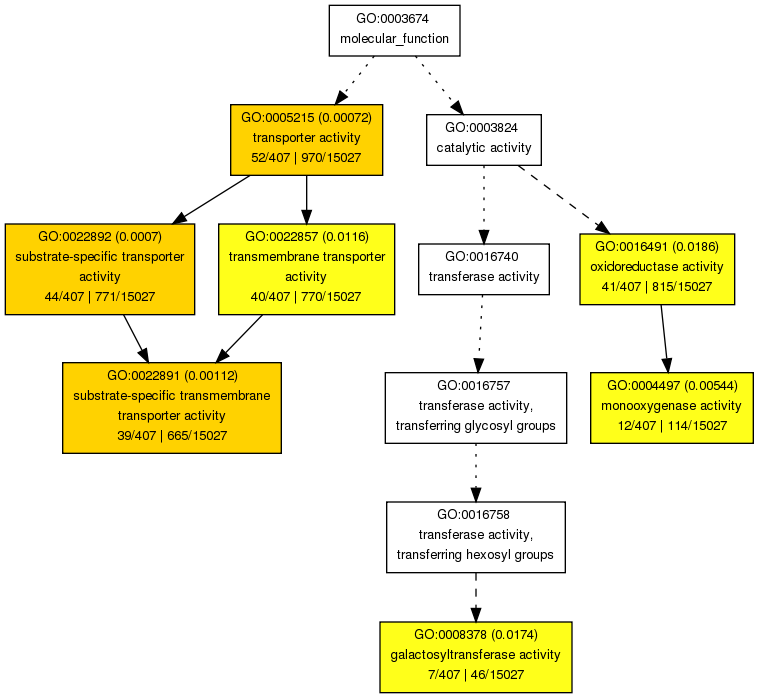


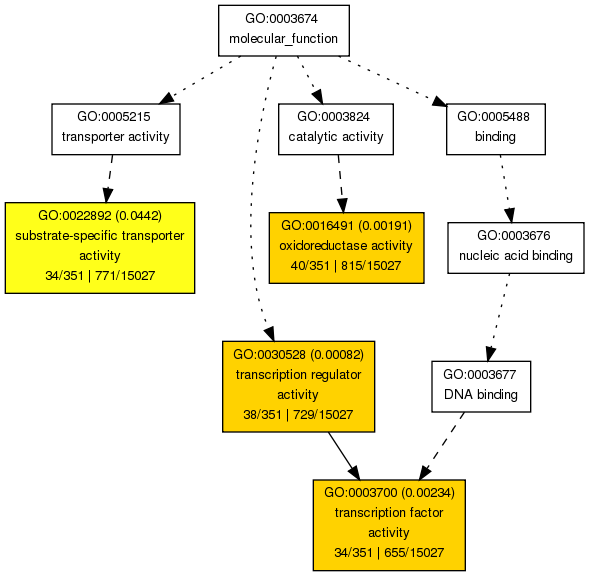


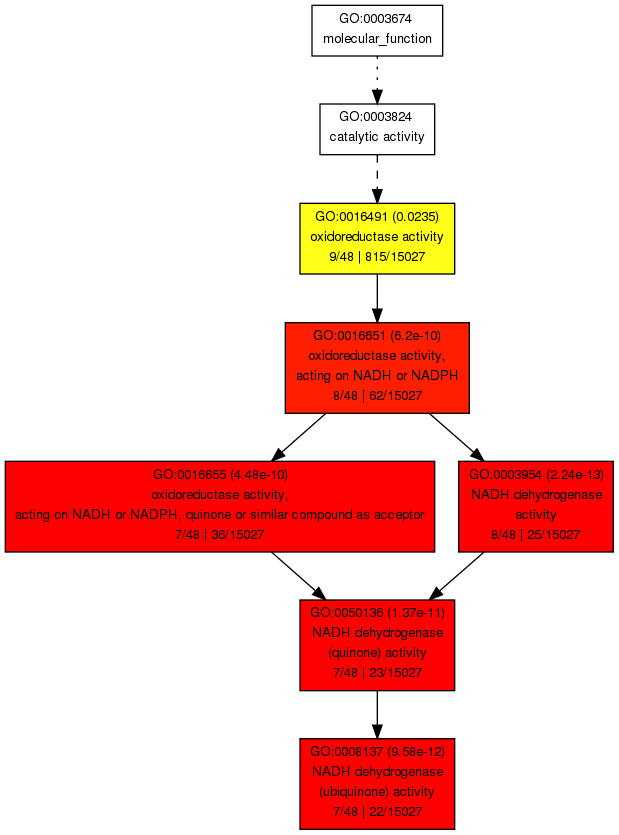
h. HBMA vs HBMD


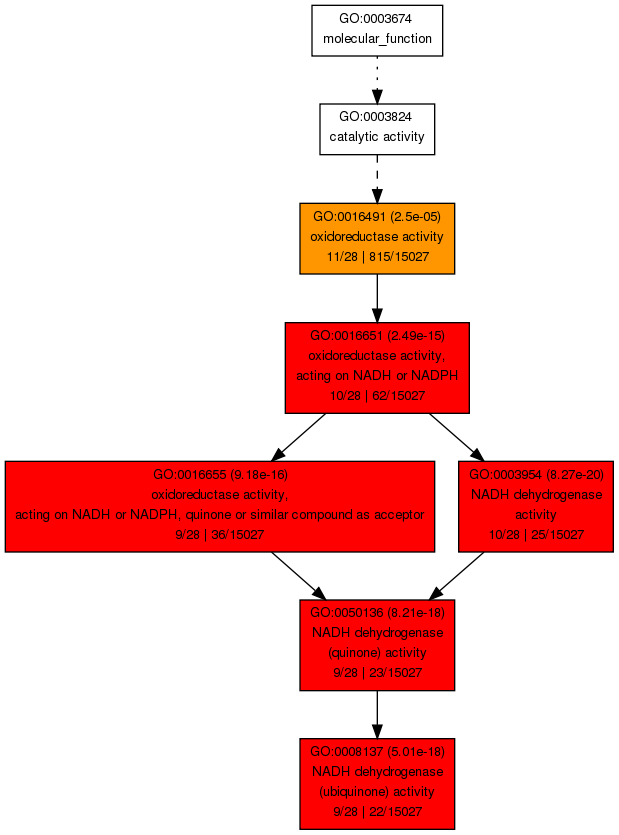


j. HBA vs HBMD


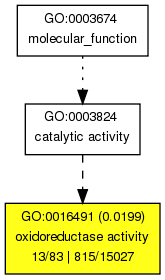


1. Biological Process (Up-regulated)
2. HBA vs HBD


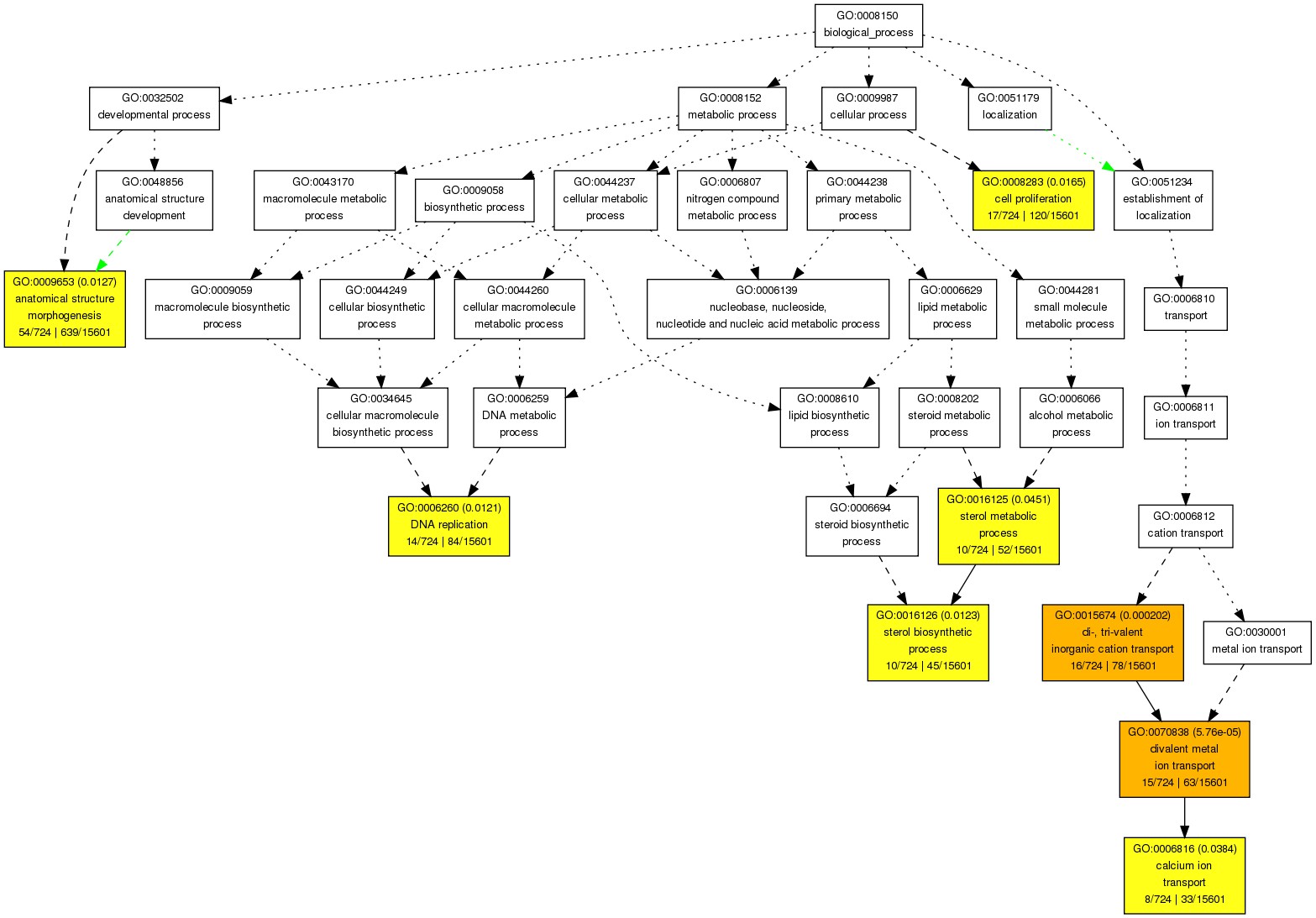


1. HBMA vs HBD


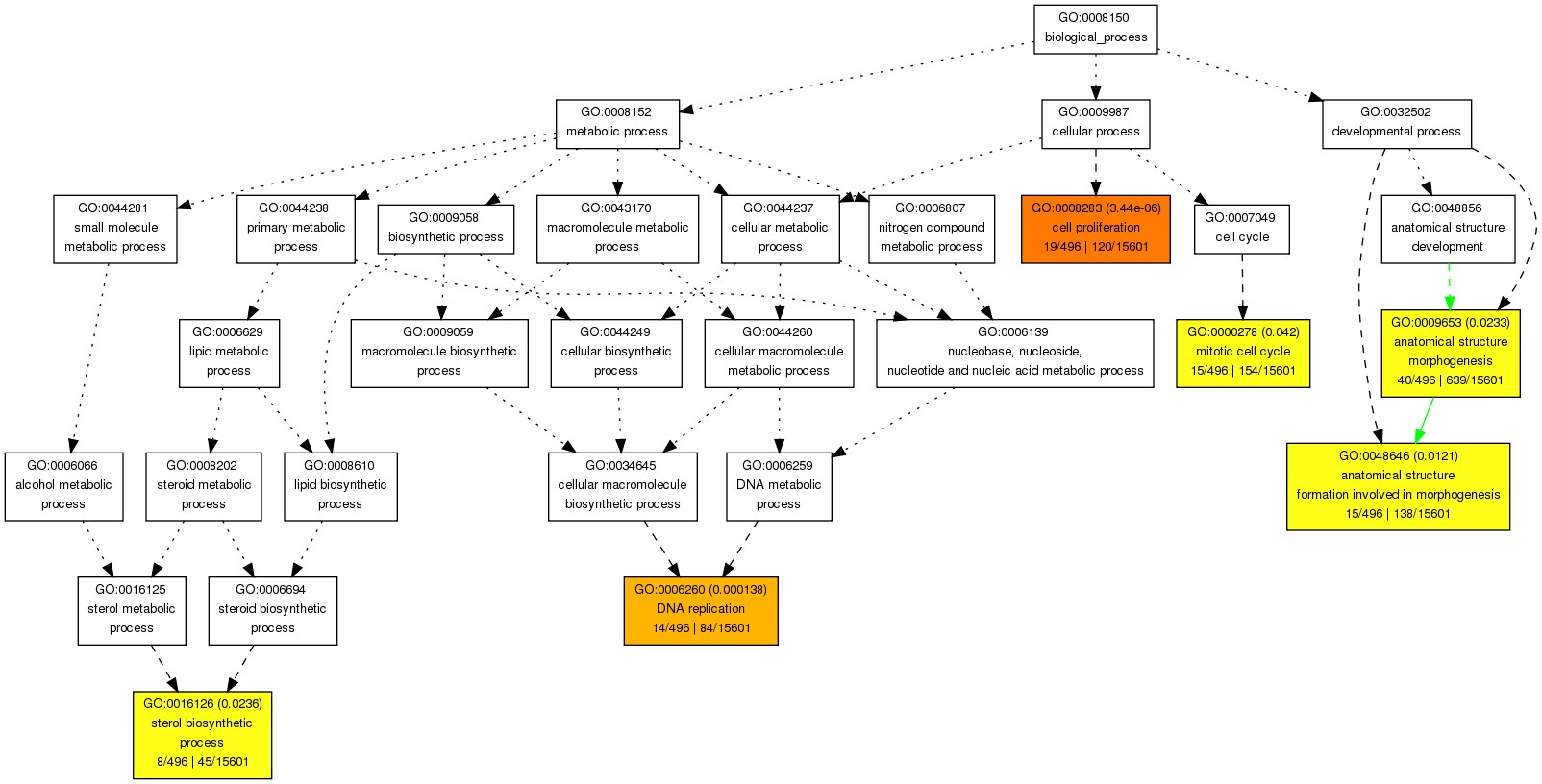


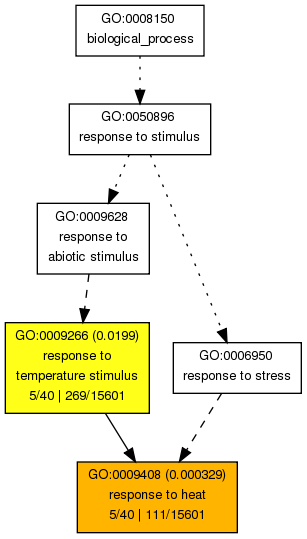


Biological Process (Down-regulated)

1. HBA vs HBMA


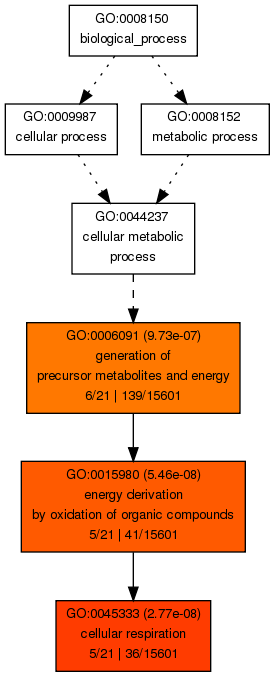


1. HBA vs HBD


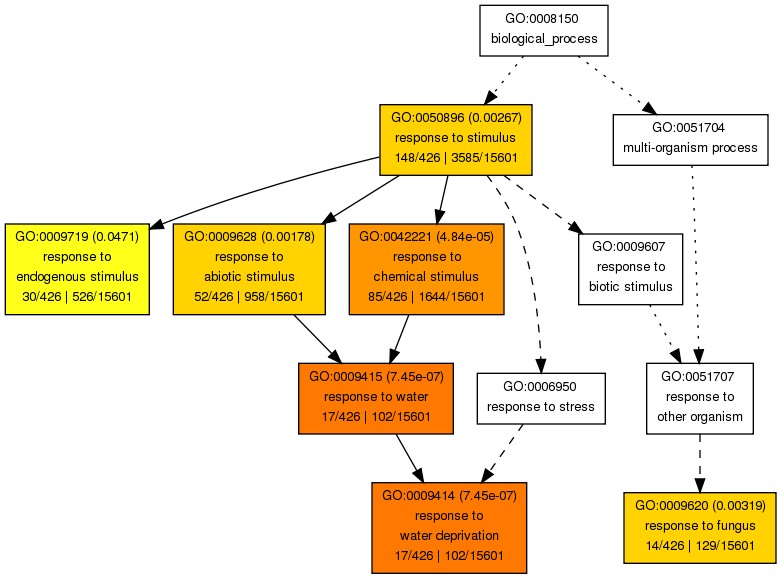


1. HBMD vs HBD


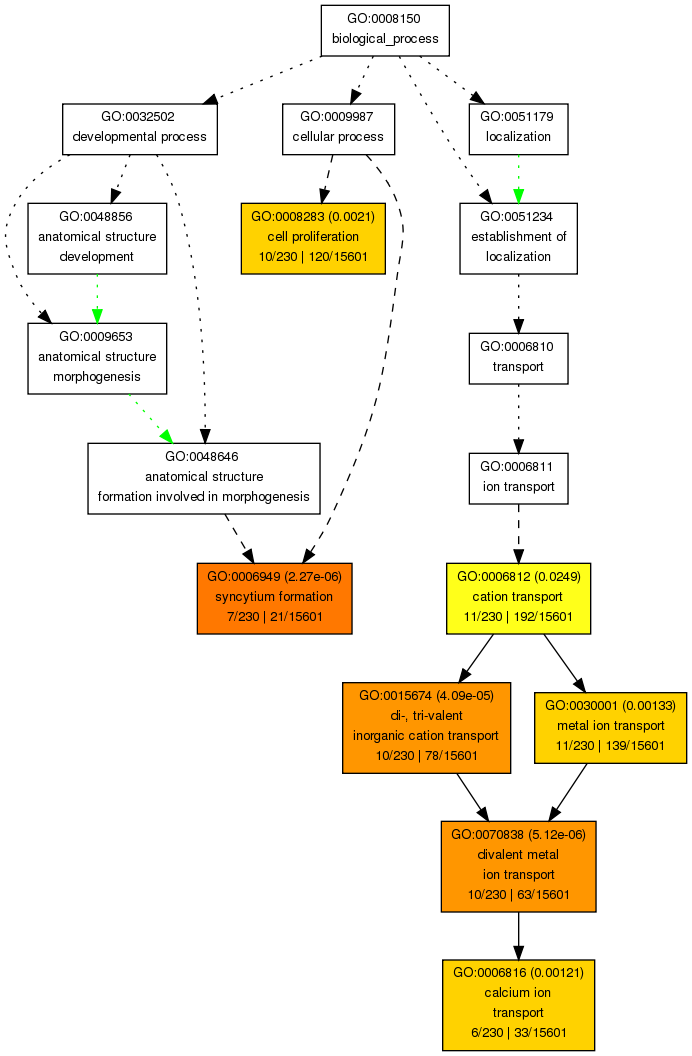


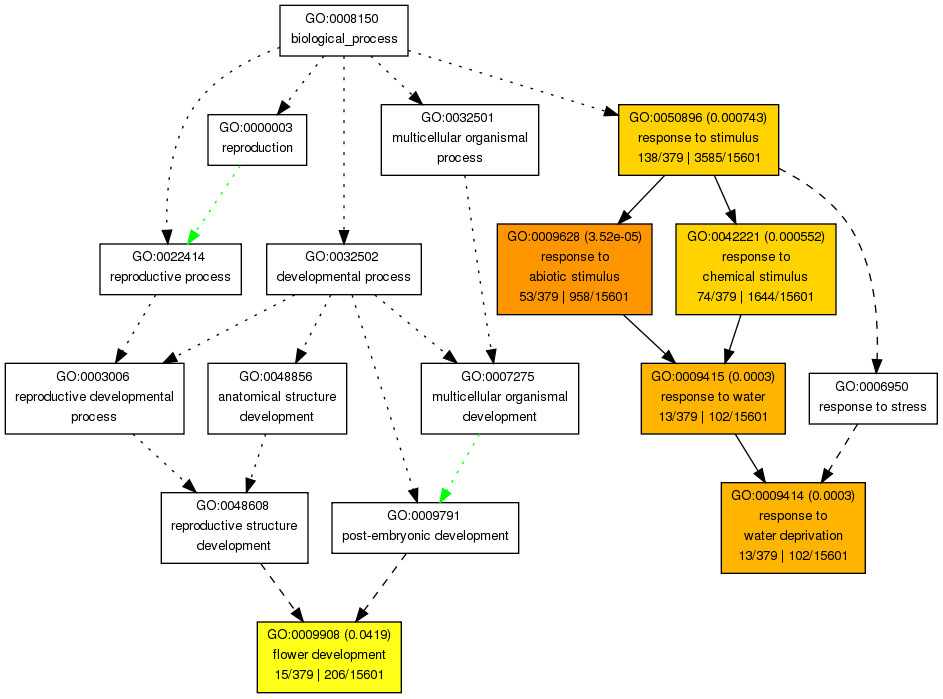


h. HBMA vs HBMD


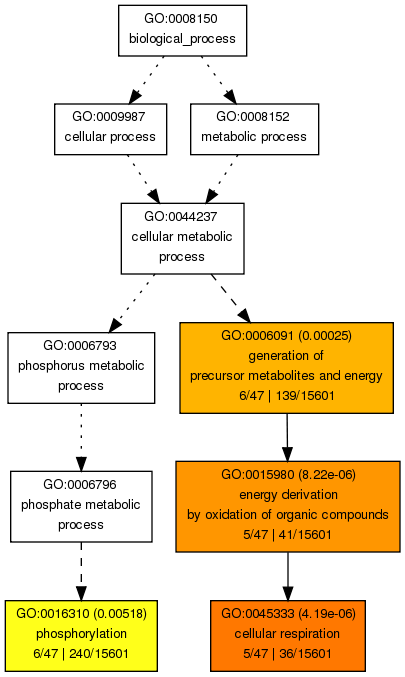


1. Molecular Function (Up-regulated)
2. TBA vsTBD

1. TBMD vsTBD

1. TBA vs TBMD

1. TBMD vsTBD

1. TBA vs TBMD

h. TBA vs TBD

1. Biological Process (Up-regulated)
2. TBA vs TBD

1. TBMD vs TBD

d. TBMA vs TBD

Biological Process (Down-regulated)

1. TBMA vs TBD

1. TBMD vs TBD

g. TBA vs TBD

1. Molecular Function (Up-regulated)
   1. UBA vs UBD

- 1. UBMD vs UBD

d. UBMA vs UBD

Molecular Function (Down-regulated)

f. UBA vs UBMD

h. UBMA vs UBD

1. Biological Process (Up-regulated)

a. UBA vs UBD

c. UBMA vs UBD

Biological Process (Down-regulated)

e. UBA vs UBMA

1. UBA vs UBMD

- 1. UBMA vs UBD

**Supplementary figure 4** GO enrichment analysis of differentially expressed genes (DEGs) during developmental (A; I-VI) and seasonal (B; I-VI) comparisons in three different tea cultivars.
